# Probing Halogen Bonds by Scalar Couplings

**DOI:** 10.1021/jacs.1c04477

**Published:** 2021-07-08

**Authors:** Bono Jimmink, Daniel Sethio, Lotta Turunen, Daniel von der Heiden, Máté Erdélyi

**Affiliations:** Department of Chemistry−BMC, Uppsala University, SE-75123 Uppsala, Sweden

## Abstract

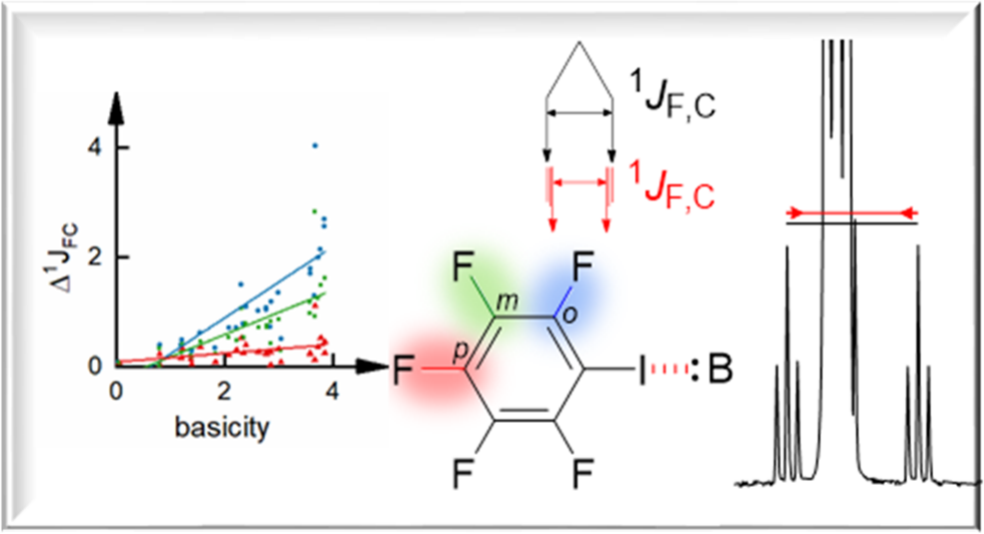

As halogen bonding is a weak, transient interaction, its description in solution is challenging. We demonstrate that scalar coupling constants (*J*) are modulated by halogen bonding. The binding-induced magnitude change of one-bond couplings, even up to five bonds from the interaction site, correlates to the interaction strength. We demonstrate this using the NMR data of 42 halogen-bonded complexes in dichloromethane solution and by quantum chemical calculations. Our observation puts scalar couplings into the toolbox of methods for characterization of halogen bond complexes in solution and paves the way for their applicability for other types of weak σ-hole interactions.

## Introduction

The halogen bond (XB) is a net attractive interaction of a polarized halogen and a Lewis base.^1^ It has lately gained applications, for instance, in supramolecular chemistry,^2,3^ material sciences,^4−6^ drug design,^7−9^ and organic synthesis.^10−13^ Being a weak interaction, its detection and quantification in solution are challenging. Besides UV, IR, and ITC studies, complexation-induced NMR chemical shift alterations have typically been used for the characterization of halogen-bonded complexes.^14^ NMR is often preferred, as it tolerates high concentrations that are necessary to study weak interactions in solution and as it also provides detailed structural information.^15^ Whereas coupling constants are widely utilized in the elucidation of conformational states, their potential for the detection of halogen bonding in solution has not yet been experimentally evaluated. Recent observation of the geometry and bond strength dependence of the ^1^*J*_Ch,P_ of P=Ch···I-type complexes (where Ch denotes a chalcogen) in the solid state^16−18^ and computational reports on through-bond couplings in exotic systems^19,20^ implicate that scalar couplings ought to be applicable for the characterization of weak interactions.

## Results and Discussion

**Figure 1:**
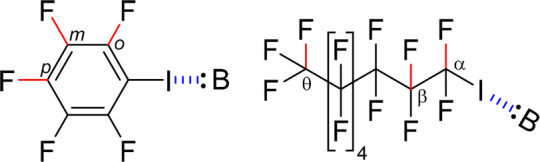
Perfluorinated halogen bond donors in complex with N- and O-donor Lewis bases (B) were used to evaluate the influence of halogen bonding on ^1^*J*_F,C_ scalar couplings near the interactions site.

**Figure 2:**
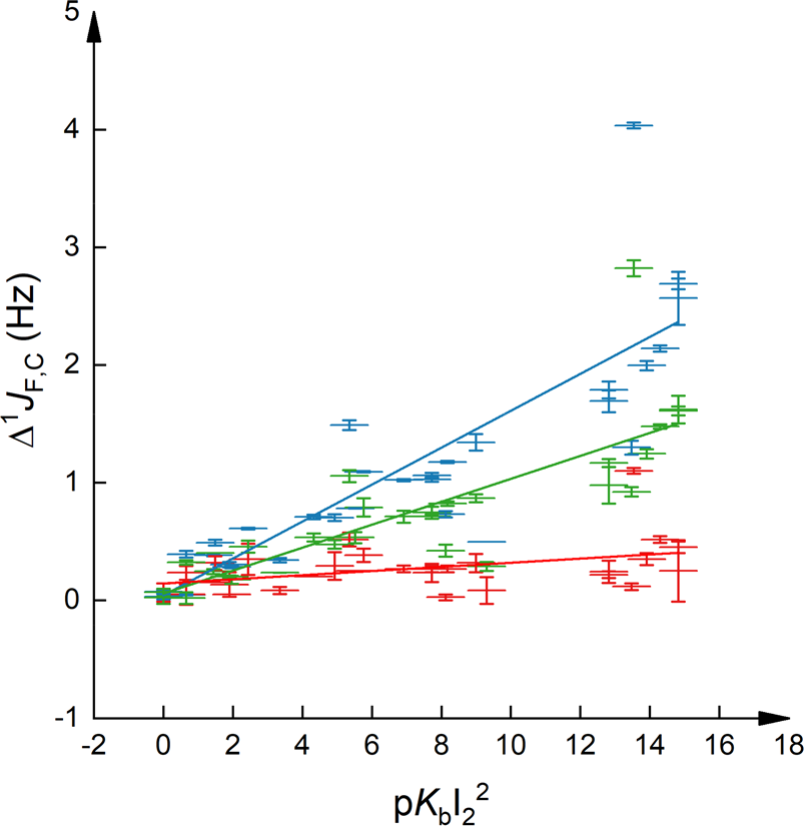
Δ^1^*J*_F,C_ as a function of halogen bond basicity, p*K*_BI2_^2^, observed for the *o*-, *m*-, and *p*-positions of 1-iodopentafluorobenzene upon addition of a variety of Lewis bases. Here, p*K*_BI2_ = 0 refers to a *K* = 1 and not to no binding. Errors are given as standard deviations; a detailed error analysis is given in the Supporting Information. The data corresponding to the *ortho*-position are shown in blue (*R*^2^ = 0.76, slope 0.16), to the *meta* in red (*R*^2^ = 0.16, slope 0.0081), and to the *para* in green (*R*^2^ = 0.66, slope 0.098).

**Figure 3:**
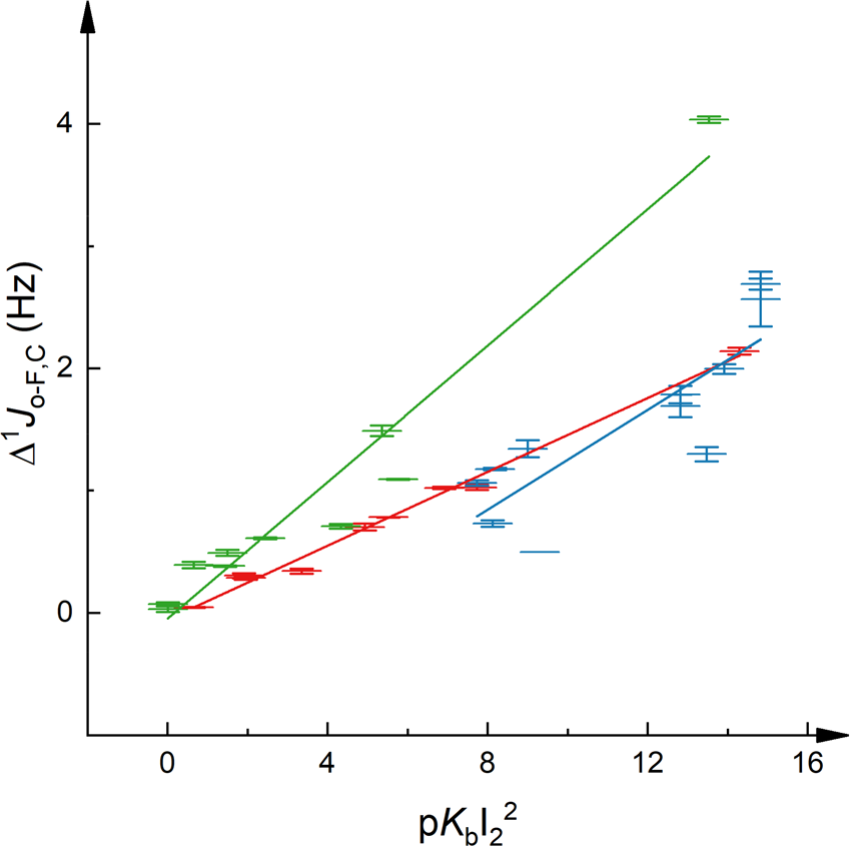
Change of ^1^*J*_F,C_ in the *ortho*-position of iodopentafluorobenzene, Δ^1^*J*_*o*__-F,C_, as a function of the Lewis basicity, p*K*_BI2_^2^, upon complexation to a variety of Lewis bases. Errors are given as standard deviations; p*K*_BI2_ = 0 refers to a *K* = 1. The data corresponding to the pyridines are shown in red (*R*^2^ = 0.99), to amines in blue (*R*^2^ = 0.71), and to *N*-oxides and carbonyls in green (*R*^2^ = 0.95).

Herein, we demonstrate that scalar one-bond couplings (^1^*J*) are modulated by halogen bonding in solution. For this proof of principle study, we chose to detect ^1^*J*_F,C_, as the most commonly used halogen bond donors are perfluorinated.^21^ They are known to form halogen bond complexes in solution^22^ and in the solid state.^17,18,23^ The use of fluorinated halogen bond donors in combination with nonfluorinated Lewis bases diminishes the risk of signal overlaps, which is further supported by the wide, ∼800 ppm, chemical shift scale of ^19^F NMR. Thereto, the magnitude of ^1^*J*_F,C_ can be straightforwardly measured on the ^13^C satellites of well-separated ^19^F NMR signals acquiring standard 1D NMR spectra at natural abundance. A similar approach has earlier been applied, for instance, in the study of stereoelectronic effects using ^1^*J*_C,C_.^24^ Herein, we report the change of ^1^*J*_F,C_ for the *o-*, *m-,* and *p*-positions of 1-iodopentafluorobenzene upon addition of 10 pyridines, 11 aliphatic amines, 4 *N*-oxides, and 6 carbonyl bases, and for the α- and β-positions of 1-iodoperfluorooctane upon addition of 10 pyridine bases (Figure 1 and Figure S1, Supporting Information). To minimize the influence of nonspecific interactions on the obtained data, such as solvent effects, measurements were performed with a 200 mM halogen bond donor in dichloromethane, using 2.5 equiv of the corresponding Lewis base. The coordination induced change in ^1^*J*_F,C_ of the halogen bond donor (Δ^1^*J*_F,C_) as a function of the interacting halogen bond acceptor’s Lewis basicity (p*K*_BI2_) is shown for iodopentafluorobenzene in Figure 2. Here, p*K*_BI2_ is the decimal logarithm of the experimental complexation constant (*K*) of a Lewis base upon interaction with diiodine.^25^ It is referred to as the diiodine scale, and it has been applied as a family-dependent halogen bond basicity scale.^26^ The complexation-induced change in the coupling constant is defined, in analogy to the definition of coordination shifts, as Δ^1^*J*_F,C_ = ^1^*J*_F,C(XB complex)_ – ^1^*J*_F,C(free XB donor)_. Due to the weak nature of halogen bonds,^15,27^ the observed Δ^1^*J*_F,C_s are not only dependent on the enthalpy of the interaction (Δ*H*) but also on the molar fraction of the halogen bond complex that is formed (*K*). The linear correlation of Δ^1^*J*_F,C_ with p*K*_BI2_^2^ is thus due to the linear involvement of p*K* and Δ*H* in Δ*G.* A similar linear relationship to the above was also observed for the binding-induced chemical shift changes, Δδ_F_, to p*K*_BI2_^2^ (Figures S4, S5, Supporting Information). Upon addition of Lewis bases to iodopentafluorobenzene, we observed a positive correlation between the ^1^*J*_F,C_s of the halogen bond donor and the strength of the halogen bond acceptor (Figure 2). The magnitude of Δ^1^*J*_F,C_ follows the expected |Δ^1^*J*_*o*__-F,C_| > |Δ^1^*J*_*p*__-F,C_| > |Δ^1^*J*_*m*__-F,C_| order, consistent with the order of chemical shift changes of the fluorines, that is |Δδ_*o*__-F_| > |Δδ_*p*__-F_| ≈ |Δδ_*m*__-F_| (Figure S4, Supporting Information). The observation that weak halogen bonds are detectable on the magnitude of scalar couplings even up to five bonds from the interaction site is worth noting. The correlation of Δ^1^*J*_F,C_ to p*K*_BI2_^2^, as expressed by the coefficient of determination (*R*^2^), is stronger for the *o*- and *p*-Δ^1^*J*_F,C_ than for the *m*-Δ^1^*J*_F,C_. It is also stronger for halogen bond acceptor families that induce larger Δ^1^*J*_F,C_, which results in a steeper slope (Figure 3). The steepness of the slopes follows the expected Δ^1^*J*_*o*-F,C_ > Δ^1^*J*_*p*-F,C_ > Δ^1^*J*_*m*-F,C_ order, analogous to the corresponding correlations of the chemical shifts (Figure S5, Supporting Information). However, the correlation of Δ^1^*J*_*m*-F,C_ to p*K*_BI2_^2^ is weak (*R*^2^ = 0.16), and the slopes of Δ^1^*J*_*p*-F,C_ and Δ^1^*J*_*m*-F,C_ differ (Figure 2), whereas Δδ_*m*-F_ shows a better correlation to p*K*_BI2_^2^ and the slopes of Δδ_p-F_ and Δδ_*m*-F_ are comparable (Figures S4, S5, Supporting Information). This suggests that ^1^*J*_*m*_ is a less good parameter for the description of halogen bond strength than ^1^*J*_*o*_ and ^1^*J*_*p*_. When comparing the trends in Δ^1^*J*_F,C_ and Δδ_F_, the *J*-couplings are overall more sensitive to the distance from the binding site as compared to the chemical shift, δ, of the same fluorine.

Next, we analyzed the influence of halogen bonding on the magnitude of ^1^*J* using different subsets of bases representing pyridines, amines, *N*-oxides, and carbonyls (Figure 3). Comparing Δ^1^*J*_*o*__-F,C_s, strong correlations, *R*^2^ ≥ 0.95, are observed for pyridines, *N*-oxides, and carbonyls, whereas those for amine halogen bond acceptors were weaker, *R*^2^ = 0.71. This is not unexpected, as the iodine basicity scale is known to be subset sensitive.^28^ However, the correlation markedly improves upon selectively correlating the ^1^*J*-couplings of primary, secondary, and tertiary alkyl-amines (Figure S6, Supporting Information), which suggests the importance of steric effects. This is further corroborated by the decreasing strength of correlation observed in the order primary > secondary > tertiary amines, when investigated selectively.

**Figure 4:**
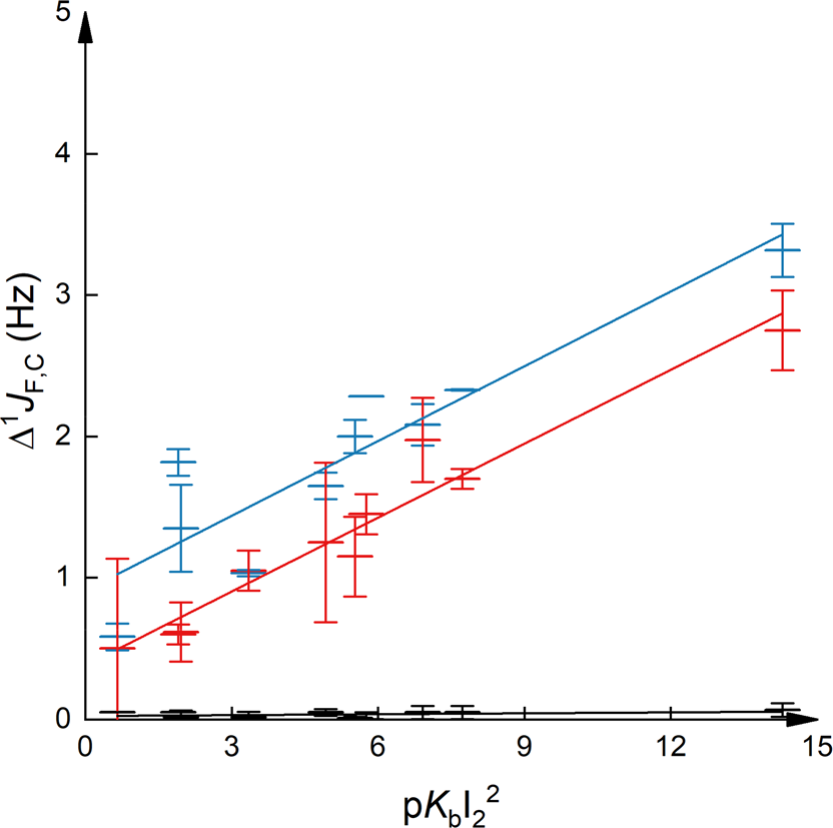
Δ^1^*J*_F,C_ of 1-iodoperfluorooctane as a function of the squared iodine basicity (p*K*_BI2_^2^) of the interacting Lewis base. Here, p*K*_BI2_ = 0 refers to *K* = 1 and not to no binding. Errors are given as standard deviations. The data corresponding to the α-position are given in blue (*R*^2^ = 0.80), to the β-position in red (*R*^2^ = 0.94), and to the θ-position in black (*R*^2^ = 0.03).

To explore the generality of the influence of a weak halogen bond on the magnitude of scalar couplings, we also assessed the complexes of an aliphatic halogen bond donor. As a model compound, we chose 1-iodoperfluorooctane (Figure 1), because it has a similar halogen bond donor strength to 1-iodopentafluorobenzene.^27^ Its complexes with 10 pyridine bases provide a linear correlation of Δ^1^*J*_F,C_, and of Δδ_F_, for the α- and β-fluorines toward p*K*_BI2_^2^ (Figure 4). The reference θ-fluorine, nine bonds away from the interaction site, does not experience Δ^1^*J*_F,C_ upon halogen bond formation. As a reference experiment to ensure that the observed Δ^1^*J*_F,C_ at the α- and β-positions were due to halogen bonding, we evaluated whether a Δ^1^*J*_F,C_ is measurable upon addition of 2.5 equiv of *n*-pentane to 1-iodoperfluorooctane. As expected, the ^1^*J*_F,C_ at the α- and β-positions did not experience any significant changes (for details, see the Supporting Information). The ^1^*J*_F,C_ of the reference θ-position turned out to be a feasible reporter on polarity changes of the environment, which are independent of halogen bonding. Hence we observed 2.1 Hz alteration of ^1^*J*_F,C_ in the θ-position upon addition of pentane, whereas no significant changes were seen in this position upon addition of halogen bond acceptors (Figure 4). The lack of Δ^1^*J*_F,C_ at the α- and β-positions upon addition of water confirmed that moisture does not have a significant effect, most likely due to the weak Lewis basicity of water as compared to the halogen bond acceptors used in this study. Overall, we observed similar trends for 1-iodoperfluorooctane to those seen for the complexes of 1-iodopentafluorobenzene. Hence, the Lewis basicity (p*K*_BI2_) of the halogen bond acceptor positively correlates with the Δ^1^*J*_F,C_ (and the Δδ_F_) observed on the halogen bond donor upon halogen bonding. The magnitude of these changes decreases with an increasing number of bonds between the halogen bond donor iodine and the observed C–F bond. The slope of the correlation of ^1^*J*_F,C_ to p*K*_BI2_ is comparable for the α- and the β-positions (Figure 4). Conversion of the p*K*_BI2_ values into specific p*K*_B_(C_8_F_17_I) and p*K*_B_(C_6_F_5_I) values would be possible upon correction to the binding affinities (*K*), as described by Laurence et al.^25,26^ Whereas this would not alter the linearity of the correlation, this might improve the quantitativity of the graphs, supporting their use for predictive purposes.

To rationalize the experimental findings, we have computationally studied the influence of the halogen bond on the magnitude of nearby Δ^1^*J*_F,C_s. The electron density at the C–I···B bond critical points (ρ_I···N_) was chosen as the indicator for the interaction strength. This has previously been used in the topological analysis of the electron density distribution at the description of distinct interactions^29−31^ and correlates with the Δ*G* of the interaction when other effects, such as steric crowding, are negligible. Our DFT computations (B3LYP-D3/aug-cc-pVTZ/PCM(CH_2_Cl_2_)) indicate that the calculated ^1^*J*_o-F,C_ of 1-iodopentafluorobenzene linearly correlates to the electron density at the bond critical point (ρ_I···N_, *R*^2^ = 0.97) as well as to the halogen bond binding energy (*E*_XB_, *R*^2^ = 0.80).

**Figure 5:**
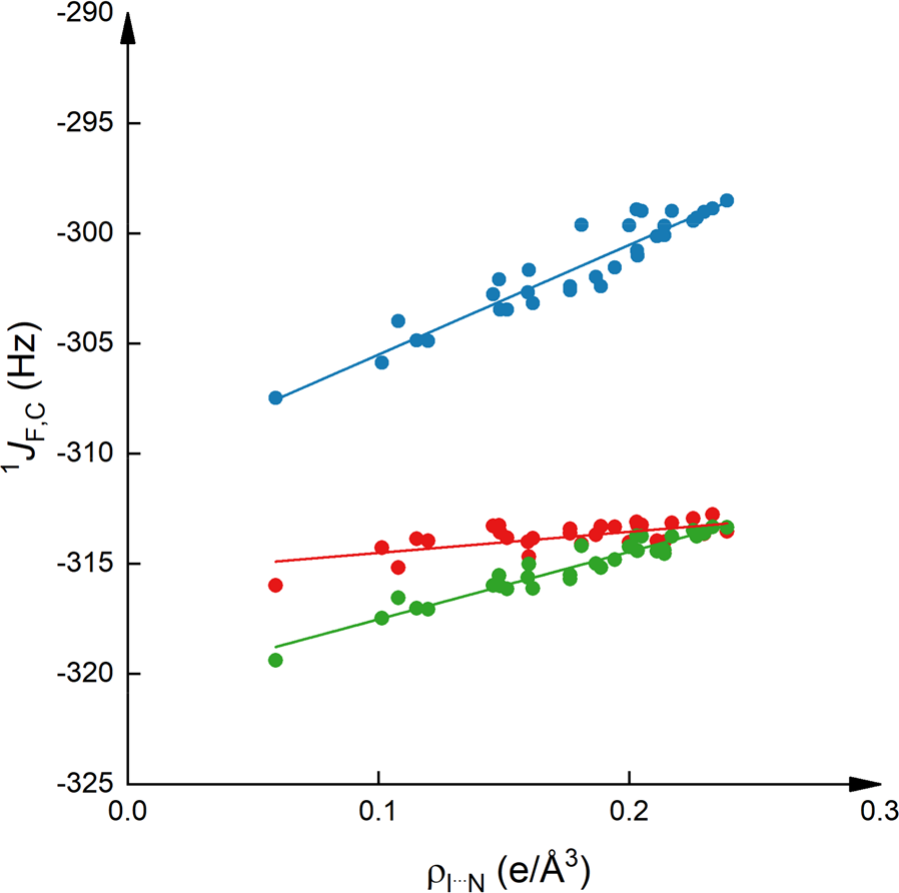
Computed ^1^*J*_F,C_ of 1-iodoperfluorooctane as a function of the electron density of the complexing Lewis base at the C–I···X bond critical point (ρ_I···N_). The data corresponding to the *ortho*-position are shown in blue (*R*^2^ = 0.89, slope 49.8), to the *meta* in red (*R*^2^ = 0.40, slope 9.6), and to the *para* in green (*R*^2^ = 0.92, slope 30.6).

We observed strong correlations between the ^1^*J*_F,C_ of 1-iodoperfluorooctane with the halogen bond binding energy, *E*_XB_ (*R*^2^ = 0.94), and with the electron density at the bond critical point, ρ_N···I_ (*R*^2^ = 0.95), upon complexation with pyridines (Figure 5 and Figures S21, Supporting Information). For 1-iodopentafluorobenzene analogous trends were observed for the correlation of the computed ^1^Δ*J*_F,C_ as a function of ρ_I···N_ (Figure S22, Supporting Information) as for the correlation of the experimental Δ^1^*J*_F,C_ as a function of p*K*_BI2_^2^ of the complexing Lewis base, namely, Δ^1^*J*_*o*__-F,C_ > Δ^1^*J*_*p*__-F,C_ > Δ^1^*J*_*m*__-F,C_ (Figure 2). The computed trends reproduce the experimental correlation of Δ^1^*J*_F,C_ to p*K*_BI2_^2^ (Figure 4) with the computed ^1^Δ*J*_F,C_ being larger at C_α_ as compared to C_β_ (Figure S24, Supporting Information).

A multicomponent analysis of the contributions of the Fermi contact (FC), the spin dipolar (SD), the paramagnetic spin–orbit (PSO), and the diamagnetic spin–orbit (DSO) components to the magnitude of the coupling constant, that is, ^1^*J*_F,C_ = ^1^*J*_FC_ + ^1^*J*_SD_ + ^1^*J*_PSO_ + ^1^*J*_DSO_, was performed (Tables S18–S20 and S40–S42, Supporting Information). Fermi contact contributions were shown to be important for the Δ^1^*J* upon hydrogen bonding of unrelated systems.^32^ The spin–orbit components are expected to be relevant for heavier halogens.^33^ Our analysis indicates that for the Δ^1^*J*s of 1-iodopentafluorobenzene, the spin–orbit contribution is increasingly relevant for C–F bonds more distant from the halogen bond donor (Table S15, Supporting Information). The Fermi contact contribution is dominant for the Δ^1^*J*_*o*-F,C_. Correlations in Δ^1^*J*_*m*-F,C_ suffer from large *R*^2^s. The Fermi contact and the paramagnetic spin–orbit terms are equally important for the Δ^1^*J*_*m*-F,C_ and Δ^1^*J*_*p*-F,C_ (Table S15, Figures S15–S17, Supporting Information). The spin–orbit contribution is larger for the *J*s in *m*- and *p*-positions as compared to the *o*-positions. Following the change in the natural occupations^34^ of 2s and 2p orbitals of carbon atoms, the complexation-induced change in ^1^*J*_F,C_ is dominated by the induction (2s, 2p_σ_) and resonance (2p_π_) for *o*- and *m*-positions, whereas by resonance (2p_π_) for the *p*-position (Tables S22–S24, Figures S10–S12, Supporting Information).

For the aliphatic 1-iodoperfluorooctane, the overall Δ^1^*J*_F,C_s are similar at the α- and β-positions. While the spin–orbit contributions are dominant for the Δ^1^*J*_α-F,C_s, the Fermi contact contributions are responsible for the overall Δ^1^*J*_β-F,C_s. It should be noted that a previous study reported the sensitivity of the ^1^*J*_C,H_ to the formation of strong C–H···X hydrogen bonds, with the dominant contribution to the magnitude change being the decrease of the Fermi contact term or an increase in the s-character of carbon hybridization for weaker complexes.^35^ The complexation-induced change in the coupling constants is dominated by induction (2s, 2p_σ_) effects, as revealed by NBO analysis (Tables S30 and S31, Figures S13 and S14, Supporting Information).

## Conclusions

In summary, scalar coupling constants are demonstrated to reflect the strength of halogen bonds in solution. The binding-specific changes are observable several bonds away from the binding site, also for rather weak interactions, in the solution phase. In contrast to detecting interactions by chemical shift changes, referencing is not needed for the observation of Δ*J*s, which improves accuracy. The halogen bond-induced Δ*J* predominantly originates from the Fermi contact and paramagnetic spin–orbit terms, whereas the spin dipolar contribution plays a smaller role and the diamagnetic spin–orbit contribution is negligible.

There is no indication that the weak interaction-induced alteration of distant coupling constants would depend on the type of coupling or the type of interaction. Accordingly, similar trends are expected to be detectable using ^1^*J*_H,C_, as demonstrated here using ^1^*J*_F,C_. Scalar couplings are expected not just to become an addition to the toolbox of techniques for the experimental characterization of halogen bonding^36^ but to be also applicable for the assessment of other types of weak σ-hole interactions,^37^ such as tetrel, pnictogen, chalcogen, and hydrogen bonds.

## Supporting Information Available

The Supporting Information is available free of charge at https://pubs.acs.org/doi/10.1021/jacs.1c04477. Original NMR FIDs are available, free of charge open access, at Zenodo with DOI: 10.5281/zenodo.4698893.

Experimental details, extracted data sets, computational details, and extracted data (PDF)
