# Supplementary material for: Probing Halogen Bonds by Scalar Couplings
Source: J Am Chem Soc. 2021 Jul 8;143(28):10695–9. doi: 10.1021/jacs.1c04477 (PMC8397312; doi:10.1021/jacs.1c04477)
Supplement: Supplementary file 1 — ja1c04477_si_001.pdf [file ja1c04477_si_001.pdf]

Electronic Supporting Information

Probing Halogen Bonds by Scalar Couplings

Bono Jimmink, Daniel Sethio, Lotta Turunen, Daniel von der Heiden, Máté Erdélyi*

Table of Contents

General information **S2**

The iodine basicity scale **S2**

1 Experimental Setup and Details **S2**

1.1. The Spectrometer settings relevant for the measurement accuracy of $^{1}J_{F,C}$s S2

1.2. Sample preparation S2

1.2.1. Experiment repetition S2

1.2.2. 1-Iodoperfluorobenzene samples S2

1.2.3. 1-Iodoperfluorooctane samples S7

1.3. Error estimation S8

1.3.1. The method used for error estimation in this study S8

1.3.2. Curve fitting algorithms S8

1.3.3. Comparison of the observed errors for the $\Delta^{1}J_{F,C}$ estimations for 1-iodopentafluorobenzene and 1-iodoheptadecafluorooctane S8

1.4. The influence of moisture S11

2 Experimental Data **S12**

2.1. 1-Iodoperfluorobenzene: a typical example S13

2.2. 1-Iodoperfluorobenzene: Base induced changes in the ^{19}F NMR S16

2.2.1. The $\Delta^{1}J_{F,C}$ and $\Delta\delta$ values for 1-iodopentafluorobenzene induced by Lewis bases S17

2.2.2. Subset analysis of $\Delta^{1}J_{ortho\text{-}F,C}$ values for 1-iodopentafluorobenzene induced by Lewis bases S18

2.3. 1-Iodoheptadecafluorooctane: A typical example S19

2.4. 1-Iodoperfluorooctane: Base induced changes in the ^{19}F NMR S21

2.4.1. The $\Delta^{1}J_{F,C}$ and $\Delta\delta$ values for 1-Iodoheptadecafluorooctane induced by Lewis bases S22

2.5. Evaluating the impact of Non-Directional Effects S22

3 Computational Details **S22**

4 Computational Results **S24**

4.1. Decomposition of the computed scalar couplings into FC, SD, PSO and DSO terms S39

5 Optimized Structures Obtained at the B3LYP-D3/aug-cc-pVTZ-pp Level of Theory **S46**

6 References **S77**

General information

The solvent CD_2Cl_2 was used as delivered (Eurisotop® lot: T1071) or was distilled over CaH_2; CH_2Cl_2 (VWR, AnalaR NORMAPUR Reag. Ph.Eur., ACS, water < 0.01 %, Lot 20G034019) was used as delivered. Dry solvents were stored over 3 Å molecular sieves in a glovebox. All other chemicals were purchased from commercial suppliers and used without further purification. For all synthesis and analytical studies, the glassware had been dried *in vacuo*, at least overnight. NMR spectra were recorded on an Agilent MR4000-DD2 equipped with a OneNMR probe. Chemical shifts are reported on the δ scale (ppm), with respect to the solvent deuterium lock signal. To assign the ^{19}F NMR resonances, chemical shifts (δ), multiplicity, coupling constants (J Hz) and integrals were considered. Multiplicities are denoted as s (singlet), d (doublet), t (triplet), q (quartet), hep (heptet), and m (multiplet). MestReNova 12.0.3. was used to process the NMR spectra.

The iodine basicity scale

The iodine basicity (e.g. pK_bI_2 and $\Delta\nu$(I-I) scale) gives Lewis base-type dependent correlations to observables that describe the non-covalent binding of Lewis bases to diiodine. NMR-spectroscopy detects a time-averaged signal of the species involved into the binding equilibrium. The pK_bI_2 scale describes the same equilibrium process. The iodine basicity observed for a halogen bond acceptor is interconvertible via a linear correlation justifying the $pK_bI_2{}^2$ dependency of the correlations described in this work.[1] There are a variety of basicity scales available, with the book "Lewis Basicity and Affinity Scales" by C. Laurence and J-F. Gal[2] providing a helpful overview of the scope of each.

1 Experimental Setup and Details

1.1. The Spectrometer settings relevant for the measurement accuracy of $^1J_{F,C}$s

^{19}F NMR spectra have been recorded for CD_2Cl_2 solutions at 25.0 °C, acquiring 128 scans with 1 sec relaxation delay at 376.25 MHz using specifications given in Table S1.

Table S2.

Table S1. Experimental parameters for ^{19}F measurements of 1-Iodoperfluorobenzene samples.

Spectral Width	30487.8 Hz
Acquired /Processed Size	131072 / 262144 points
Detected / Processed Digital Resolution	0.232 Hz /0.116 Hz

Table S2. Experimental parameters for ^{19}F measurements of 1-Iodoperfluorooctane samples.

Spectral Width	39062.5 Hz
Acquired / Processed Size	131072 / 262144 points
Detected / Processed Digital Resolution	0.298 Hz / 0.149 Hz

1.2. Sample preparation

1.2.1. Experiment repetition

Upon repetition of an experiment (^{19}F NMR measurement) a fresh sample was used. Each individual sample was measured fresh and prepared on a different day.

1.2.2. 1-Iodoperfluorobenzene samples

Each sample was prepared individually by mixing 1-Iodopentafluorobenzene (58.8±1.7% mg, 1.00 eq.) and 2.50 eq. equivalent of a base filled up to 1.00±0.01 mL (CD_2Cl_2:CH_2Cl_2 1:1 *v/v*) resulting in a set of consistent concentrations for a given halogen bond donor.

The filename format of the NMR rawdata, which is provided at the open access respository Zenodo with DOI: 10.5281/zenodo. 4698893: [YYYYMMDD]_[Operatorlabel]_[Systematiclabel]_[nonessentialdescriptions]

Table 3: Weigh-ins of experiments of 1-iodopentafluorobenzene and base with deviations.

Halogen bond donor···Base	Systematic labels:	Calcd. weigh-in base [mg]	Exp. weigh-in base [mg]	Dev.	Calcd. weigh-in C_6F_5I [mg]	Exp. weigh-in C_6F_5I [mg]	Dev.
Pentafluoroiodobenzene··· 4-Dimethylaminopyridine	ArI-3	0.0611	0.0614	0.5%	0.0588	0.0586	0.3%
Pentafluoroiodobenzene··· 4-Dimethylaminopyridine	ArI-9	0.0611	0.0609	0.3%	0.0588	0.0594	1.0%
Pentafluoroiodobenzene··· 4-Dimethylaminopyridine	ArI-15	0.0611	0.0611	0.0%	0.0588	0.0593	0.9%
Pentafluoroiodobenzene··· Pyridine	ArI-4	0.0396	0.0391	1.1%	0.0588	0.0593	0.9%
Pentafluoroiodobenzene··· Pyridine	ArI-10	0.0396	0.0398	0.6%	0.0588	0.0588	0.0%
Pentafluoroiodobenzene··· Pyridine	ArI-29	0.0396	0.0394	0.4%	0.0588	0.0592	0.7%
Pentafluoroiodobenzene··· Pyridine-N-oxide	ArI-5	0.0476	0.0474	0.3%	0.0588	0.0591	0.5%
Pentafluoroiodobenzene··· Pyridine-N-oxide	ArI-11	0.0476	0.0476	0.1%	0.0588	0.059	0.3%
Pentafluoroiodobenzene··· Pyridine-N-oxide	ArI-17	0.0476	0.0476	0.1%	0.0588	0.0596	1.4%
Pentafluoroiodobenzene··· 4-Methoxypyridine	ArI-6	0.0546	0.0544	0.3%	0.0588	0.0591	0.5%
Pentafluoroiodobenzene··· 4-Methoxypyridine	ArI-12	0.0546	0.0546	0.1%	0.0588	0.0586	0.3%
Pentafluoroiodobenzene··· 4-Methoxypyridine	ArI-18	0.0546	0.0546	0.1%	0.0588	0.0586	0.3%
Pentafluoroiodobenzene··· Trimethylamine-N-oxide	ArI-7	0.0376	0.0372	0.9%	0.0588	0.0589	0.2%
Pentafluoroiodobenzene··· Trimethylamine-N-oxide	ArI-13	0.0376	0.0378	0.7%	0.0588	0.0584	0.7%
Pentafluoroiodobenzene··· Trimethylamine-N-oxide	ArI-19	0.0376	0.0375	0.1%	0.0588	0.0596	1.4%
Pentafluoroiodobenzene··· N,N-Dimethyl-2-imidazolidinone	ArI-8	0.0571	0.0572	0.2%	0.0588	0.0592	0.7%
Pentafluoroiodobenzene··· N,N-Dimethyl-2-imidazolidinone	ArI-14	0.0571	0.0576	0.9%	0.0588	0.0586	0.3%
Pentafluoroiodobenzene··· N,N-Dimethyl-2-imidazolidinone	ArI-20	0.0571	0.0576	0.9%	0.0588	0.0598	1.7%
Pentafluoroiodobenzene··· Diethylamine	ArI-22	0.0366	0.0363	0.7%	0.0588	0.0591	0.5%
Pentafluoroiodobenzene··· Diethylamine	ArI-30	0.0366	0.0363	0.7%	0.0588	0.0582	1.0%
Pentafluoroiodobenzene··· Diethylamine	ArI-41	0.0366	0.037	1.2%	0.0588	0.0592	0.7%
Pentafluoroiodobenzene··· Butylamine	ArI-23	0.0366	0.0364	0.5%	0.0588	0.0591	0.5%
Pentafluoroiodobenzene··· Butylamine	ArI-31	0.0366	0.0369	0.9%	0.0588	0.0583	0.9%
Pentafluoroiodobenzene··· Butylamine	ArI-42	0.0366	0.0363	0.7%	0.0588	0.0585	0.5%
Pentafluoroiodobenzene··· Triphenylamine	ArI-24	0.1227	0.1222	0.4%	0.0588	0.0583	0.9%
Pentafluoroiodobenzene··· Triphenylamine	ArI-32	0.1227	0.1226	0.0%	0.0588	0.0596	1.4%

Halogen bond donor···Base	Systematic labels:	Calcd. weigh-in base [mg]	Exp. weigh-in base [mg]	Dev.	Calcd. weigh-in C_6F_5I [mg]	Exp. weigh-in C_6F_5I [mg]	Dev.
Pentafluoroiodobenzene··· Triphenylamine	ArI-43	0.1227	0.1229	0.2%	0.0588	0.059	0.3%
Pentafluoroiodobenzene··· Triphenylphosphineoxide	ArI-25	0.1437	0.1395	2.9%	0.0588	0.0588	0.0%
Pentafluoroiodobenzene··· Triphenylphosphineoxide	ArI-37	0.1437	0.1389	3.4%	0.0588	0.0586	0.3%
Pentafluoroiodobenzene··· Triphenylphosphineoxide	ArI-48	0.1437	0.1391	3.2%	0.0588	0.0594	1.0%
Pentafluoroiodobenzene··· Piperidine	ArI-26	0.0426	0.0424	0.4%	0.0588	0.0583	0.9%
Pentafluoroiodobenzene··· Piperidine	ArI-33	0.0426	0.0425	0.2%	0.0588	0.0583	0.9%
Pentafluoroiodobenzene··· Piperidine	ArI-44	0.0426	0.043	1.0%	0.0588	0.0594	1.0%
Pentafluoroiodobenzene··· 3-Chloropyridine	ArI-27	0.0568	0.0565	0.5%	0.0588	0.0594	1.0%
Pentafluoroiodobenzene··· 3-Chloropyridine	ArI-34	0.0568	0.0566	0.3%	0.0588	0.0589	0.2%
Pentafluoroiodobenzene··· 3-Chloropyridine	ArI-45	0.0568	0.0565	0.5%	0.0588	0.0586	0.3%
Pentafluoroiodobenzene··· Diisopropylamine	ArI-28	0.0646	0.0642	0.7%	0.0588	0.0587	0.2%
Pentafluoroiodobenzene··· Diisopropylamine	ArI-35	0.0646	0.0645	0.2%	0.0588	0.0585	0.5%
Pentafluoroiodobenzene··· Diisopropylamine	ArI-46	0.0646	0.0646	0.0%	0.0588	0.0589	0.2%
Pentafluoroiodobenzene··· Triethylamine	ArI-74	0.0506	0.0503	0.6%	0.0588	0.0592	0.7%
Pentafluoroiodobenzene··· Triethylamine	ArI-47	0.0506	0.0506	0.0%	0.0588	0.0589	0.2%
Pentafluoroiodobenzene··· Triethylamine	ArI-50	0.0506	0.0507	0.2%	0.0588	0.0588	0.0%
Pentafluoroiodobenzene··· Pyrrolidinone	ArI-39	0.0426	0.0427	0.3%	0.0588	0.0584	0.7%
Pentafluoroiodobenzene··· Pyrrolidinone	ArI-49	0.0426	0.0423	0.6%	0.0588	0.0591	0.5%
Pentafluoroiodobenzene··· Pyrrolidinone	ArI-51	0.0426	0.0426	0.1%	0.0588	0.059	0.3%
Pentafluoroiodobenzene··· 3-Bromopyridine	ArI-52	0.0790	0.0791	0.1%	0.0588	0.0587	0.2%
Pentafluoroiodobenzene··· 3-Bromopyridine	ArI-62	0.0790	0.0787	0.4%	0.0588	0.0588	0.0%
Pentafluoroiodobenzene··· 3-Bromopyridine	ArI-75	0.0790	0.0788	0.3%	0.0588	0.0589	0.2%
Pentafluoroiodobenzene··· 3,5-Dimethylpyridine	ArI-55	0.0536	0.0532	0.7%	0.0588	0.0583	0.9%
Pentafluoroiodobenzene··· 3,5-Dimethylpyridine	ArI-63	0.0536	0.0534	0.3%	0.0588	0.0589	0.2%
Pentafluoroiodobenzene··· 3,5-Dimethylpyridine	ArI-76	0.0536	0.0534	0.3%	0.0588	0.0589	0.2%
Pentafluoroiodobenzene··· 3,5-Dimethylpyridine	ArI-56	0.0536	0.0537	0.2%	0.0588	0.0594	1.0%
Pentafluoroiodobenzene··· Dimethylamine	ArI-64	0.0536	0.0532	0.7%	0.0588	0.0592	0.7%
Pentafluoroiodobenzene··· Dimethylamine	ArI-77	0.0536	0.0534	0.3%	0.0588	0.0589	0.2%
Pentafluoroiodobenzene··· 3,5-Dichloropyridine	ArI-57	0.0740	0.0741	0.1%	0.0588	0.0594	1.0%
Pentafluoroiodobenzene··· 3,5-Dichloropyridine	ArI-65	0.0740	0.0744	0.5%	0.0588	0.0593	0.9%

Halogen bond donor···Base	Systematic labels:	Calcd. weigh-in base [mg]	Exp. weigh-in base [mg]	Dev.	Calcd. weigh-in C_6F_5I [mg]	Exp. weigh-in C_6F_5I [mg]	Dev.
Pentafluoroiodobenzene··· 3,5-Dichloropyridine	ArI-78	0.0740	0.074	0.0%	0.0588	0.0594	1.0%
Pentafluoroiodobenzene··· sec-Butylamine	ArI-58	0.0366	0.0369	0.9%	0.0588	0.0585	0.5%
Pentafluoroiodobenzene··· sec-Butylamine	ArI-66	0.0366	0.0364	0.5%	0.0588	0.0589	0.2%
Pentafluoroiodobenzene··· sec-Butylamine	ArI-79	0.0366	0.0366	0.1%	0.0588	0.0591	0.5%
Pentafluoroiodobenzene··· Pyrrolidinone	ArI-59	0.0356	0.0354	0.5%	0.0588	0.0594	1.0%
Pentafluoroiodobenzene··· Pyrrolidine	ArI-91	0.0356	0.0356	0.1%	0.0588	0.0588	0.0%
Pentafluoroiodobenzene··· Pyrrolidine	ArI-80	0.0356	0.0354	0.5%	0.0588	0.0594	1.0%
Pentafluoroiodobenzene··· Diisopropylamine	ArI-60	0.0506	0.0503	0.6%	0.0588	0.0596	1.4%
Pentafluoroiodobenzene··· Diisopropylamine	ArI-68	0.0506	0.0506	0.0%	0.0588	0.0587	0.2%
Pentafluoroiodobenzene··· Diisopropylamine	ArI-81	0.0506	0.0504	0.4%	0.0588	0.0585	0.5%
Pentafluoroiodobenzene··· Ethylisopropylamine	ArI-61	0.0436	0.0439	0.7%	0.0588	0.0584	0.7%
Pentafluoroiodobenzene··· Ethylisopropylamine	ArI-69	0.0436	0.0432	0.9%	0.0588	0.0591	0.5%
Pentafluoroiodobenzene··· Ethylisopropylamine	ArI-82	0.0436	0.0435	0.2%	0.0588	0.0588	0.0%
Pentafluoroiodobenzene··· *tert*-Butylamine	ArI-70	0.0366	0.0366	0.1%	0.0588	0.0593	0.9%
Pentafluoroiodobenzene··· *tert*-Butylamine	ArI-83	0.0366	0.0362	1.0%	0.0588	0.0589	0.2%
Pentafluoroiodobenzene··· *tert*-Butylamine	ArI-86	0.0366	0.0362	1.0%	0.0588	0.0589	0.2%
Pentafluoroiodobenzene··· Dibutylamine	ArI-71	0.0646	0.0643	0.5%	0.0588	0.0591	0.5%
Pentafluoroiodobenzene··· Dibutylamine	ArI-84	0.0646	0.0644	0.3%	0.0588	0.0591	0.5%
Pentafluoroiodobenzene··· Dibutylamine	ArI-87	0.0646	0.0648	0.3%	0.0588	0.0584	0.7%
Pentafluoroiodobenzene··· Tributylamine	ArI-73	0.0927	0.0924	0.3%	0.0588	0.059	0.3%
Pentafluoroiodobenzene··· Tributylamine	ArI-85	0.0927	0.0925	0.2%	0.0588	0.0588	0.0%
Pentafluoroiodobenzene··· Tributylamine	ArI-88	0.0927	0.0928	0.1%	0.0588	0.0593	0.9%
Pentafluoroiodobenzene··· Diisopropylamine	ArI-89	0.0506	0.0505	0.2%	0.0588	0.0596	1.4%
Pentafluoroiodobenzene··· Diisopropylamine	ArI-92	0.0506	0.0502	0.8%	0.0588	0.0585	0.5%
Pentafluoroiodobenzene··· Diisopropylamine	ArI-103	0.0506	0.051	0.8%	0.0588	0.0585	0.5%
Pentafluoroiodobenzene··· 2-Methylpyridine	ArI-90	0.0466	0.0466	0.1%	0.0588	0.0583	0.9%
Pentafluoroiodobenzene··· 2-Methylpyridine	ArI-93	0.0466	0.0467	0.3%	0.0588	0.0583	0.9%
Pentafluoroiodobenzene··· Dimetylsulfoxide	ArI-94	0.0391	0.0394	0.8%	0.0588	0.059	0.3%
Pentafluoroiodobenzene··· Dimetylsulfoxide	ArI-106	0.0391	0.0386	1.2%	0.0588	0.0588	0.0%
Pentafluoroiodobenzene··· Dimetylsulfoxide	ArI-110	0.0391	0.0388	0.7%	0.0588	0.0582	1.0%

Halogen bond donor···Base	Systematic labels:	Calcd. weigh-in base [mg]	Exp. weigh-in base [mg]	Dev.	Calcd. weigh-in C_6F_5I [mg]	Exp. weigh-in C_6F_5I [mg]	Dev.
Pentafluoroiodobenzene··· Dimetylformamide	ArI-95	0.0365	0.0361	1.2%	0.0588	0.0592	0.7%
Pentafluoroiodobenzene··· Dimetylformamide	ArI-105	0.0365	0.0361	1.2%	0.0588	0.0595	1.2%
Pentafluoroiodobenzene··· Dimetylformamide	ArI-111	0.0365	0.0365	0.1%	0.0588	0.0588	0.0%
Pentafluoroiodobenzene··· Acetophenone	ArI-96	0.0601	0.06	0.1%	0.0588	0.0587	0.2%
Pentafluoroiodobenzene··· Acetophenone	ArI-101	0.0601	0.0602	0.2%	0.0588	0.059	0.3%
Pentafluoroiodobenzene··· Acetophenone	ArI-107	0.0601	0.06	0.1%	0.0588	0.0585	0.5%
Pentafluoroiodobenzene··· Benzophenone	ArI-97	0.0911	0.0906	0.6%	0.0588	0.0583	0.9%
Pentafluoroiodobenzene··· Benzophenone	ArI-108	0.0911	0.0898	1.4%	0.0588	0.0593	0.9%
Pentafluoroiodobenzene··· Benzophenone	ArI-112	0.0911	0.0911	0.0%	0.0588	0.0584	0.7%
Pentafluoroiodobenzene··· 4-Methylpyridine-N-oxide	ArI-98	0.0546	0.0548	0.4%	0.0588	0.0585	0.5%
Pentafluoroiodobenzene··· 4-Methylpyridine-N-oxide	ArI-109	0.0546	0.0543	0.5%	0.0588	0.0583	0.9%
Pentafluoroiodobenzene··· 4-Methylpyridine-N-oxide	ArI-113	0.0546	0.0548	0.4%	0.0588	0.0595	1.2%
	Max deviation			**3.4%**			**1.7%**
	Average weigh-in deviation			**0.5%**			**0.6%**

Control experiments

Pentafluoroiodobenzene··· n-pentane	DAN-ArI-9	0.0361	0.0364 = 57.8 μL	1.0 %	0.0588	0.0593	0.9%
Pentafluoroiodobenzene··· n-pentane	DAN-ArI-10	0.0361	0.0364 = 57.8 μL	1.0 %	0.0588	0.0595	1.2%
Pentafluoroiodobenzene··· n-pentane	DAN-ArI-11	0.0361	0.0364 = 57.8 μL	1.0 %	0.0588	0.0587	0.2%

1.2.3. 1-Iodoperfluorooctane samples

Each sample was prepared individually by mixing 1-iodoheptadecafluorooctane (109.2mg±0.9% , 1.00 eq.) and 2.50eq.±0.9% of a base filled up to 1.00±0.01 mL (CD_2Cl_2:CH_2Cl_2 1:1 *v/v*) resulting in a set of consistent concentrations for a given halogen bond donor.

Table 4: Weigh-ins of experiments of 1-iodoperfluorooctane and base with deviations.

Halogen bond donor···Base	Rawdata filename format: [date]_[Operator]_[Label]_[add.descri.] Labels:	XBA Calcd. weigh-in [mg]	XBA Exp. weigh-in [mg]	XBA Dev.	XBD Calcd. weigh-in [mg]	XBD Exp. weigh-in [mg]	XBD Dev.
Heptadecafluoroiodooctane··· 4-Dimethylaminopyridine	AliI-30	0.0611	0.0609	0.3%	0.1092	0.1099	0.6%
Heptadecafluoroiodooctane··· 4-Dimethylaminopyridine	AliI-36	0.0611	0.0614	0.5%	0.1092	0.1102	0.9%
Heptadecafluoroiodooctane··· Pyridine	AliI-31	0.0396	0.0392	0.9%	0.1092	0.1097	0.5%
Heptadecafluoroiodooctane··· Pyridine	AliI-37	0.0396	0.0395	0.1%	0.1092	0.1097	0.5%
Heptadecafluoroiodooctane··· Pyridine-N-oxide	AliI-32	0.0476	0.0472	0.7%	0.1092	0.1092	0.0%
Heptadecafluoroiodooctane··· Pyridine-N-oxide	AliI-38	0.0476	0.0475	0.1%	0.1092	0.1091	0.1%
Heptadecafluoroiodooctane··· 4-Methoxypyridine	AliI-33	0.0546	0.0547	0.2%	0.1092	0.1093	0.1%
Heptadecafluoroiodooctane··· 4-Methoxypyridine	AliI-39	0.0546	0.0545	0.1%	0.1092	0.1091	0.1%
Heptadecafluoroiodooctane··· 4-Methoxypyridine	AliI-49	0.0546	0.0549	0.6%	0.1092	0.1091	0.1%
Heptadecafluoroiodooctane··· 4-Methoxypyridine	AliI-45	0.0546	0.0545	0.1%	0.1092	0.1092	0.0%
Heptadecafluoroiodooctane··· 3,5-Dimethylpyridine	AliI-42	0.0536	0.0536	0.0%	0.1092	0.1096	0.4%
Heptadecafluoroiodooctane··· 3,5-Dimethylpyridine	AliI-46	0.0536	0.0538	0.4%	0.1092	0.1098	0.5%
Heptadecafluoroiodooctane··· 2,6-Dimethylpyridine	AliI-43	0.0536	0.0534	0.3%	0.1092	0.1099	0.6%
Heptadecafluoroiodooctane··· 2,6-Dimethylpyridine	AliI-47	0.0536	0.0535	0.1%	0.1092	0.1095	0.3%
Heptadecafluoroiodooctane··· 3-Bromopyridine	AliI-44	0.0790	0.0788	0.3%	0.1092	0.1089	0.3%
Heptadecafluoroiodooctane··· 3-Bromopyridine	AliI-48	0.0790	0.0793	0.4%	0.1092	0.110	0.7%
Heptadecafluoroiodooctane··· 3-Bromopyridine	AliI-50	0.0790	0.0791	0.1%	0.1092	0.1091	0.1%
Heptadecafluoroiodooctane··· 3-Chloropyridine	AliI-51	0.0568	0.0565	0.5%	0.1092	0.110	0.7%
Heptadecafluoroiodooctane··· 3-Chloropyridine	AliI-55	0.0568	0.0567	0.1%	0.1092	0.1092	0.0%
Heptadecafluoroiodooctane··· 3,5-Dichloropyridine	AliI-54	0.0740	0.0735	0.7%	0.1092	0.1093	0.1%
Heptadecafluoroiodooctane··· 3,5-Dichloropyridine	AliI-56	0.0740	0.0741	0.1%	0.1092	0.1097	0.5%
Heptadecafluoroiodooctane··· 2-Methylpyridine	AliI-53	0.0466	0.0464	0.4%	0.1092	0.1094	0.2%
Heptadecafluoroiodooctane··· 2-Methylpyridine	AliI-57	0.0466	0.0464	0.4%	0.1092	0.1096	0.4%
	Max deviation			**0.9%**			**0.9%**
	Average deviation			**0.3%**			**0.3%**

**Control experiments**							
Heptadecafluoroiodooctane··· *n*-pentane	DAN-AliI-12	0.0361	0.0364 = 57.8 μL	1.0 %	0.1092	0.1098	0.5%
Heptadecafluoroiodooctane··· *n*-pentane	DAN-AliI-13	0.0361	0.0364 = 57.8 μL	1.0 %	0.1092	0.1089	0.3%
Heptadecafluoroiodooctane··· *n*-pentane	DAN-AliI-14	0.0361	0.0364 = 57.8 μL	1.0 %	0.1092	0.1086	0.5%

1.3. Error estimation

We carefully investigated the errors possibly caused by traces of water, solvent and differences between samples (repetition). The acquired digital resolution is 0.232-0.298 Hz, whereas the processed is 0.116-0.149 Hz (see Section 1.1). The reproducibility of the read-out coupling constants has been 0.01-0.10 Hz, with 17 out of 114 $^{1}J_{F,C}$ that exceed these limits (see section 2.2). The concentrations of the samples have errors < 1.1%, based on the weigh-ins (see section 1.2.2 and 1.2.3).

1.3.1. The method used for error estimation in this study

The reported error of the $^{1}J_{F,C}$ (see 1.2.1) is the averaged ($\bar{x}$) value, based on $\bar{x} = {(\sum_{i=1}^{n} x_n)}/{n}$ "AVERAGE", and its standard deviation σ as $\sigma = \sqrt[2]{\frac{\sum_{i=1}^{n}(x_i-\bar{x})^2}{n-1}}$ (STDEV)

This method consistently gives the largest σ within the dataset/sample size, compared to alternatives tested. Alternative methods for describing variations of a very small datasets do treat σ differently, e.g. giving the ± range of the averaged value ($\frac{Max(A)-Min(A)}{2}$) instead of accessing variance.

1.3.2. Curve fitting algorithms

To fit functions to the dataset, a given function has been minimized by reducing its overall R^2 value without applying weighing methods, using the software Origin 2018.

1.3.3. Comparison of the observed errors for the $\Delta^{1}J_{F,C}$ estimations for 1-iodopentafluorobenzene and 1-iodoheptadecafluorooctane

The pyridine data sets with 1-iodopentafluorobenzene and 1-iodoheptadecafluorooctane have been compared to estimate which of the two halogen bond donors gives more reliable $\Delta^{1}J$ values. Despite the closer proximity of the observed $\Delta^{1}J_{F,C}$ measured for 1-iodoheptadecafluorooctane higher relative errors have been observed. We therefore decided to conduce the further study with 1-iodopentafluorobenzene.

Table S5. Average of three measurements of the $^{1}J_{ortho\text{-}F,C}$ of 1-iodopentafluorobenzene. Standard deviation and error in percentage was taken from *Table S11*.

	$\Delta^{1}J_{ortho\text{-}F,C}$ (Hz)	STDEV	Error in percentage [0.%%]
Pyrrolidine	2.57	0.22	0.09
4-Dimethylaminopyridine	2.14	0.03	0.01
3,5-Dimethylpyridine	1.03	0.02	0.02
4-Methoxypyridine	1.02	0.01	0.01
2-Methylpyridine	0.78	0.00	0.00

Pyridine	0.70	0.03	0.04
2,6-Dimethylpyridine	0.34	0.02	0.06
3-Bromopyridine	0.28	0.01	0.04
3-Chloropyridine	0.30	0.02	0.07
3,5-Dichloropyridine	0.05	0.00	0.09
Average			**0.043**

Table S6. Average of two measurements of the $^1J_{\alpha\text{-F,C}}$ in 1-Iodoheptadecafluoroocatane. Standard deviation and error in percentage. Data have been taken from *Table S14*.

	$\Delta^1J_{\alpha\text{-F,C}}$ (Hz)	STDEV	Error in percentage [0.%%]
4-Dimethylaminopyridine	3.32	0.19	0.06
3,5-Dimethylpyridine	2.33	0.01	0.00
4-Methoxypyridine	2.08	0.15	0.07
2-Methylpyridine	2.00	0.12	0.06
Pyridine	1.65	0.09	0.06
2,6-Dimethylpyridine	1.03	0.02	0.02
3-Bromopyridine	1.35	0.31	0.23
3-Chloropyridine	1.82	0.09	0.05
3,5-Dichloropyridine	0.58	0.09	0.16
Average			**0.079**

1.4. The influence of moisture

To estimate the influence of moisture on the detected ${}^{1}J_{F,C}$, we recorded the ${}^{19}F$ NMR spectrum of 1-iodoheptadecafluorooctane in dry CD_2Cl_2 (Eurisotop® lot: T1071) and in mixtures of non-dried CD_2Cl_2 (Eurisotop® lot: T1071) and CH_2Cl_2 (VWR, Lot 20G034019).
The influence of water residues was insignificant (Table S1), with the variations between the samples being within the error limits. Averaging the coupling constants read on several individual peaks of a multiplet for a single coupling constant gives a lower error than the digital resolution of the spectra would permit.

Table S7. Comparison of the ${}^{1}J_{\alpha\text{-}F,C}$ determined for 1-Iodoheptadecafluorooctane using different solvent sources. Note that the calculated average value and the standard deviation are both below the digital resolution of the NMR data.

α-F, CD_2Cl_2 distilled from CaH_2 and stored over Molecular sieves

Peak	Hz	Peak	Hz	$\lvert{}^{1}J_{\alpha\text{-}F,C}\rvert$ (Hz)
1	-22460.30	4	-22780.79	320.49
2	-22475.35	5	-22795.88	320.53
3	-22490.28	6	-22810.97	320.69
			Average	320.57
			stdev	**0.11**
			digital resolution	**0.14**

α-F, 50% technical grade CH_2Cl_2 + 50% non-dried CD_2Cl_2

Peak	Hz	Peak	Hz	$\lvert{}^{1}J_{a\text{-}F,C}\rvert$ (Hz)
1	-22457.78	4	-22778.2	320.42
2	-22472.91	5	-22793.39	320.48
3	-22487.78	6	-22808.35	320.57
			Average	320.49
			stdev	**0.08**
			digital resolution	**0.15**

2 Experimental Data

Table S8. Halogen bond donors used in this study and the iodine basicity of the Lewis bases applied.

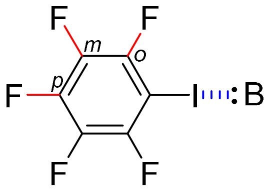

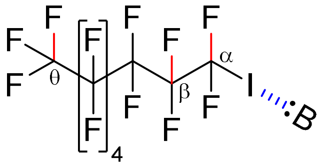

Pyridines	pK_{BI2}[1]		Amines	pK_{BI2}[1]	N-Oxides and Carbonyls	pK_{BI2}[1]
Pyrrolidine	3.85		Butylamine	3.00	DMSO	1.56
4-Dimethylaminopyridine	3.78	1°	sec-butylamine	2.78	1,3-dimethyl-2-imidazolidinone	1.22
3,5-Dimethylpyridine	2.78		tert-butylamine	2.86	2-Pyrrolidone	1.20
4-Methoxypyridine	2.63		Diethylamine	3.73	DMF	0.81
2-Methylpyridine	2.35		Dipropylamine	3.58	Acetophenone	0.06
Pyridine	2.22		Dibutylamine	3.58	Benzophenone	-0.07
2,6-Dimethylpyridine	1.83	2°	Ethylisopropylamine		Trimethylamin-N-Oxide	3.68
3-Bromopyridine	1.40		Diisopropylamine	2.85	Pyridine-N-Oxide	2.40
3-Chloropyridine	1.38		Piperidine	3.85	4-Methylpyridine-N-Oxide	2.31
3,5-Dichloropyridine	0.81		Triethylamine	3.67	Triphenylphosphine oxide	2.08
			Tributylamine	3.05		
		3°	Diisopropylethylamine	Not available		
			Triphenylamine	Not available		

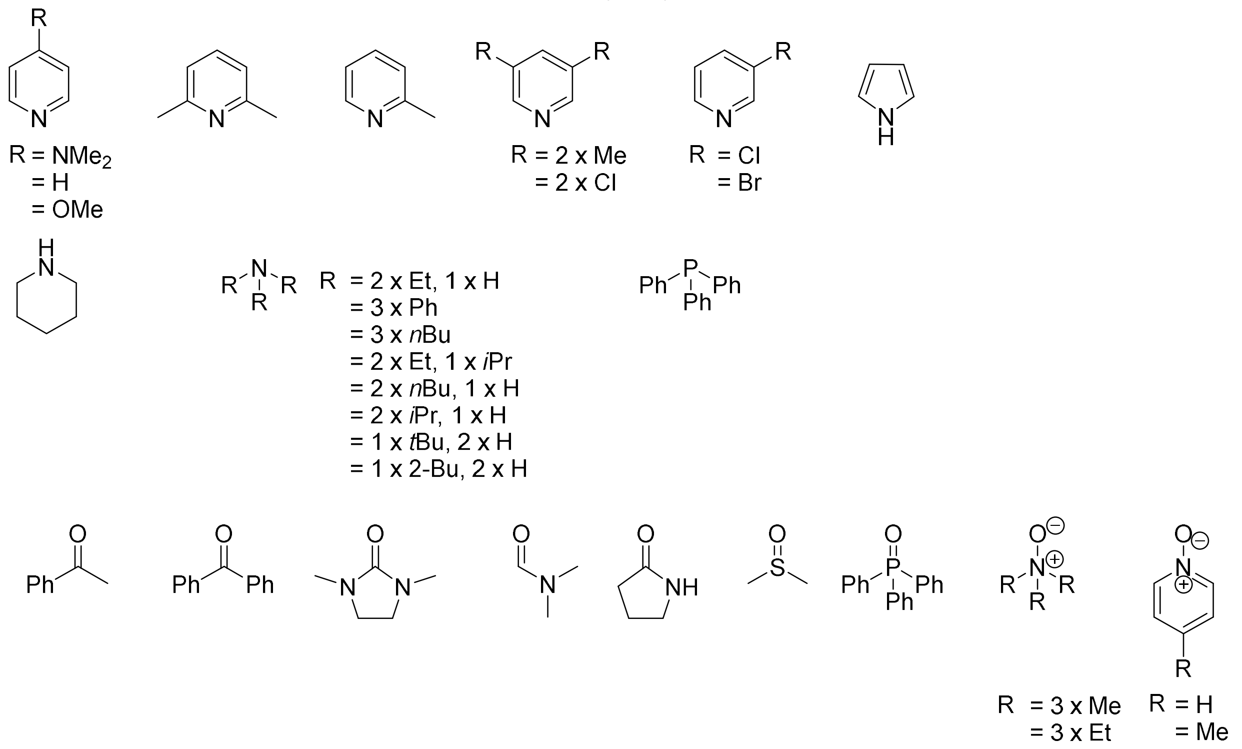

Figure S1. Summary of the Lewis bases evaluated for halogen bonding.

2.1. 1-Iodoperfluorobenzene: a typical example

Enlarged multiplets of the ^{19}F NMR spectrum of 1-iodopentafluorobenzene without and in the presence of 4-methoxypyridine. The multiplet patterns don't change in the presence of the base. The ^{19}F NMR spectra corresponds to the data given in Figure S2 and Figure S3.

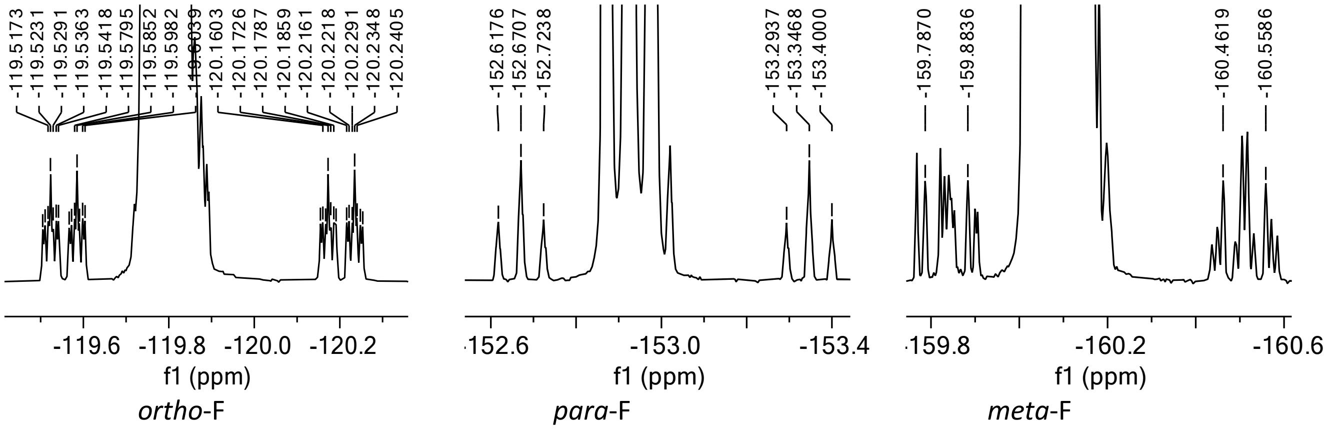

Figure S2. Enlarged multiplets of the ^{19}F NMR spectrum of 1-iodopentafluorobenzene without the presence of a Lewis base.

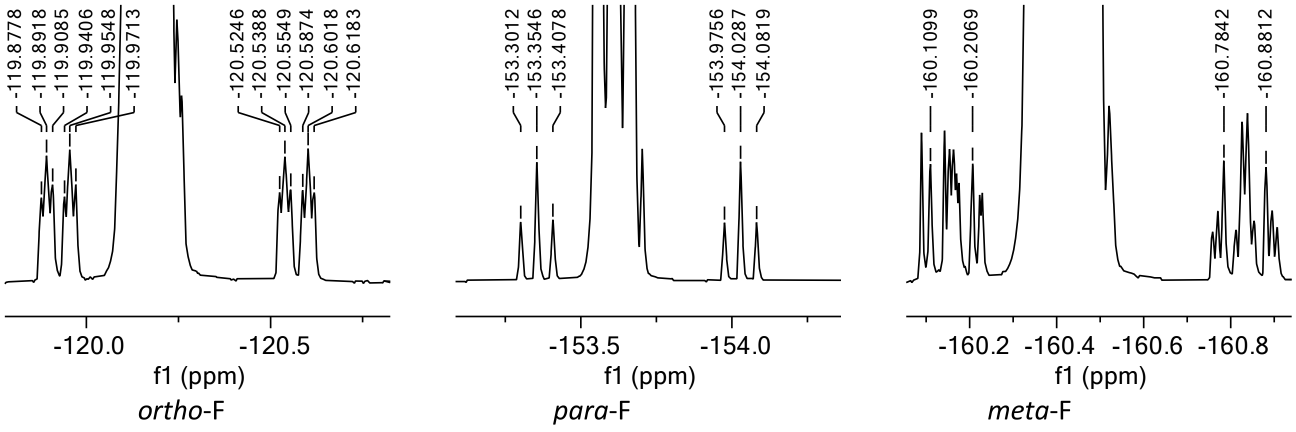

Figure S3. Enlarged multiplets of the ^{19}F NMR spectrum of 1-iodopentafluorobenzene in the presence of 4-methoxypyridine.

Table S9.Extracted peaks and calculated $^{1}J_{F,C}$ coupling constants in 1-Iodopentafluorobenzene without the presence of a base.

Multiplet information	Rawdata filename format: [date]_[Operator]_[Label]_[add.descri.] Labels:	Peak	Hz	Peak	Hz	$\|^{1}J_{F,C}\|$ (Hz)
$^{1}J_{ortho\text{-}F,C}$	ArI-Ref1	1	-44969.9	15	-45214.2	244.3
		2	-44971.8	16	-45216.2	244.4
		3	-44974.5	17	-45218.9	244.4
		4	-44976.7	18	-45221.1	244.4
		5	-44978.8	19	-45223.3	244.5
		6	-44981.5	20	-45226.1	244.6
		7	-44983.7	21	-45228.1	244.4
		8	-44993	22	-45237.4	244.4
		9	-44995.2	23	-45239.5	244.3
		10	-44997.9	24	-45242.3	244.4
		11	-44999.9	25	-45244.5	244.6
		12	-45002.1	26	-45246.5	244.4
		13	-45004.8	27	-45249.5	244.7
		14	-45007	28	-45251.5	244.5
$^{1}J_{para\text{-}F,C}$	ArI-Ref1	1	-57430.2	4	-57684.6	254.4
		2	-57450.2	5	-57704.6	254.4
		3	-57470.2	6	-57724.6	254.4
$^{1}J_{meta\text{-}F,C}$	ArI-Ref1	1	-60128	3	-60382	254.0
		2	-60164.4	4	-60418.4	254.0

		Peak	Hz	Peak	Hz	$\|^{1}J_{F,C}\|$ (Hz)
$^{1}J_{ortho\text{-}F,C}$	ArI-Ref2	1	-44970.1	15	-45214.7	244.6
		2	-44972.2	16	-45216.6	244.4
		3	-44974.9	17	-45219.2	244.3
		4	-44977.1	18	-45221.5	244.4
		5	-44979.1	19	-45223.7	244.6
		6	-44982.1	20	-45226.4	244.3
		7	-44984	21	-45228.4	244.4
		8	-44993.4	22	-45237.9	244.5
		9	-44995.5	23	-45239.9	244.4
		10	-44998.3	24	-45242.7	244.4
		11	-45000.3	25	-45244.8	244.5
		12	-45002.5	26	-45246.8	244.3
		13	-45005.3	27	-45249.9	244.6
		14	-45007.4	28	-45251.9	244.5
$^{1}J_{para\text{-}F,C}$	ArI-Ref2	1	-57430.2	4	-57684.6	254.4
		2	-57450.2	5	-57704.6	254.4
		3	-57470.2	6	-57724.6	254.4

$^{1}J_{meta\text{-}F,C}$	ArI-Ref2	1	-60128.8	3	-60382.9	254.1
		2	-60165.1	4	-60419.2	254.1

		Peak	Hz	Peak	Hz	$\lvert ^{1}J_{F,C} \rvert$ (Hz)
$^{1}J_{ortho\text{-}F,C}$	ArI-Ref5	1	-44970.1	15	-45214.7	244.6
		2	-44972.2	16	-45216.6	244.4
		3	-44974.9	17	-45219.2	244.3
		4	-44977.1	18	-45221.5	244.4
		5	-44979.1	19	-45223.7	244.6
		6	-44982.1	20	-45226.4	244.3
		7	-44984	21	-45228.4	244.4
		8	-44993.4	22	-45237.9	244.5
		9	-44995.5	23	-45239.9	244.4
		10	-44998.3	24	-45242.7	244.4
		11	-45000.3	25	-45244.8	244.5
		12	-45002.5	26	-45246.8	244.3
		13	-45005.3	27	-45249.9	244.6
		14	-45007.4	28	-45251.9	244.5
$^{1}J_{para\text{-}F,C}$	ArI-Ref5	1	-57430.2	4	-57684.6	254.4
		2	-57450.2	5	-57704.6	254.4
		3	-57470.2	6	-57724.6	254.4
$^{1}J_{meta\text{-}F,C}$	ArI-Ref5	1	-60128.8	3	-60382.9	254.1
		2	-60165.1	4	-60419.2	254.1

Table S10. Extracted peaks and calculated $^{1}J_{F,C}$ coupling constants in 1-iodopentafluorobenzene in the presence of 4-methoxypyridine.

Multiplet information	Rawdata filename format: [date]_[Operator]_[Label]_[add.descri.] Labels:	Peak	Hz	Peak	Hz	$\lvert ^{1}J_{F,C} \rvert$ (Hz)
$^{1}J_{ortho\text{-}F,C}$	ArI-6	1	-45110.2	7	-45353.6	243.4
		2	-45115.4	8	-45358.9	243.5
		3	-45121.7	9	-45364.9	243.2
		4	-45133.8	10	-45377.2	243.4
		5	-45139.1	11	-45382.6	243.5
		6	-45145.3	12	-45388.8	243.5
$^{1}J_{para\text{-}F,C}$	ArI-6	1	-57687.4	4	-57941.2	253.8
		2	-57707.5	5	-57961.2	253.7
		3	-57727.5	6	-57981.2	253.7
$^{1}J_{meta\text{-}F,C}$	ArI-6	1	-60249.5	3	-60503.3	253.8
		2	-60286	4	-60539.8	253.8

2.2. 1-Iodoperfluorobenzene: Base induced changes in the ^{19}F NMR

Table S11. The average changes in the ^{19}F-^{13}C-^{1}J coupling constants ($AVARAGE(\Delta\ ^{1}J_{^{13}C^{19}F})$), chemical shift ($AVARAGE(\Delta\delta\ ^{19}F_{^{13}C^{19}F})$) as well as their standard deviations (STDEV) of 1-iodoperfluorobenzene (c = 0.200 mol/L) by the presence of selected halogen bond acceptors (c = 0.500 mol/L). All samples have been prepared trice.

Compound	$AVARAGE(\Delta\ ^{1}J_{^{13}C_{orthoF}})$ $[Hz]$	STDEV	$AVARAGE(\Delta\ ^{1}J_{^{13}C_{paraF}})$ $[Hz]$	STDEV	$AVARAGE(\Delta\ ^{1}J_{^{13}C_{metaF}})$ $[Hz]$	STDEV	$AVARAGE(\Delta\delta\ \mathrm{ortho}^{19}F_{^{13}C^{19}F})$ $[ppm]$	STDEV	$AVARAGE(\Delta\delta\ \mathrm{para}^{19}F_{^{13}C^{19}F})$ $[ppm]$	STDEV	$AVARAGE(\Delta\delta\ \mathrm{meta}^{19}F_{^{13}C^{19}F})$ $[ppm]$	STDEV	pK_{bI_2}	$(pK_{bI_2})^2$
**Pyridines**														
4-Dimethylaminopyridine	2.14	0.03	1.48	0.02	0.52	0.03	0.686	0.007	1.284	0.014	0.595	0.006	3.78	14.29
3,5-Dimethylpyridine	1.03	0.02	0.28	0.85	0.27	0.03	0.367	0.005	0.726	0.010	0.346	0.007	2.78	7.73
Methoxypyridine	1.02	0.01	0.71	0.05	0.27	0.03	0.355	0.008	0.663	0.017	0.314	0.007	2.63	6.92
2-Methylpyridine	0.78	0.00	0.53	0.05	0.25	0.00	0.266	0.004	0.514	0.008	0.228	0.005	2.35	5.52
Pyridine	0.70	0.03	0.47	0.04	0.29	0.12	0.235	0.005	0.467	0.012	0.214	0.005	2.22	4.93
2,6-Dimethylpyridine	0.34	0.02	0.08	0.27	0.08	0.03	0.097	0.002	0.254	0.003	0.079	0.000	1.83	3.35
3-Bromopyridine	0.28	0.01	0.09	0.19	0.05	0.00	0.004	0.001	0.111	0.005	0.031	0.000	1.40	1.96
3-Chloropyridine	0.30	0.02	0.20	0.01	0.13	0.10	0.058	0.002	0.178	0.005	0.042	0.001	1.38	1.90
3,5-Dichloropyridine	0.05	0.00	0.00	0.05	0.05	0.09	0.061	0.001	0.005	0.001	0.071	0.000	0.81	0.66
**Primary Amines**														
Butylamine	1.34	0.07	0.87	0.03	0.32	0.08	0.674	0.006	1.003	0.009	0.547	0.006	3	0.07
Secbutylamine	1.06	0.02	0.24	0.82	0.23	0.08	0.533	0.003	0.795	0.004	0.434	0.004	2.78	0.02
Tertbutylamine	1.18	0.01	0.27	0.95	0.27	0.03	0.612	0.006	0.901	0.010	0.470	0.005	2.86	0.01
**Secondary Amines**														
Diethylamine	1.99	0.04	1.24	0.04	0.35	0.05	0.920	0.024	1.374	0.034	0.736	0.019	3.73	13.91
Dipropylamine	1.69	0.09	0.98	0.15	0.22	0.03	0.776	0.010	1.223	0.014	0.650	0.008	3.58	12.81
Diisopropylamine	0.73	0.03	0.18	0.47	0.02	0.02	0.368	0.003	0.628	0.007	0.320	0.003	3.58	12.81
N-ethylisopropylamine	1.23	0.03	0.29	1.00	0.22	0.06	0.66	0.02	1.01	0.02	0.53	0.01		
Piperidine	2.69	0.04	1.61	0.04	0.25	0.26	1.171	0.010	1.702	0.015	0.874	0.003	2.85	8.12
Pyrrolidine	2.57	0.22	0.62	1.84	0.45	0.05	1.140	0.103	1.702	0.118	0.880	0.064	3.85	14.82
**Tertiary Amines**														
Triethylamine	1.30	0.06	0.92	0.04	0.12	0.03	0.634	0.011	1.072	0.017	0.565	0.010	3.67	13.47
Tributylamine	0.50	0.00	0.11	0.33	0.08	0.12	0.228	0.007	0.571	0.014	0.277	0.006	3.05	9.30
**Carbonyls**														
DMSO	0.61	0.01	0.46	0.05	0.35	0.13	0.183	0.002	0.388	0.004	0.205	0.003	1.56	2.43
1,3-dimethyl-2-imidazolidinone	0.49	0.02	0.40	0.00	0.32	0.06	0.186	0.008	0.399	0.015	0.243	0.009	1.22	1.50
2-Pyrrolidon	0.38	0.01	0.24	0.02	0.15	0.00	0.150	0.004	0.279	0.006	0.170	0.003	1.20	1.44
Dimethylformamide	0.39	0.03	0.32	0.02	0.23	0.06	0.181	0.003	0.367	0.006	0.245	0.004	0.81	0.66
Acetophenone	0.07	0.02	0.07	0.03	0.07	0.08	0.046	0.000	0.019	0.001	0.076	0.000	0.06	0.00
Benzophenone	0.03	0.02	0.04	0.02	0.00	0.05	0.181	0.008	0.134	0.005	0.245	0.010	-0.07	0.00
**N-Oxides**														
Trimethylamin-N-Oxide	4.04	0.02	2.82	0.07	1.10	0.02	1.437	0.016	2.716	0.028	1.447	0.016	3.68	13.53

Pyridine-N-Oxide	1.09	0.01	0.79	0.08	0.38	0.06	0.293	0.008	0.686	0.019	0.390	0.067	2.40	5.76
4-Methylpyridine-N-Oxide	1.49	0.04	1.06	0.05	0.52	0.06	0.425	0.011	0.991	0.021	0.511	0.009	2.31	5.35
Triphenylphosphine oxide	0.71	0.02	0.53	0.03	0.20	0.00	0.153	0.004	0.263	0.003	0.077	0.004	2.08	4.33
**Control without basic additives (not part of the graphes**														
n-Pentane	0.06	0.04	0.13	0.14	0.07	0.17	0.040	0.000	1.703	0.001	0.074	0.001	-	-

2.2.1. The $\Delta^1J_{F,C}$ and $\Delta\delta$ values for 1-iodopentafluorobenzene induced by Lewis bases

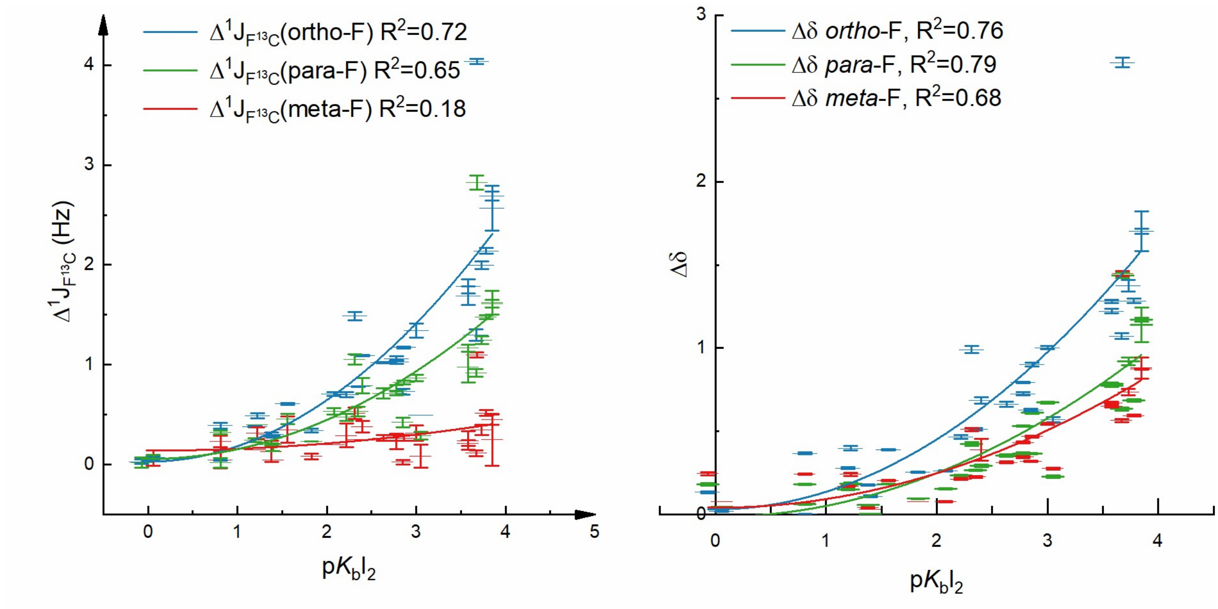

Figure S4: The $\Delta^1J_{F,C}$ and $\Delta\delta$ values for 1-iodopentafluorobenzene induced by the presence of a base, based on Table S11.

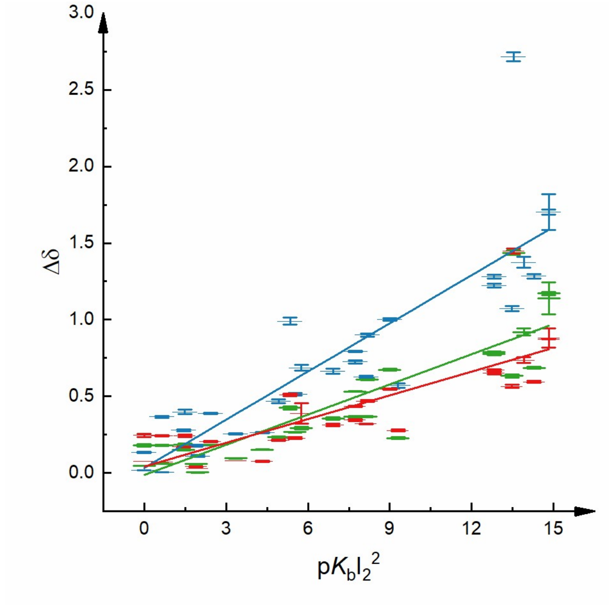

Figure S5: The $\Delta\delta_{ortho}$ (blue, R^2 = 0.79, slope 0.066), $\Delta\delta_{meta}$ (red, R^2 = 0.68, slope 0.052), $\Delta\delta_{para}$ (green, R^2 = 0.76, slope 0.10) values vs pK_{BI2}^2 for 1-iodopentafluorobenzene induced by the presence of a base, based on Table S11.

2.2.2. Subset analysis of $\Delta^1 J_{ortho\text{-F,C}}$ values for 1-iodopentafluorobenzene induced by Lewis bases

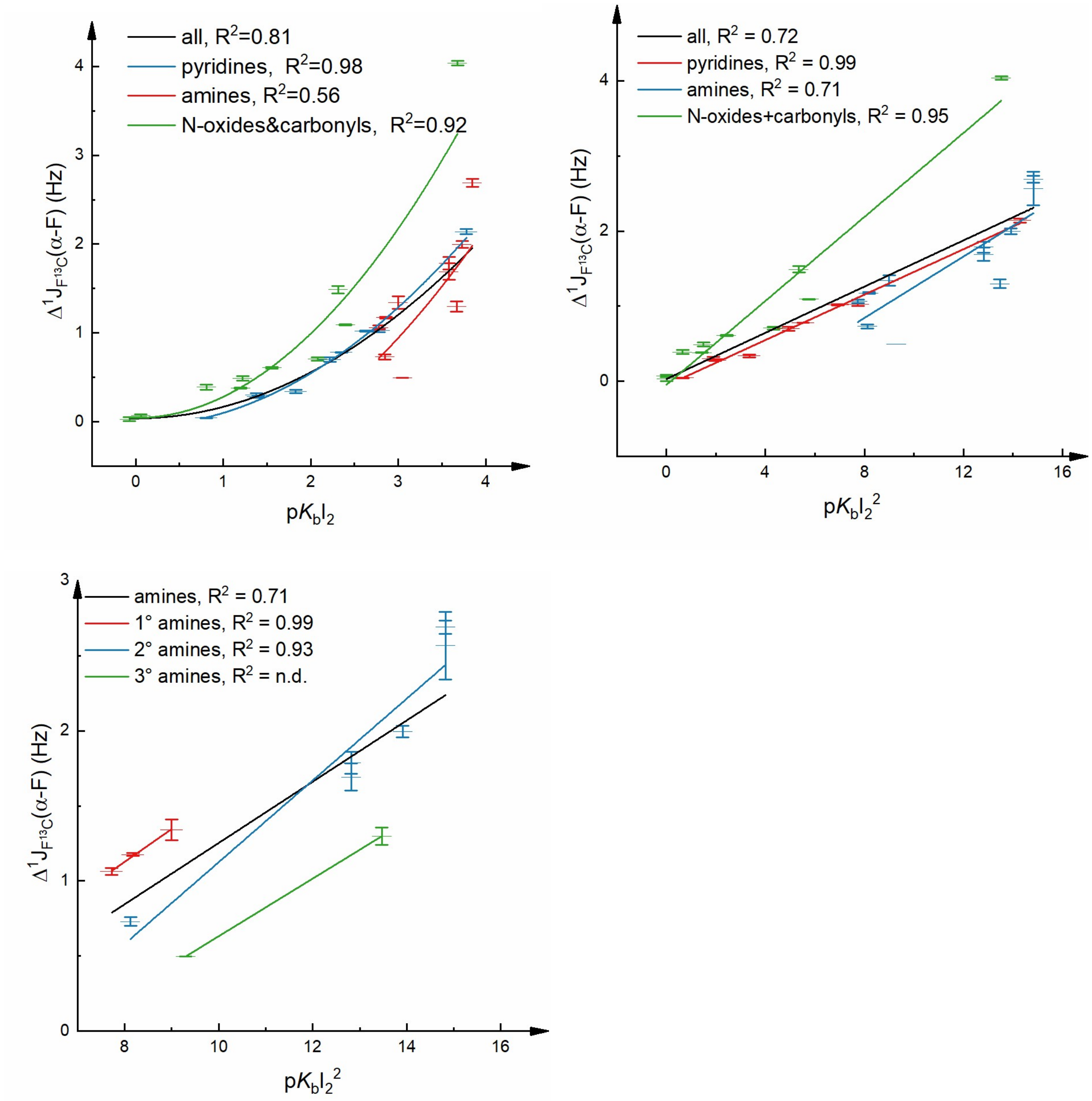

Figure S6: The $\Delta^1 J_{F,C}$ and $\Delta\delta$ values for 1-iodopentafluorobenzene induced by Lewis bases, based on Table S11. Top-left: $\Delta^1 J_{ortho\text{-}F,13C}$ vs pK_{BI2} grouped according to the type of base employed. The data points have been fitted to ax^2+b. top-right: The change of $^1J_{F,C}$ in the ortho-position of iodopentafluorobenzene, $\Delta^1 J_{ortho\text{-}F,C}$, as a function of the Lewis basicity, pK_{BI2}^2, upon binding to various Lewis bases. Errors are given as standard deviations; $pK_{BI2} = 0$ refers to a K = 1. The data corresponding to the pyridines is shown in red ($R^2 = 0.98$), to amines in blue ($R^2 = 0.60$), to N-oxides in green ($R^2 = 0.94$), whereas to all data in black ($R^2 = 0.71$).
Bottom-left: $\Delta^1 J_{ortho\text{-}F,13C}$ vs pK_{BI2}^2 of the amine subset grouped into 1°,2° and 3° with their linear correlation.

2.3. 1-Iodoheptadecafluorooctane: A typical example

Expansions of the ^{19}F NMR spectrum of 1-iodoheptadecafluorooctane without and in the presence of 4-methoxypyridine. The multiplet patterns don't change upon addition of a Lewis base. The ^{19}F NMR spectra correspond to the data given in Figure S7 and Figure S8.

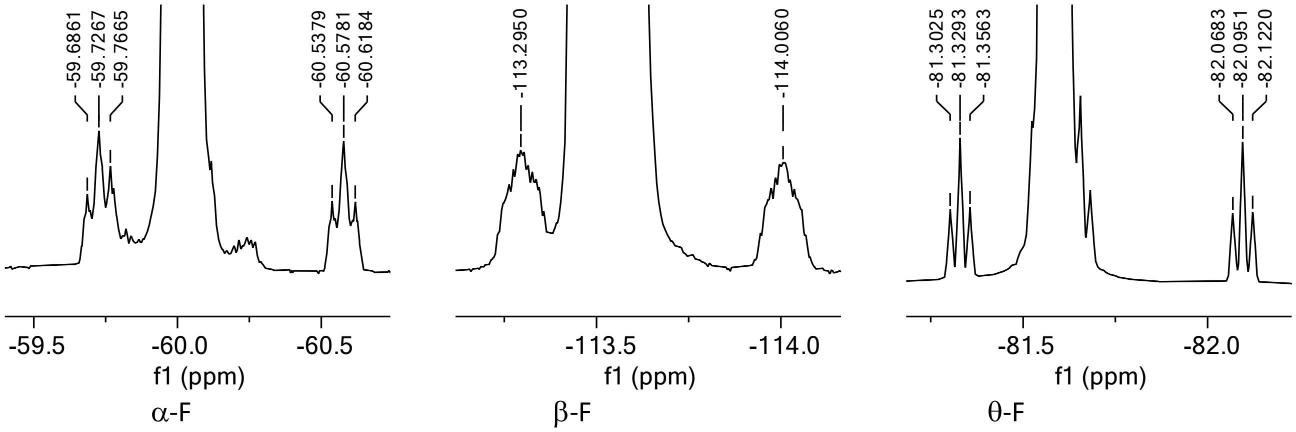

Figure S7. Cut-outs of the ^{19}F NMR spectrum of 1-Iodoheptadecafluorooctane without the presence of a base (AliI_Ref7) showing satellites with peaks picked.

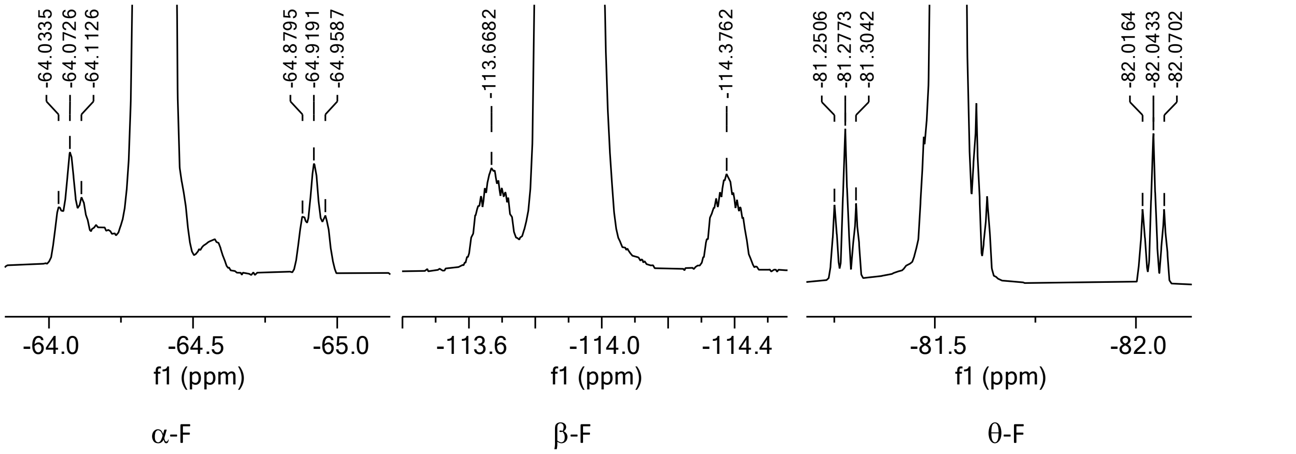

Figure S8. Cut-outs of the ^{19}F NMR spectrum of 1-iodoheptadecafluorooctane in the presence of 4-methoxypyridine showing satellites with peaks picked.

Table S12. Extracted peaks and calculated $^1J_{F,C}$ coupling constants in 1-iodopentafluorobenzene without the presence of a base.

Multiplet information	Rawdata filename format: [date]_[Operator]_[Label]_[add.descri.] Labels:	Peak	Hz	Peak	Hz	$\|^1J_{F,C}\|$ (Hz)
$^1J_{\alpha\text{-}F,C}$	ArI-Ref7	1	-22459.9	4	-22780.5	320.6
		2	-22475.2	5	-22795.6	320.4
		3	-22490.2	6	-22810.8	320.6
$^1J_{\beta\text{-}F,C}$	ArI-Ref7	1	-42633	2	-42900.6	267.6
$^1J_{\theta\text{-}F,C}$	ArI-Ref7	1	-30594.2	4	-30882.4	288.2
		2	-30604.3	5	-30892.5	288.2
		3	-30614.5	6	-30902.6	288.1

		Peak	Hz	Peak	Hz	$\|^1J_{F,C}\|$ (Hz)
$^1J_{\alpha\text{-}F,C}$	ArI-Ref8	1	-22459.8	4	-22780.4	320.6
		2	-22475.2	5	-22795.6	320.4
		3	-22490	6	-22810.7	320.7
$^1J_{\beta\text{-}F,C}$	ArI-Ref8	1	-42632	2	-42900.3	268.3
$^1J_{\theta\text{-}F,C}$	ArI-Ref8	1	-30592.4	4	-30880.5	288.1
		2	-30602.5	5	-30890.6	288.1
		3	-30612.6	6	-30900.8	288.2

Table S13. Extracted peaks and calculated $^1J_{F,C}$ coupling constants in 1-iodopentafluorobenzene in the presence of 4-methoxypyridine.

Multiplet information	Rawdata filename format: [date]_[Operator]_[Label]_[add.descri.] Labels:	Peak	Hz	Peak	Hz	$^1J_{a\text{-}F,C}$ (Hz)
$^1J_{\alpha\text{-}F,C}$	ArI-45	1	-24095.9	4	-24414.2	318.3
		2	-24110.6	5	-24429.1	318.5
		3	-24125.6	6	-24444	318.4
$^1J_{\beta\text{-}F,C}$	ArI-45	1	-42773.5	2	-43039.9	266.4
$^1J_{\theta\text{-}F,C}$	ArI-45	1	-30580.2	4	-30868.4	288.2
		2	-30590.3	5	-30878.5	288.2
		3	-30600.5	6	-30888.6	288.1

2.4. 1-Iodoperfluorooctane: Base induced changes in the ^{19}F NMR

Changes in selected chemical shifts and coupling constants of 1-iodoperfluorooctane are given in Table S14. The changes in coupling constant observed for the terminal CF_3 group are within the error of the experiment. Significant chemical shift changes of the terminal CF_3 group has been rationalized by Ciancaleoni et. al. as secondary interaction with the aromatic π-system leading to an orientation of the chain above the aromatic cycle and therefore remote Fluorine resonances experience a shielding effect.[3]

Table S14. The average changes in the ^{19}F-^{13}C-^{1}J coupling constants ($AVARAGE(\Delta\ ^{1}J_{^{13}C^{19}F})$), chemical shift ($AVARAGE(\Delta\delta\ ^{19}F_{^{13}C^{19}F})$) as well as their standard deviations (STDEV) of 1-Iodoperfluorooctane (c = 0.200 mol/L) by the presence of selected halogen bond acceptors (c = 0.500 mol/L). Samples have been prepared at least twice.

Compound	$AVARAGE(\Delta\ ^{1}J_{^{13}C\alpha F})$ $[Hz]$	STDEV	$AVARAGE(\Delta\ ^{1}J_{^{13}C\beta F})$ $[Hz]$	STDEV	$AVARAGE(\Delta\ ^{1}J_{^{13}C\theta F})$ $[Hz]$	STDEV	$AVARAGE(\Delta\delta\ \alpha^{19}F_{^{13}C^{19}F})$ $[ppm]$	STDEV	$AVARAGE(\Delta\delta\ \beta^{19}F_{^{13}C^{19}F})$ $[ppm]$	STDEV	$AVARAGE(\Delta\delta\ \theta^{19}F_{^{13}C^{19}F})$ $[ppm]$	STDEV	pK_{bI_2}	$(pK_{bI_2})^2$
**Pyridines**														
4-Dimethylaminopyridine	3.32	0.19	2.75	0.28	0.07	0.05	8.47	0.11	0.71	0.01	0.13	0.01	3.78	14.29
3,5-Dimethylpyridine	2.33	0.01	1.70	0.07	0.05	0.05	4.71	0.03	0.39	0.00	0.07	0.00	2.78	7.73
Methoxypyridine[a]	2.08	0.15	1.98	0.30	0.05	0.05	4.40	0.11	0.38	0.01	0.04	0.01	2.63	6.92
Pyridine-*N*-oxide	1.65	0.09	1.25	0.57	0.05	0.02	3.24	0.09	0.26	0.01	0.05	0.02	2.40	5.76
Pyridine	1.03	0.02	1.05	0.14	0.02	0.04	1.84	0.04	0.12	0.12	0.08	0.04	2.22	4.93
2,6-Dimethylpyridine	1.35	0.31	0.62	0.21	0.02	0.05	1.55	0.01	0.10	0.01	0.05	0.01	1.83	3.35
3-Bromopyridine[b]	1.82	0.09	0.60	0.07	0.05	0.00	1.52	0.02	0.10	0.00	0.04	0.00	1.40	1.96
3-Chloropyridine	2.28	0.00	1.45	0.14	0.00	0.05	3.98	0.04	0.37	0.01	0.00	0.02	1.38	1.90
Control without basic additives (not part of the graphes														
n-Pentane	0.42	0.11	0.10	0.04	2.15	0.30	0.036	0.002	0.173	0.002	0.070	0.001	-	-

[a]*sample has been prepared four-times,* [b]*sample has been prepared three times.*

2.4.1. The $\Delta^1J_{F,C}$ and $\Delta\delta$ values for 1-Iodoheptadecafluorooctane induced by Lewis bases

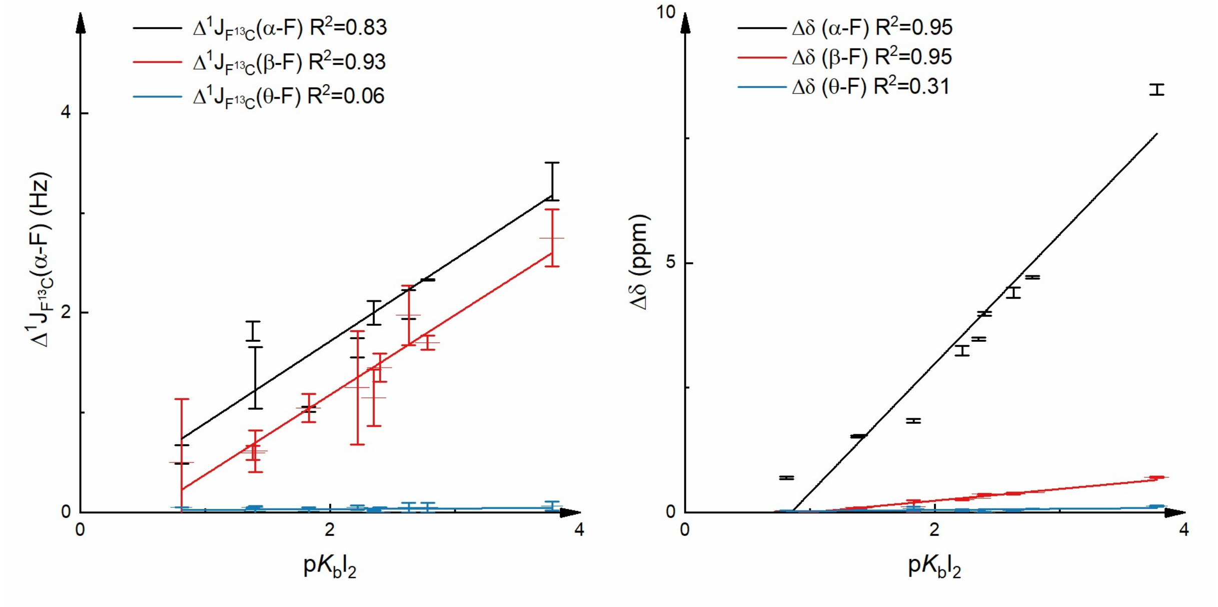

Figure S9. Left: Δ^1J_{F13C} in iodoperfluorooctane (c = 0.200 mol×L^{-1}) vs the iodine basicity (pK_bI_2) of a present base (c = 0.500 mol×L^{-1}). α-F (black), β-F (red) and θ-F (blue). Right: $\Delta\delta F$ in iodoperfluorooctane (c = 0.200 mol×L^{-1}) vs the iodine basicity (pK_{BI2}) of a present base (c = 0.500 mol×L^{-1}). α-F (black), β-F (red) and θ-F (blue).

2.5. Evaluating the impact of Non-Directional Effects

Addition of Lewis bases does not affect the $^1J_{F,C}$ of the terminal θ-CF_3 group of IC_8F_{17} whereas it influences in $^1J_{F,C}$ $^1J_{\alpha F,C}$ and $^1J_{\beta F,C}$ in a Lewis basicity dependent manner (Figure 4, main text). Significant change, 2.15 Hz, was observed for the $^1J_{F,C}$ of the θ-CF_3 group upon addition of 2.5 eq. pentane to the solution of the halogen bond donor, whereas the $^1J_{F,C}$ of the α- and β-CF_2 groups were not affected by *n*-pentane significantly. This observation indicates that the remote θ-CF_3 group serves as an internal reference to detect non-directional solvent effects introduced by changes in the bulk properties of the solution e.g. polarity. Thus, halogen bonding is observable close to the halogen bond donor site at the α- and β-CF_2 groups but not at the remote θ-CF_3 group whereas bulk solvent effects are detectable on the θ-CF_3 but not on the α- and β-CF_2 groups.

Iodopentafluorobenzene lacks a comparable reference position. However, upon addition of 2.5 eq. *n*-pentane only minor, $\Delta^1J_{F,C} < 0.07$ Hz, were observed at the *ortho* and *para*- $^1J_{F,C}$ whereas that at the the *meta* position has been slightly more sensitive ($\Delta^1J_{F,C} = 0.13$ Hz). Halogen bonding shows the opposite effect (larger change at the *ortho* and *para*- $^1J_{F,C}$) whereas no significant Lewis basicity dependent change at the *meta* position.

Overall, no significant influence of solvent polarity has been observed upon *n*-pentane addition, suggesting that the Lewis bases used in this study did not induce changes in $^1J_{F,C}$ by changing the solvents bulk properties.

3 Computational Details

All geometry optimization calculations for all halogen bonded complexes investigated in this work were carried out utilizing the B3LYP[4-5] functional augmented with Grimme's D3[6] dispersion correction in combination with the large correlation consistent Dunning's aug-cc-pVTZ[7-8] basis set. Scalar relativistic effects for heavy atoms (e.g. I) were assessed by utilizing the Stuttgart-Dresden (SDD)[9-10] effective core potential. The B3LYP-D3 functional was chosen as it is known to adequately account for electron correlations for

systems exhibiting noncovalent interactions.[11-12] Dichloromethane solvation effects were included using the polarizable continuum model (PCM) of Tomasi and co-workers.[13] Vibrational frequency calculations were followed at the same level of theory to confirm the optimized geometry corresponding to geometry minima.

All calculations were performed using the Gaussian 16 Rev. C.01 package.[14] The geometries were optimized using an ultrafine grid and tight convergence criteria for the forces and displacements.[15] Natural population analysis and second-order perturbation of the Fock matrix analysis of two interacting orbitals were carried out utilizing the NBO7 program.[16] Topological analysis of electron density were carried out using the AIMALL version 19.10.12 program.[17] The nature of halogen bonding interactions were characterized through the energy density (H_c) at the N···I and O···I bond critical points, where a negative value of the energy ($H_c < 0$) indicates covalent bond and a positive value of the energy ($H_c > 0$) points to electrostatic interactions.[18]

The associated binding energies (ΔE) were calculated by taking the energy difference between halogen bonded complexes (A···B) and its isolated components (A and B) at their equilibrium geometries,

$$A + B \rightarrow A\cdots B$$

$$\Delta E = E_{A\cdots B} - (E_A + E_B).$$

Due to the size of the molecules, the coupling constant calculations of 1-iodoperluorooctane were replaced with 1-iodoperfluoropropane.

4 Computational Results

Table S15. Contributions of the Fermi-contact (F,C), the spin dipolar (SD), the paramagnetic (PSO), and the diamagnetic spin-orbit (DSO) components to the change of $^1J_{F,C}$ ($\Delta^1J_{F,C}$) of the halogen bond donor upon halogen bonding. The slope of the computed contribution of the component as a function of $\Delta^1J_{F,C}$ are given, with the variance of the linear fits being shown in brackets. The first three rows show data computed for 1-iodopentafluorobenzene (IC_6F_5), whereas the next two data for 1-iodoperfluorooctane (IC_8F_{17}). Visualisation of the data used for extraction of these values can be found in section 4.1.

	FC	SD	PSO	DSO
$\Delta^1J_{o\text{-}F,C}$	0.713	0.076	0.209	0.000
IC_6F_5	(0.99)	(0.81)	(0.93)	(0.00)
$\Delta^1J_{m\text{-}F,C}$	0.435	0.150	0.416	0.002
IC_6F_5	(0.43)	(0.55)	(0.57)	(0.31)
$\Delta^1J_{p\text{-}F,C}$	0.459	0.126	0.414	0.000
IC_6F_5	(0.96)	(0.97)	(0.97)	(0.00)
$\Delta^1J_{\alpha\text{-}F,C}$	0.076	0.207	0.720	-0.003
IC_8F_{17}	(0.18 [a])	(0.97)	(0.97)	(0.89)
$\Delta^1J_{\beta\text{-}F,C}$	0.615	0.098	0.281	-0.020
IC_8F_{17}	(0.98)	(0.95)	(0.97)	(0.63)

[a] The weak correlation is caused by a single outlier.

Table S16. Bond distances (R), ^{19}F-^{13}C-^{1}J-coupling constants ($J_{F,C}$), binding energies (ΔE), electron densities (ρ) and energy densities (H) at the C-F and N···I bond critical point, calculated at the B3LYP-D3/aug-cc-pVTZ-pp

C_6F_5-I···Base	R_{C-F} (Å)	R_{C-I} (Å)	$R_{N\cdots I}$ (Å)	$^1J_{F,C}$ (Hz)	ΔE (kJ/mol)	ρ_{C-F} (e/Å^3)	H_{C-F} (h/Å^3)	$\rho_{N\cdots I}$ (e/Å^3)	$H_{N\cdots I}$ (h/Å^3)
–	1.337	2.099	-	-312.60	-	1.83824	-2.7164	-	-
4-Dimethylaminopyridine	1.343	2.148	2.723	-299.43	-31.86	1.80666	-2.6495	0.22552	-0.0114
2-Methylpyridine	1.342	2.135	2.819	-301.99	-29.02	1.81308	-2.6631	0.18682	-0.0013
2,6-Dimethylpyridine	1.341	2.131	2.904	-302.69	-28.86	1.81521	-2.6677	0.15958	+0.0035
4-Methoxypyridine	1.342	2.139	2.773	-300.79	-28.53	1.81099	-2.6586	0.20322	-0.0049
3,5-Dimethylpyridine	1.342	2.139	2.775	-301.02	-28.44	1.81113	-2.6589	0.20344	-0.0052
Pyridine	1.342	2.136	2.796	-301.55	-26.99	1.81293	-2.6628	0.19420	-0.0026
3-Bromopyridine	1.341	2.129	2.840	-302.49	-24.51	1.81653	-2.6704	0.17639	+0.0014
3-Chloropyridine	1.341	2.129	2.840	-302.42	-24.39	1.81641	-2.6701	0.17636	+0.0014
3,5-Dichloropyridine	1.340	2.124	2.880	-303.18	-22.40	1.81936	-2.6764	0.16166	+0.0042

Table S17. Hybridization characters and natural population charge analysis (X) of C, F, N, and I atoms, calculated at the B3LYP-D3/aug-cc-pVTZ-pp

C_6F_5-I···Base	sp^n C_{ortho}	sp^n F_{ortho}	sp^n C	sp^{n*} I	X_{C_ipso} (in e)	X_I (in e)	$X_{F(ortho)}$ (in e)	X_N (in e)
–	$sp^{3.19}$	$sp^{2.49}$	$sp^{2.96}$	$sp^{10.24}$	-0.2931	+0.2517	-0.3075	-
4-Dimethylaminopyridine	$sp^{3.26}$	$sp^{2.49}$	$sp^{2.78}$	$sp^{17.74}$	-0.3197	+0.2591	-0.3183	-0.5211
2-Methylpyridine	$sp^{3.25}$	$sp^{2.49}$	$sp^{2.79}$	$sp^{15.53}$	-0.3158	+0.2585	-0.3159	-0.4768
2,6-Dimethylpyridine	$sp^{3.24}$	$sp^{2.50}$	$sp^{2.80}$	$sp^{14.73}$	-0.3143	+0.2586	-0.3149	-0.4898
4-Methoxypyridine	$sp^{3.25}$	$sp^{2.49}$	$sp^{2.79}$	$sp^{16.27}$	-0.3172	+0.2601	-0.3168	-0.4953
3,5-Dimethylpyridine	$sp^{3.25}$	$sp^{2.49}$	$sp^{2.79}$	$sp^{16.22}$	-0.3171	+0.2593	-0.3167	-0.4577
Pyridine	$sp^{3.25}$	$sp^{2.49}$	$sp^{2.80}$	$sp^{15.71}$	-0.3159	+0.2599	-0.3161	-0.4662
3-Bromopyridine	$sp^{3.24}$	$sp^{2.49}$	$sp^{2.81}$	$sp^{14.69}$	-0.3139	+0.2605	-0.3148	-0.4454
3-Chloropyridine	$sp^{3.24}$	$sp^{2.49}$	$sp^{2.81}$	$sp^{14.69}$	-0.3139	+0.2606	-0.3149	-0.4458
3,5-Dichloropyridine	$sp^{3.23}$	$sp^{2.49}$	$sp^{2.82}$	$sp^{13.93}$	-0.3120	+0.2606	-0.3139	-0.4262

*Due to the use of effective core potential for describing relativistic effects of I atom

Table S18. Decomposition terms of calculated $^1J_{F,C}$ coupling constants into Fermi contact (FC), spin-dipolar (SD), paramagnetic spin-orbital (PSO), diamagnetic spin-orbit (DSO), and the total $^1J_{F,C}$ coupling constants at the ortho position as well as the second-order perturbation of the Fock Matrix between σ orbital of C-I and σ orbital of C-F, calculated at the B3LYP-D3/aug-cc-pVTZ-pp*

C_6F_5-I···Base	Fermi Contact (Hz)	Spin-Dipolar (Hz)	Paramagnetic Spin-orbit (Hz)	Diamagnetic Spin-orbit (Hz)	$^1J_{F,C}$(Hz)	C-I → C-F (kcal/mol)
–	-310.78	3.96	-6.94	1.17	-312.60	1.33
4-Dimethylaminopyridine	-300.76	4.82	-4.67	1.18	-299.43	1.68
2-Methylpyridine	-302.67	4.65	-5.15	1.18	-301.99	1.57
2,6-Dimethylpyridine	-303.11	4.58	-5.34	1.18	-302.69	1.52
4-Methoxypyridine	-301.69	4.17	-4.98	1.18	-300.79	1.61
3,5-Dimethylpyridine	-301.90	4.70	-5.00	1.18	-301.02	1.61
Pyridine	-302.26	4.65	-5.13	1.18	-301.55	1.58
3-Bromopyridine	-302.83	4.56	-5.39	1.18	-302.49	1.54
3-Chloropyridine	-302.78	4.56	-5.38	1.18	-302.42	1.53
3,5-Dichloropyridine	-303.24	4.48	-5.59	1.18	-303.18	1.50

Table S19. Decomposition terms of calculated $^1J_{F,C}$ coupling constants into Fermi contact (FC), spin-dipolar (SD), paramagnetic spin-orbital (PSO), diamagnetic spin-orbit (DSO), and the total $^1J_{F,C}$ coupling constants at the meta position (in Hz), calculated at the B3LYP-D3/aug-cc-pVTZ-pp

C_6F_5-I⋯Base	Fermi Contact	Spin-Dipolar	Paramagnetic Spin-orbit	Diamagnetic Spin-orbit	$^1J_{F,C}$(Hz)
–	-320.78	4.86	-2.50	1.15	-317.26
4-Dimethylaminopyridine	-319.13	5.57	-0.54	1.16	-312.94
2-Methylpyridine	-319.32	5.42	-0.96	1.16	-313.69
2,6-Dimethylpyridine	-319.50	5.38	-1.07	1.16	-314.02
4-Methoxypyridine	-318.96	5.47	-0.82	1.16	-313.15
3,5-Dimethylpyridine	-319.07	5.47	-0.83	1.16	-313.27
Pyridine	-318.97	5.43	-0.95	1.16	-313.33
3-Bromopyridine	-318.96	5.34	-1.17	1.16	-313.63
3-Chloropyridine	-318.94	5.35	-1.16	1.16	-313.60
3,5-Dichloropyridine	-318.94	5.28	-1.36	1.16	-313.86

Table S20. Decomposition terms of calculated $^1J_{F,C}$ coupling constants into Fermi contact (FC), spin-dipolar (SD), paramagnetic spin-orbital (PSO), diamagnetic spin-orbit (DSO), and the total $^1J_{F,C}$ coupling constants at the para position (in Hz), calculated at the B3LYP-D3/aug-cc-pVTZ-pp

C_6F_5-I⋯Base	Fermi Contact	Spin-Dipolar	Paramagnetic Spin-orbit	Diamagnetic Spin-orbit	$^1J_{F,C}$(Hz)
–	-320.92	4.12	-5.45	1.15	-321.11
4-Dimethylaminopyridine	-317.03	4.97	-2.58	1.15	-313.49
2-Methylpyridine	-317.71	4.78	-3.23	1.15	-315.01
2,6-Dimethylpyridine	-318.12	4.73	-3.39	1.15	-315.63
4-Methoxypyridine	-317.38	4.84	-3.02	1.15	-314.41
3,5-Dimethylpyridine	-317.37	4.84	-3.02	1.15	-314.41
Pyridine	-317.55	4.78	-3.21	1.15	-314.83
3-Bromopyridine	-317.79	4.68	-3.55	1.15	-315.51
3-Chloropyridine	-317.80	4.68	-3.54	1.15	-315.51
3,5-Dichloropyridine	-318.05	4.60	-3.82	1.15	-316.13

Table S21. Calculated ^{19}F coordination shifts ($\Delta\delta = \delta_{F(complex)} - \delta_{F(free)}$) in ppm

C_6F_5-I⋯Base	$\Delta\delta_{ortho}$	$\Delta\delta_{meta}$	$\Delta\delta_{para}$
–	43.55	2.48	10.50
4-Dimethylaminopyridine	40.39	0.47	6.14
2-Methylpyridine	40.96	0.96	7.20
2,6-Dimethylpyridine	41.36	1.05	7.50
4-Methoxypyridine	40.74	0.76	6.83
3,5-Dimethylpyridine	40.83	0.84	6.86
Pyridine	40.92	0.92	7.14
3-Bromopyridine	41.20	1.15	7.68
3-Chloropyridine	41.21	1.19	7.67
3,5-Dichloropyridine	41.45	1.40	8.13

Table S22. Natural occupation numbers of 2s and 2p orbitals of C atoms in the ortho-position, calculated at the B3LYP-D3/aug-cc-pVTZ-pp.

C_6F_5-I···Base	2s ($2s_\sigma$)	2px ($2p_\sigma$)	2py ($2p_\sigma$)	2pz ($2p_\pi$)	$^1J_{F,C}$(Hz)	Δ(SUM(2σ))[a]	$\Delta(2\pi)$ [a]
–	0.8545	0.9671	0.7921	1.0322	-312.6	0.000 (reference)	0.000 (reference)
4-Dimethylaminopyridine	0.8582	0.9668	0.7919	1.0366	-299.43	0.00331	0.004
2-Methylpyridine	0.8574	0.9673	0.7911	1.0358	-301.99	0.00212	0.004
2,6-Dimethylpyridine	0.8572	0.9669	0.7919	1.0353	-302.69	0.00230	0.003
4-Methoxypyridine	0.8577	0.9786	0.7797	1.0362	-300.79	0.00229	0.004
3,5-Dimethylpyridine	0.8577	0.9668	0.7922	1.0354	-301.02	0.00305	0.003
Pyridine	0.8574	0.9669	0.7914	1.0359	-301.55	0.00205	0.004
3-Bromopyridine	0.8570	1.0253	0.7329	1.0355	-302.49	0.00148	0.003
3-Chloropyridine	0.8570	1.0045	0.7537	1.0355	-302.42	0.00146	0.003
3,5-Dichloropyridine	0.8566	0.9669	0.7913	1.0350	-303.18	0.00111	0.003

[a] *The aromatic rings were placed in the XY plane (in-plane), whereas the π orbitals are located in the out-of-plane (Z-axis). The σ orbitals are assumed to have sp_2-hybridization with equal contribution from 2s, 2px, and 2py.*

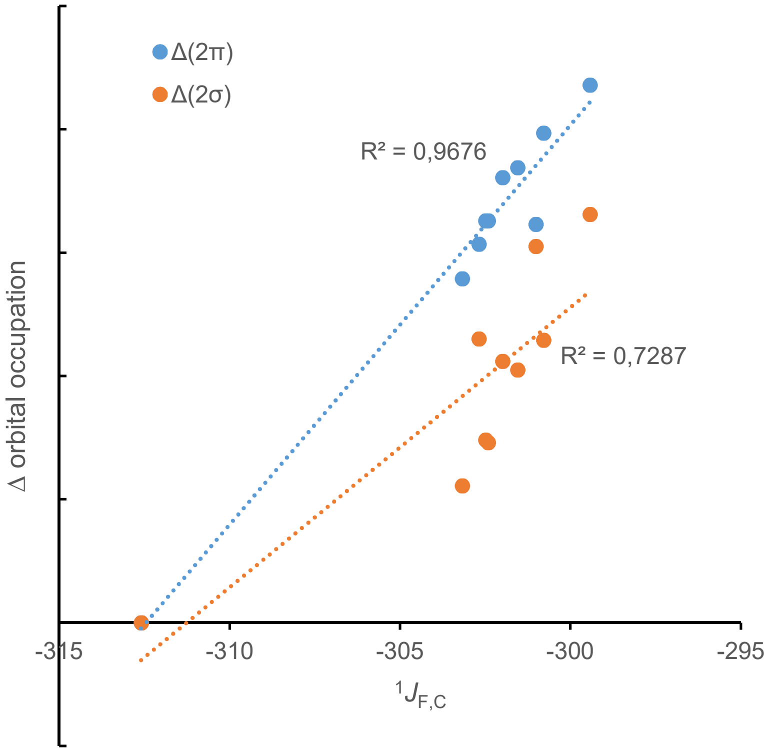

Figure S10: Trends between calculated $^1J_{F,C}$ coupling constants and natural occupation of 2s and 2p orbitals of the ortho-C-atom in iodopentafluorobenzene on halogen bond formation with different pyridine bases.

Table S23. Natural occupation numbers of 2s and 2p orbitals of C atoms in the meta-position, calculated at the B3LYP-D3/aug-cc-pVTZ-pp

C_6F_5-I···Base	2s	2px	2py	2pz	$^1J_{F,C}$(Hz)	Δ(SUM(2σ))[a]	Δ(2π)[a]
–	0.871	0.966	0.773	1.068	-312.6	0.000 (reference)	0.000 (reference)
4-Dimethylaminopyridine	0.874	0.969	0.770	1.072	-299.43	0.004	0.00219
2-Methylpyridine	0.873	0.968	0.771	1.072	-301.99	0.004	0.00138
2,6-Dimethylpyridine	0.873	0.968	0.771	1.071	-302.69	0.003	0.00143
4-Methoxypyridine	0.873	0.958	0.781	1.072	-300.79	0.004	0.00152
3,5-Dimethylpyridine	0.873	0.969	0.771	1.071	-301.02	0.003	0.00241
Pyridine	0.873	0.968	0.770	1.072	-301.55	0.004	0.00143
3-Bromopyridine	0.873	0.904	0.835	1.071	-302.49	0.003	0.00115
3-Chloropyridine	0.873	0.930	0.809	1.071	-302.42	0.003	0.00114
3,5-Dichloropyridine	0.873	0.968	0.771	1.071	-303.18	0.003	0.00096

[a] *The aromatic rings were placed in the XY plane (in-plane), whereas the π orbitals are located in the out-of-plane (Z-axis). The σ orbitals are assumed to have sp_2-hybridization with equal contribution from 2s, 2px, and 2py.*

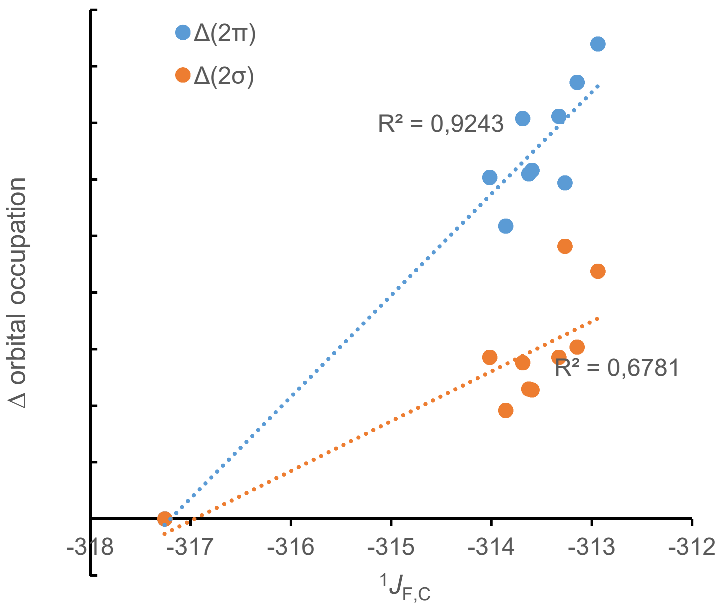

Figure S11: Trends between calculated $^1J_{F,C}$ coupling constants and natural occupation of 2s and 2p orbitals of the meta-C-atom in pentafluoroiodobenzene on halogen bond formation with different pyridine bases.

Table S24. Natural occupation numbers of 2s and 2p orbitals of C atoms in the para-position, calculated at the B3LYP-D3/aug-cc-pVTZ-pp

C_6F_5-I···Base	2s	2px	2py	2pz	$^1J_{F,C}$(Hz)	Δ(SUM(2σ))[a]	Δ(2π)[a]
–	0.875	0.683	1.055	1.050	-312.6	0.000 (reference)	0.000 (reference)
4-Dimethylaminopyridine	0.875	0.680	1.056	1.060	-313.49	0.010	-0.00093
2-Methylpyridine	0.875	0.680	1.056	1.058	-315.01	0.008	-0.00075
2,6-Dimethylpyridine	0.875	0.680	1.056	1.057	-315.63	0.007	-0.00074
4-Methoxypyridine	0.875	0.681	1.055	1.058	-314.41	0.009	-0.00081
3,5-Dimethylpyridine	0.875	0.680	1.056	1.058	-314.41	0.009	-0.00079
Pyridine	0.875	0.680	1.056	1.058	-314.83	0.008	-0.00074
3-Bromopyridine	0.875	0.693	1.043	1.057	-315.51	0.007	-0.00063
3-Chloropyridine	0.875	0.685	1.051	1.057	-315.51	0.007	-0.00067
3,5-Dichloropyridine	0.875	0.681	1.055	1.056	-316.13	0.006	-0.00056

[a] *The aromatic rings were placed in the XY plane (in-plane), whereas the π orbitals are located in the out-of-plane (Z-axis). The σ orbitals are assumed to have sp_2-hybridization with equal contribution from 2s, 2px, and 2py.*

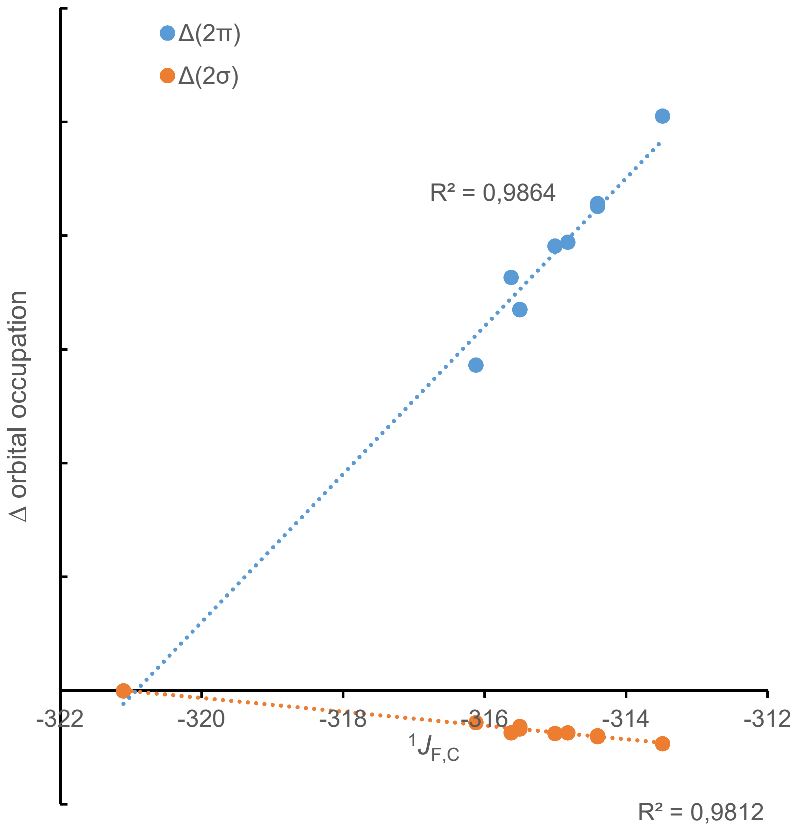

Figure S12: Trends between calculated $^1J_{F,C}$ coupling constants and natural occupation of 2s and 2p orbitals of the para-C-atom in iodopentafluorobenzene on halogen bond formation with different pyridine bases.

Table S25. Bond distances (R), ^{19}F-^{13}C-^{1}J-coupling constants ($J_{F,C}$), binding energies (ΔE), electron densities (ρ) and energy densities (H) at the C-F and N···I bond critical point, calculated at the B3LYP-D3/aug-cc-pVTZ-pp

C_8F_{17}-I···Base	R_{C-F} (Å)	R_{C-I} (Å)	$R_{N\cdots I}$ (Å)	$^1J_{F,C}$ (Hz)	ΔE (kJ/mol)	ρ_{C-F} (e/Å^3)	H_{C-F} (h/Å^3)	$\rho_{N\cdots I}$ (e/Å^3)	$H_{N\cdots I}$ (h/Å^3)
–	1.343	2.186	-	-401.43	-	1.898	-2.870	-	-
4-Dimethylaminopyridine	1.359	2.219	2.685	-388.16	-34.86	1.812	-2.682	0.246	-0.018
2-Methylpyridine	1.356	2.209	2.785	-388.35	-31.38	1.828	-2.717	0.203	-0.005
2,6-Dimethylpyridine	1.355	2.205	2.872	-389.69	-30.65	1.833	-2.729	0.173	+0.001
4-Methoxypyridine	1.357	2.211	2.736	-388.50	-31.06	1.823	-2.705	0.222	-0.010
3,5-Dimethylpyridine	1.357	2.212	2.738	-389.12	-31.35	1.823	-2.707	0.222	-0.010
Pyridine	1.356	2.209	2.761	-389.55	-29.26	1.828	-2.717	0.211	-0.007
3-Bromopyridine	1.354	2.204	2.805	-390.12	-26.34	1.837	-2.736	0.192	-0.002
3-Chloropyridine	1.354	2.204	2.806	-390.23	-26.23	1.837	-2.736	0.191	-0.002
3,5-Dichloropyridine	1.353	2.199	2.848	-390.97	-23.79	1.844	-2.735	0.175	0.001

Table S26. Hybridization characters and natural population charge analysis (X) of C, F, N, and I atoms, calculated at the B3LYP-D3/aug-cc-pVTZ-pp

C_8F_{17}-I···Base	sp^n C_{ortho}	sp^n F_{ortho}	sp^n C	sp^{n*} I	X_C	X_I	$X_{F(ortho)}$	X_N
–	$sp^{3.27}$	$sp^{2.49}$	$sp^{3.39}$	$sp^{15.70}$	+0.5622	+0.1509	-0.3370	-
4-Dimethylaminopyridine	$sp^{3.41}$	$sp^{2.56}$	$sp^{2.96}$	$sp^{30.73}$	+0.4959	+0.1785	-0.3577	-0.5205
2-Methylpyridine	$sp^{3.39}$	$sp^{2.55}$	$sp^{3.02}$	$sp^{25.70}$	+0.5074	+0.1738	-0.3530	-0.4758
2,6-Dimethylpyridine	$sp^{3.38}$	$sp^{2.55}$	$sp^{3.03}$	$sp^{23.84}$	+0.5118	+0.1718	-0.3516	-0.4884
4-Methoxypyridine	$sp^{3.40}$	$sp^{2.55}$	$sp^{3.00}$	$sp^{27.38}$	+0.5031	+0.1775	-0.3546	-0.4948
3,5-Dimethylpyridine	$sp^{3.39}$	$sp^{2.55}$	$sp^{3.00}$	$sp^{27.29}$	+0.5035	+0.1761	-0.3544	-0.4568
Pyridine	$sp^{3.39}$	$sp^{2.55}$	$sp^{3.02}$	$sp^{26.16}$	+0.5067	+0.1759	-0.3531	-0.4656
3-Bromopyridine	$sp^{3.37}$	$sp^{2.55}$	$sp^{3.05}$	$sp^{24.01}$	+0.5125	+0.1747	-0.3505	-0.4453
3-Chloropyridine	$sp^{3.37}$	$sp^{2.55}$	$sp^{3.05}$	$sp^{23.99}$	+0.5125	+0.1748	-0.3505	-0.4456
3,5-Dichloropyridine	$sp^{3.37}$	$sp^{2.53}$	$sp^{3.09}$	$sp^{22.35}$	+0.5206	+0.1733	-0.3500	-0.4263

*Due to the use of effective core potential for describing relativistic effects of I atom

Table S27. Decomposition terms of calculated $^1J_{F,C}$ coupling constants into Fermi contact (FC), spin-dipolar (SD), paramagnetic spin-orbital (PSO), diamagnetic spin-orbit (DSO), and the total $^1J_{F,C}$ coupling constants at the carbon α position as well as the second-order perturbation of the Fock Matrix between σ orbital of C-I and σ orbital of C-F and lone pair of I with σ^* of C-F orbital, calculated at the B3LYP-D3/aug-cc-pVTZ-pp*

C_8F_{17}-I···Base	Fermi Contact	Spin-Dipolar	Paramagnetic Spin-orbit	Diamagnetic Spin-orbit	$^1J_{F,C}$(Hz)	C-I → C-F (kcal/mol)	Lp(I)→ C-F
–	-372.42	4.36	-34.92	1.55	-401.43	1.59	4.76
4-Dimethylaminopyridine	-372.44	7.34	-24.56	1.50	-388.16	1.40	4.36
2-Methylpyridine	-370.40	6.84	-26.30	1.51	-388.35	1.38	4.44
2,6-Dimethylpyridine	-370.93	6.65	-26.92	1.52	-389.69	1.36	4.58
4-Methoxypyridine	-371.34	7.03	-25.70	1.51	-388.50	1.39	3.77
3,5-Dimethylpyridine	-371.87	6.99	-25.75	1.51	-389.12	1.40	4.26
Pyridine	-371.67	6.86	-26.25	1.51	-389.55	1.39	4.35
3-Bromopyridine	-371.01	6.60	-27.22	1.51	-390.12	1.39	4.46
3-Chloropyridine	-371.11	6.60	-27.23	1.51	-390.23	1.39	4.35
3,5-Dichloropyridine	-370.75	6.36	-28.10	1.51	-390.97	1.38	4.53

Table S28. Decomposition terms of calculated $^{1}J_{F,C}$ coupling constants into Fermi contact (FC), spin-dipolar (SD), paramagnetic spin-orbital (PSO), diamagnetic spin-orbit (DSO), and the total $^{1}J_{F,C}$ coupling constants at the carbon β position, calculated at the B3LYP-D3/aug-cc-pVTZ-pp

C_8F_{17}-I···Base	Fermi Contact	Spin-Dipolar	Paramagnetic Spin-orbit	Diamagnetic Spin-orbit	$^{1}J_{F,C}$(Hz)
–	-315.14	10.27	-22.61	1.74	-325.74
4-Dimethylaminopyridine	-311.45	10.87	-20.91	1.61	-319.88
2-Methylpyridine	-312.53	10.74	-21.30	1.61	-321.47
2,6-Dimethylpyridine	-312.70	10.70	-21.44	1.61	-321.83
4-Methoxypyridine	-312.16	10.77	-21.20	1.61	-320.98
3,5-Dimethylpyridine	-312.23	10.78	-21.17	1.61	-321.02
Pyridine	-312.69	10.75	-21.28	1.61	-321.62
3-Bromopyridine	-313.33	10.67	-21.52	1.61	-322.58
3-Chloropyridine	-313.39	10.68	-21.51	1.61	-322.62
3,5-Dichloropyridine	-313.38	10.61	-21.70	1.61	-323.35

Table S29. Calculated ^{19}F coordination shifts ($\Delta\delta = \delta_{F(complex)} - \delta_{F(free)}$) in ppm

C_8F_{17}-I···Base	$\Delta\delta_{alpha}$	$\Delta\delta_{beta}$
–	114.72	49.42
4-Dimethylaminopyridine	88.71	44.73
2-Methylpyridine	93.33	45.05
2,6-Dimethylpyridine	95.09	45.17
4-Methoxypyridine	91.69	44.96
3,5-Dimethylpyridine	91.69	44.92
Pyridine	92.93	45.03
3-Bromopyridine	95.42	45.23
3-Chloropyridine	95.40	45.22
3,5-Dichloropyridine	97.54	45.41

Table S30. Natural occupation numbers of 2s and 2p orbitals of C atoms in the α-position, calculated at the B3LYP-D3/aug-cc-pVTZ-pp

C_8F_{17}-I···Base	2s	2px	2py	2pz	$^{1}J_{F,C}$(Hz)	Δ(2s)	$\Delta(2p_{\sigma})$ [a]
–	1.031	1.077	0.780	0.638	-401.43	0	0
4-Dimethylaminopyridine	1.061	1.136	0.797	0.594	-388.16	0.03	0.032
4-Methoxypyridine	1.057	1.130	0.797	0.599	-388.5	0.026	0.031
Pyridine	1.054	1.125	0.802	0.597	-389.55	0.023	0.029
3-Bromopyridine	1.051	1.118	0.770	0.634	-390.12	0.02	0.027
3-Chloropyridine	1.051	1.120	0.792	0.609	-390.23	0.02	0.026

[a] *We separated the contibutions of the 2s and sum of $2p_x + 2p_y + 2p_z = 3 \times 2p_{\sigma}$ as independent contibutors.*

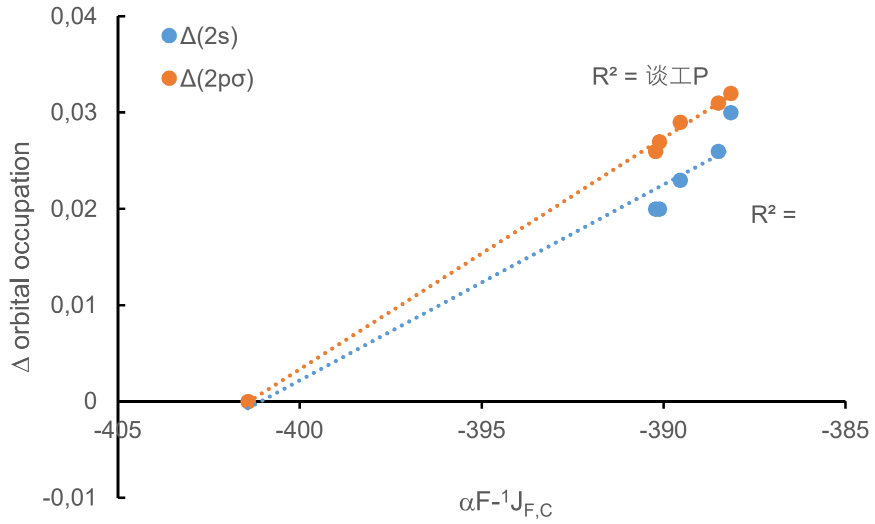

Figure S13: Trends between calculated $^1J_{F,C}$ coupling constants and natural occupation of 2s and $2p_\sigma$ orbitals of the α-C-atom in iodoperfluorooctane on halogen bond formation with different pyridine bases.

Table S31. Natural occupation numbers of 2s and 2p orbitals of C atoms in the β-position, calculated at the B3LYP-D3/aug-cc-pVTZ-pp

C_8F_{17}-I⋯Base	2s	2px	2py	2pz	$^1J_{F,C}$(Hz)	Δ(2s)	$\Delta(2p_\sigma)$ [a]
–	0.936	1.061	0.781	0.621	-325.74	-	-
4-Dimethylaminopyridine	0.940	1.046	0.818	0.602	-319.88	0.004	0.003
4-Methoxypyridine	0.939	1.047	0.813	0.604	-321.47	0.003	0.001
Pyridine	0.939	1.054	0.806	0.604	-321.62	0.003	0.001
3-Bromopyridine	0.938	1.030	0.783	0.651	-322.58	0.002	0.001
3-Chloropyridine	0.938	1.042	0.800	0.622	-322.62	0.002	0.001

[a] *We separated the contibutions of the 2s and sum of $2p_x + 2p_y + 2p_z = 3 \times 2p_\sigma$ as independent contibutors.*

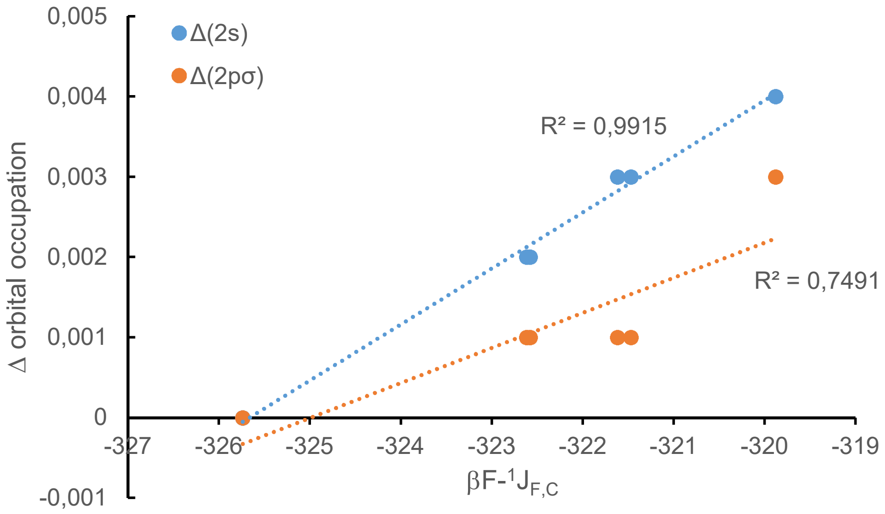

Figure S14: Trends between calculated $^{1}J_{F,C}$ coupling constants and natural occupation of 2s and 2p$_{\sigma}$ orbitals of the β-C-atom in iodoperfluorooctane on halogen bond formation with different pyridine bases.

Table S32. Bond distances (R), $^{1}J_{F,C}$-coupling constants ($J_{F,C}$), binding energies (ΔE), electron densities (ρ) and energy densities (H) at the C-F and N···I bond critical point, calculated at the B3LYP-D3/aug-cc-pVTZ-pp

C_6F_5-I···Base	R_{C-F} (Å)	R_{C-I} (Å)	$R_{N\cdots I}$ (Å)	ortho$^{1}J_{F,C}$ (Hz)	ΔE (kJ/mol)	ρ_{C-F} (e/Å^3)	H_{C-F} (h/Å^3)	$\rho_{N\cdots I}$ (e/Å^3)	$H_{N\cdots I}$ (h/Å^3)
–	1.337	2.099	-	-312.60	-	1.838	-2.716	-	-
NEt_2H	1.343	2.154	2.751	-299.31	-35.72	1.806	-2.649	0.227	-0.014
$NBuH_2$	1.343	2.150	2.758	-298.99	-29.86	1.807	-2.650	0.217	-0.010
Piperidine	1.344	2.158	2.720	-298.51	-37.14	1.804	-2.644	0.239	-0.017
N(*sec*-Bu)H_2	1.343	2.148	2.795	-298.93	-31.46	1.808	-2.652	0.203	-0.006
Pyrrolidine	1.343	2.155	2.741	-299.03	-37.37	1.805	-2.646	0.230	-0.015
N(*iso*-Pr)H_2	1.342	2.144	2.864	-299.62	-34.05	1.810	-2.656	0.181	-0.003
NEt(*iso*-Pr)H	1.343	2.149	2.816	-299.64	-36.17	1.808	-2.652	0.200	-0.007
N(*tert*-Bu)H_2	1.343	2.148	2.789	-298.98	-31.65	1.808	-2.652	0.205	-0.007
NBu_2H	1.343	2.151	2.782	-300.08	-38.52	1.807	-2.651	0.214	-0.010
NPr_2H	1.343	2.150	2.787	-300.13	-37.40	1.808	-2.652	0.211	-0.009
N(*iso*-Pr)$_2$H	1.340	2.121	3.163	-303.98	-21.65	1.822	-2.682	0.108	0.005
NEt_3	1.342	2.137	2.941	-301.67	-32.26	1.814	-2.665	0.160	0.001
NBu_3	1.343	2.152	2.794	-299.67	-42.67	1.807	-2.650	0.214	-0.012
NPr_3	1.337	2.102	3.413	-307.48	-14.87	1.836	-2.711	0.059	0.007

Table S33. Hybridization characters and natural population charge analysis (X) of C, F, N, and I atoms, calculated at the B3LYP-D3/aug-cc-pVTZ-pp

C_6F_5-I···Base	sp^n C	sp^n F	sp^n C	sp^n I	X_C	X_I	X_F	X_N
–	$sp^{3.19}$	$sp^{2.49}$	$sp^{2.96}$	$sp^{10.24}$	-0.2931	+0.2517	-0.3075	-
NEt_2H	$sp^{3.26}$	$sp^{2.50}$	$sp^{2.81}$	$sp^{17.51}$	-0.3156	+0.2427	-0.3183	-0.6475
$NBuH_2$	$sp^{3.26}$	$sp^{2.49}$	$sp^{2.80}$	$sp^{17.61}$	-0.3173	+0.2451	-0.3181	-0.8159
Piperidine	$sp^{3.27}$	$sp^{2.50}$	$sp^{2.80}$	$sp^{18.43}$	-0.3174	+0.2424	-0.3192	-0.6411
N(*sec*-Bu)H_2	$sp^{3.26}$	$sp^{2.50}$	$sp^{2.80}$	$sp^{17.16}$	-0.3163	+0.2440	-0.3177	-0.8258
Pyrrolidine	$sp^{3.27}$	$sp^{2.50}$	$sp^{2.81}$	$sp^{17.79}$	-0.3168	+0.2412	-0.3187	-0.6529
N(*iso*-Pr)H_2	$sp^{3.25}$	$sp^{2.50}$	$sp^{2.81}$	$sp^{16.00}$	-0.3138	+0.2413	-0.3169	-0.6545
NEt(*iso*-Pr)H	$sp^{3.26}$	$sp^{2.50}$	$sp^{2.81}$	$sp^{16.64}$	-0.3149	+0.2418	-0.3175	-0.6498
N(*tert*-Bu)H_2	$sp^{3.26}$	$sp^{2.50}$	$sp^{2.80}$	$sp^{17.30}$	-0.3163	+0.2450	-0.3177	-0.8290
NBu_2H	$sp^{3.26}$	$sp^{2.50}$	$sp^{2.80}$	$sp^{16.91}$	-0.3174	+0.2437	-0.3185	-0.6420
NPr_2H	$sp^{3.26}$	$sp^{2.50}$	$sp^{2.80}$	$sp^{16.78}$	-0.3157	+0.2441	-0.3177	-0.6425
N(*iso*-Pr)$_2$H	$sp^{3.22}$	$sp^{2.50}$	$sp^{2.85}$	$sp^{12.63}$	-0.3069	+0.2501	-0.3131	-0.5287
NEt_3	$sp^{3.24}$	$sp^{2.50}$	$sp^{2.82}$	$sp^{14.39}$	-0.3125	+0.2438	-0.3160	-0.5289
NBu_3	$sp^{3.26}$	$sp^{2.50}$	$sp^{2.81}$	$sp^{16.57}$	-0.3154	+0.2421	-0.3183	-0.5087
NPr_3	$sp^{3.19}$	$sp^{2.50}$	$sp^{2.92}$	$sp^{10.53}$	-0.2964	+0.2496	-0.3081	+0.0637

*Due to the use of effective core potential for describing relativistic effects of I atom

Table S34. Decomposition terms of calculated $^1J_{F,C}$ coupling constants into Fermi contact (FC), spin-dipolar (SD), paramagnetic spin-orbital (PSO), diamagnetic spin-orbit (DSO), and the total $^1J_{F,C}$ coupling constants at the ortho position as well as the second-order perturbation of the Fock Matrix between σ orbital of C-I and σ orbital of C-F, calculated at the B3LYP-D3/aug-cc-pVTZ-pp*

C_6F_5-I···Base	Fermi Contact	Spin-Dipolar	Paramagnetic Spin-orbit	Diamagnetic Spin-orbit	$^1J_{F,C}$(Hz)	C-I → C-F (kcal/mol)
–	-310.78	3.96	-6.94	1.17	-312.60	1.33
NEt_2H	-300.69	4.83	-4.63	1.18	-299.31	1.69
$NBuH_2$	-300.28	4.82	-4.69	1.17	-298.99	1.68
Piperidine	-300.13	4.90	-4.46	1.18	-298.51	1.73
N(*sec*-Bu)H_2	-300.10	4.78	-4.79	1.18	-298.93	1.65
Pyrrolidine	-300.53	4.87	-4.54	1.18	-299.03	1.70
N(*iso*-Pr)H_2	-300.69	4.75	-4.86	1.18	-299.62	1.61
NEt(*iso*-Pr)H	-300.87	4.79	-4.75	1.18	-299.64	1.63
N(*tert*-Bu)H_2	-300.16	4.79	-4.79	1.18	-298.98	1.65
NBu_2H	-301.36	4.81	-4.71	1.19	-300.08	1.60
NPr_2H	-301.37	4.80	-4.74	1.18	-300.13	1.64
N(*iso*-Pr)$_2$H	-303.80	4.41	-5.77	1.19	-303.98	1.42
NEt_3	-302.31	4.63	-5.17	1.19	-301.67	1.51
NBu_3	-300.99	4.81	-4.68	1.19	-299.67	1.66
NPr_3	-305.94	4.04	-6.77	1.19	-307.48	1.31

Table S35. Decomposition terms of calculated $^1J_{F,C}$ coupling constants into Fermi contact (FC), spin-dipolar (SD), paramagnetic spin-orbital (PSO), diamagnetic spin-orbit (DSO), and the total $^1J_{F,C}$ coupling constants at the meta position, calculated at the B3LYP-D3/aug-cc-pVTZ-pp

C_6F_5-I···Base	Fermi Contact	Spin-Dipolar	Paramagnetic Spin-orbit	Diamagnetic Spin-orbit	$^1J_{F,C}$(Hz)
–	-320.78	4.86	-2.50	1.15	-317.26
NEt_2H	-319.81	5.55	-0.64	1.16	-313.75
$NBuH_2$	-319.18	5.53	-0.67	1.16	-313.16
Piperidine	-319.76	5.59	-0.52	1.16	-313.54
N(*sec*-Bu)H_2	-319.06	5.52	-0.71	1.16	-313.10
Pyrrolidine	-319.76	5.56	-0.61	1.16	-313.65
N(*iso*-Pr)H_2	-319.92	5.48	-0.83	1.16	-314.11
NEt(*iso*-Pr)H	-319.96	5.51	-0.74	1.16	-314.04
N(*tert*-Bu)H_2	-319.20	5.52	-0.72	1.16	-313.25
NBu_2H	-319.99	5.53	-0.70	1.16	-314.00
NPr_2H	-319.92	5.52	-0.72	1.16	-313.96
N(*iso*-Pr)$_2$H	-320.10	5.24	-1.48	1.16	-315.18
NEt_3	-320.20	5.40	-1.05	1.16	-314.69
NBu_3	-320.50	5.53	-0.71	1.16	-314.52
NPr_3	-319.62	4.89	-2.43	1.16	-316.00

Table S36. Decomposition terms of calculated $^1J_{F,C}$ coupling constants into Fermi contact (FC), spin-dipolar (SD), paramagnetic spin-orbital (PSO), diamagnetic spin-orbit (DSO), and the total $^1J_{F,C}$ coupling constants at the para position, calculated at the B3LYP-D3/aug-cc-pVTZ-pp

C_6F_5-I···Base	Fermi Contact	Spin-Dipolar	Paramagnetic Spin-orbit	Diamagnetic Spin-orbit	$^1J_{F,C}$(Hz)
–	-320.92	4.12	-5.45	1.15	-321.11
NEt_2H	-317.09	4.92	-2.76	1.15	-313.76
$NBuH_2$	-317.03	4.92	-2.81	1.15	-313.77
Piperidine	-316.91	4.99	-2.59	1.15	-313.36
N(*sec*-Bu)H_2	-316.92	4.90	-2.86	1.15	-313.73
Pyrrolidine	-317.03	4.96	-2.70	1.15	-313.62
N(*iso*-Pr)H_2	-317.22	4.86	-2.97	1.15	-314.18
NEt(*iso*-Pr)H	-317.40	4.90	-2.88	1.15	-314.23
N(*tert*-Bu)H_2	-316.94	4.90	-2.87	1.15	-313.75
NBu_2H	-317.63	4.92	-2.82	1.15	-314.39
NPr_2H	-317.63	4.90	-2.86	1.15	-314.44
N(*iso*-Pr)$_2$H	-318.34	4.57	-3.93	1.15	-316.56
NEt_3	-317.66	4.77	-3.28	1.15	-315.02
NBu_3	-317.84	4.92	-2.80	1.15	-314.56
NPr_3	-319.29	4.15	-5.40	1.15	-319.39

Table S37. Calculated ^{19}F coordination shifts ($\Delta\delta = \delta_{F(complex)} - \delta_{F(free)}$) in ppm

C_6F_5-I···Base	$\Delta\delta_{ortho}$	$\Delta\delta_{meta}$	$\Delta\delta_{para}$
–	43.55	2.48	10.50
NEt_2H	40.29	0.70	6.51
$NBuH_2$	40.00	0.57	6.53
Piperidine	40.09	0.53	6.34
N(*sec*-Bu)H_2	39.94	0.62	6.44
Pyrrolidine	40.05	0.59	6.57
N(*iso*-Pr)H_2	40.12	0.88	6.72
NEt(*iso*-Pr)H	40.16	0.82	6.62
N(*tert*-Bu)H_2	40.04	0.66	6.51
NBu_2H	40.53	0.75	6.76
NPr_2H	40.35	0.76	6.78
N(*iso*-Pr)$_2$H	42.62	1.65	8.56
NEt_3	41.25	1.20	7.43
NBu_3	41.00	0.88	6.99
NPr_3	43.37	2.86	10.87

Table S38. Bond distances (R), ^{19}F-^{13}C-^{1}J-coupling constants ($J_{F,C}$), binding energies (ΔE), electron densities (ρ) and energy densities (H) at the C-F and N···I bond critical point, calculated at the B3LYP-D3/aug-cc-pVTZ-pp

C_6F_5-I···Base	R_{C-F} (Å)	R_{C-I} (Å)	$R_{O\cdots I}$ (Å)	$^{1}J_{F,C}$ (Hz)	ΔE (kJ/mol)	ρ_{C-F} (e/Å^3)	H_{C-F} (h/Å^3)	$\rho_{N\cdots I}$ (e/Å^3)	$H_{N\cdots I}$ (h/Å^3)
–	1.337	2.099	-	-312.60	-	1.83824	-2.7164	-	-
Pyridine-N-oxide	1.341	2.126	2.755	-302.60	-24.11	1.81786	-2.6731	0.17650	0.0070
Trimethylamin-N-oxide	1.343	2.149	2.626	-298.88	-35.43	1.80468	-2.6452	0.23332	-0.0040
N,N-Dimethylpyrrolidine-2-one	1.340	2.119	2.819	-303.47	-22.53	1.81995	-2.6776	0.14854	0.0115
Triphenylphosphineoxide	1.340	2.118	2.815	-302.10	-30.21	1.81917	-2.6760	0.14806	0.0122
Pyrrolidine-N-oxide	1.340	2.121	2.824	-303.46	-20.69	1.81995	-2.6776	0.15133	0.0112
Dimethylsulfoxide	1.340	2.119	2.829	-302.76	-19.69	1.82050	-2.6787	0.14573	0.0120
Dimethylformamide	1.339	2.112	2.905	-304.89	-17.26	1.82456	-2.6874	0.11977	0.0138
Acetophenone	1.339	2.107	2.982	-305.88	-16.41	1.82822	-2.6952	0.10143	0.0135
Benzophenone	1.339	2.110	2.937	-304.86	-20.70	1.82592	-2.6903	0.11526	0.0130
4-Methylpyridine-N-oxide	1.341	2.129	2.727	-302.40	-26.02	1.81540	-2.6679	0.18865	0.0049

Table S39. Hybridization characters and natural population charge analysis (X) of C, F, N, and I atoms, calculated at the B3LYP-D3/aug-cc-pVTZ-pp

C_6F_5-I⋯Base	sp^n C	sp^n F	sp^n C	sp^n I	X_C	X_I	X_F	X_O
–	$sp^{3.19}$	$sp^{2.49}$	$sp^{2.96}$	$sp^{10.24}$	-0.2931	+0.2517	-0.3075	-
Pyridine-N-oxide	$sp^{3.24}$	$sp^{2.49}$	$sp^{2.82}$	$sp^{14.17}$	-0.3141	+0.2656	-0.3144	-0.6127
Trimethylamin-N-oxide	$sp^{3.27}$	$sp^{2.50}$	$sp^{2.77}$	$sp^{18.33}$	-0.3215	+0.2686	-0.3188	-0.7428
N,N-Dimethylpyrrolidine-2-one	$sp^{3.23}$	$sp^{2.49}$	$sp^{2.82}$	$sp^{13.58}$	-0.3121	+0.2710	-0.3135	-0.7391
Triphenylphosphineoxide	$sp^{3.23}$	$sp^{2.50}$	$sp^{2.79}$	$sp^{13.75}$	-0.3133	+0.2842	-0.3136	-1.1300
Pyrrolidine-N-oxide	$sp^{3.23}$	$sp^{2.49}$	$sp^{2.82}$	$sp^{13.64}$	-0.3131	+0.2662	-0.3135	-0.6797
Dimethylsulfoxide	$sp^{3.23}$	$sp^{2.49}$	$sp^{2.81}$	$sp^{13.58}$	-0.3127	+0.2749	-0.3135	-1.0262
Dimethylformamide	$sp^{3.22}$	$sp^{2.49}$	$sp^{2.83}$	$sp^{12.61}$	-0.3092	+0.2725	-0.3121	-0.6932
Acetophenone	$sp^{3.21}$	$sp^{2.49}$	$sp^{2.85}$	$sp^{11.91}$	-0.3060	+0.2716	-0.3108	-0.6032
Benzophenone	$sp^{3.21}$	$sp^{2.49}$	$sp^{2.84}$	$sp^{12.31}$	-0.3081	+0.2726	-0.3115	-0.5942
4-Methylpyridine-N-oxide	$sp^{3.24}$	$sp^{2.49}$	$sp^{2.81}$	$sp^{14.69}$	-0.3154	+0.2659	-0.3151	-0.6256

*Due to the use of effective core potential for describing relativistic effects of I atom

Table S40. Decomposition terms of calculated $^1J_{F,C}$ coupling constants into Fermi contact (FC), spin-dipolar (SD), paramagnetic spin-orbital (PSO), diamagnetic spin-orbit (DSO), and the total $^1J_{F,C}$ coupling constants at the ortho position as well as the second-order perturbation of the Fock Matrix between σ orbital of C-I and σ orbital of C-F, calculated at the B3LYP-D3/aug-cc-pVTZ-pp*

C_6F_5-I⋯Base	Fermi Contact	Spin-Dipolar	Paramagnetic Spin-orbit	Diamagnetic Spin-orbit	$^1J_{F,C}$(Hz)	C-I → C-F (kcal/mol)
–	-310.78	3.96	-6.94	1.17	-312.60	1.33
Pyridine-N-oxide	-302.78	4.51	-5.52	1.18	-302.60	1.51
Trimethylamin-N-oxide	-300.44	4.88	-4.49	1.18	-298.88	1.67
N,N-Dimethylpyrrolidine-2-one	-303.44	4.45	-5.67	1.18	-303.47	1.47
Triphenylphosphineoxide	-302.14	4.47	-5.62	1.20	-302.10	1.46
Pyrrolidine-N-oxide	-303.47	4.46	-5.64	1.18	-303.46	1.47
Dimethylsulfoxide	-302.64	4.43	-5.73	1.18	-302.76	1.48
Dimethylformamide	-304.39	4.33	-6.01	1.18	-304.89	1.42
Acetophenone	-304.99	4.22	-6.30	1.19	-305.88	1.39
Benzophenone	-304.19	4.28	-6.15	1.19	-304.86	1.41
4-Methylpyridine-N-oxide	-302.89	4.59	-5.28	1.18	-302.40	1.53

Table S41. Decomposition terms of calculated $^1J_{F,C}$ coupling constants into Fermi contact (FC), spin-dipolar (SD), paramagnetic spin-orbital (PSO), diamagnetic spin-orbit (DSO), and the total $^1J_{F,C}$ coupling constants at the meta position, calculated at the B3LYP-D3/aug-cc-pVTZ-pp

C_6F_5-I⋯Base	Fermi Contact	Spin-Dipolar	Paramagnetic Spin-orbit	Diamagnetic Spin-orbit	$^1J_{F,C}$(Hz)
–	-320.78	4.86	-2.50	1.15	-317.26
Pyridine-N-oxide	-318.79	5.35	-1.15	1.16	-313.43
Trimethylamin-N-oxide	-319.13	5.62	-0.43	1.16	-312.78
N,N-Dimethylpyrrolidine-2-one	-318.76	5.31	-1.27	1.16	-313.57
Triphenylphosphineoxide	-318.61	5.35	-1.15	1.17	-313.26
Pyrrolidine-N-oxide	-318.89	5.27	-1.37	1.16	-313.83
Dimethylsulfoxide	-318.53	5.32	-1.23	1.16	-313.29
Dimethylformamide	-318.79	5.21	-1.54	1.16	-313.97
Acetophenone	-318.86	5.14	-1.73	1.16	-314.29
Benzophenone	-318.67	5.20	-1.56	1.16	-313.87
4-Methylpyridine-N-oxide	-318.88	5.40	-1.01	1.16	-313.31

Table S42. Decomposition terms of calculated $^{1}J_{F,C}$ coupling constants into Fermi contact (FC), spin-dipolar (SD), paramagnetic spin-orbital (PSO), diamagnetic spin-orbit (DSO), and the total $^{1}J_{F,C}$ coupling constants at the para position, calculated at the B3LYP-D3/aug-cc-pVTZ-pp

C_6F_5-I···Base	Fermi Contact	Spin-Dipolar	Paramagnetic Spin-orbit	Diamagnetic Spin-orbit	$^{1}J_{F,C}$(Hz)
–	-320.92	4.12	-5.45	1.15	-321.11
Pyridine-N-oxide	-318.05	4.69	-3.49	1.15	-315.70
Trimethylamin-N-oxide	-317.20	5.03	-2.32	1.15	-313.34
N,N-Dimethylpyrrolidine-2-one	-318.17	4.64	-3.64	1.15	-316.02
Triphenylphosphineoxide	-317.95	4.68	-3.44	1.15	-315.55
Pyrrolidine-N-oxide	-318.07	4.59	-3.81	1.15	-316.14
Dimethylsulfoxide	-318.16	4.64	-3.62	1.15	-315.98
Dimethylformamide	-318.67	4.51	-4.05	1.15	-317.07
Acetophenone	-318.70	4.43	-4.35	1.15	-317.47
Benzophenone	-318.58	4.50	-4.10	1.15	-317.02
4-Methylpyridine-N-oxide	-317.88	4.76	-3.22	1.15	-315.19

Table S43. Calculated ^{19}F coordination shifts ($\Delta\delta = \delta_{F(complex)} - \delta_{F(free)}$) in ppm

C_6F_5-I···Base	$\Delta\delta_{ortho}$	$\Delta\delta_{meta}$	$\Delta\delta_{para}$
–	43.55	2.48	10.50
Pyridine-N-oxide	41.48	0.77	7.44
Trimethylamin-N-oxide	39.98	0.21	5.52
N,N-Dimethylpyrrolidine-2-one	41.70	1.05	7.72
Triphenylphosphineoxide	41.58	0.87	7.39
Pyrrolidine-N-oxide	41.42	1.26	8.01
Dimethylsulfoxide	41.58	0.83	7.59
Dimethylformamide	42.10	1.33	8.35
Acetophenone	42.60	1.65	8.89
Benzophenone	42.33	1.42	8.49
4-Methylpyridine-N-oxide	41.16	0.74	6.98

4.1. Decomposition of the computed scalar couplings into FC, SD, PSO and DSO terms

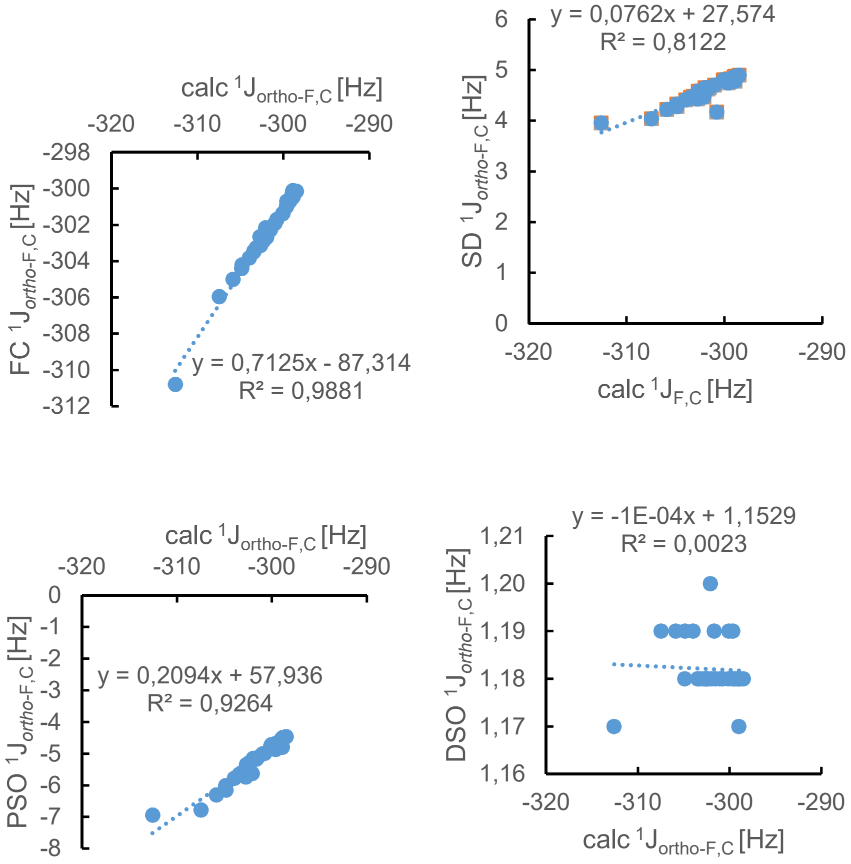

Figure S15: Calculated $^{1}J_{o\text{-}F,C}$ couplings vs it's FC, SD, PSO, DSO contributions for Lewis base-IC_6F_5 adducts.

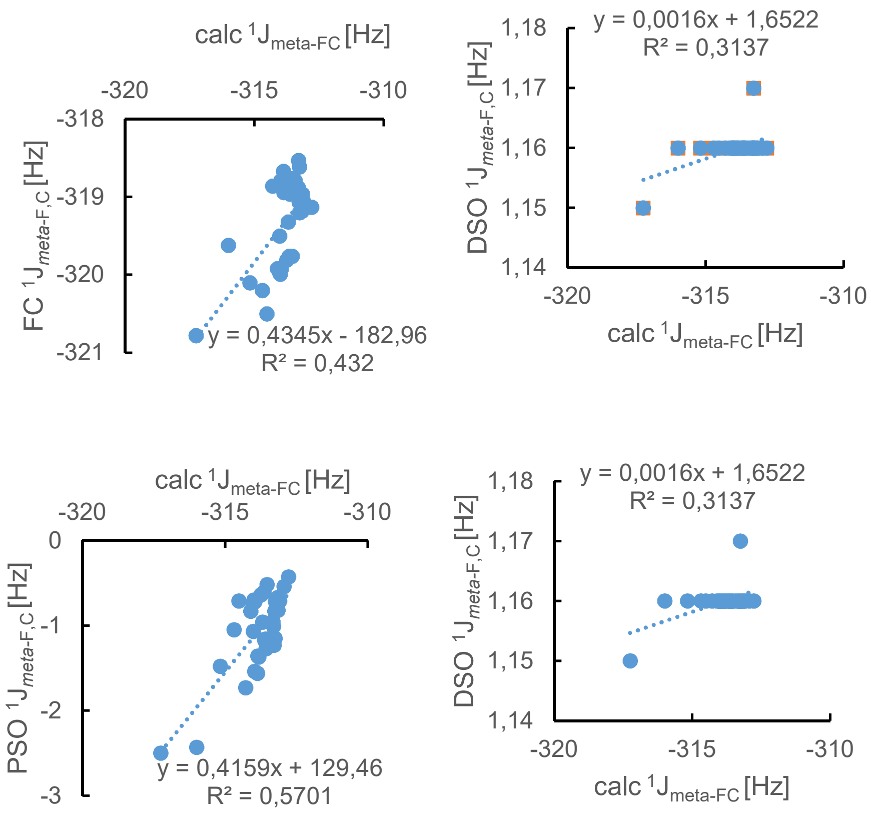

Figure S16: Calculated $^1J_{m\text{-}F,C}$ couplings vs it's FC, SD, PSO, DSO contributions for Lewis base-IC_6F_5 adducts.

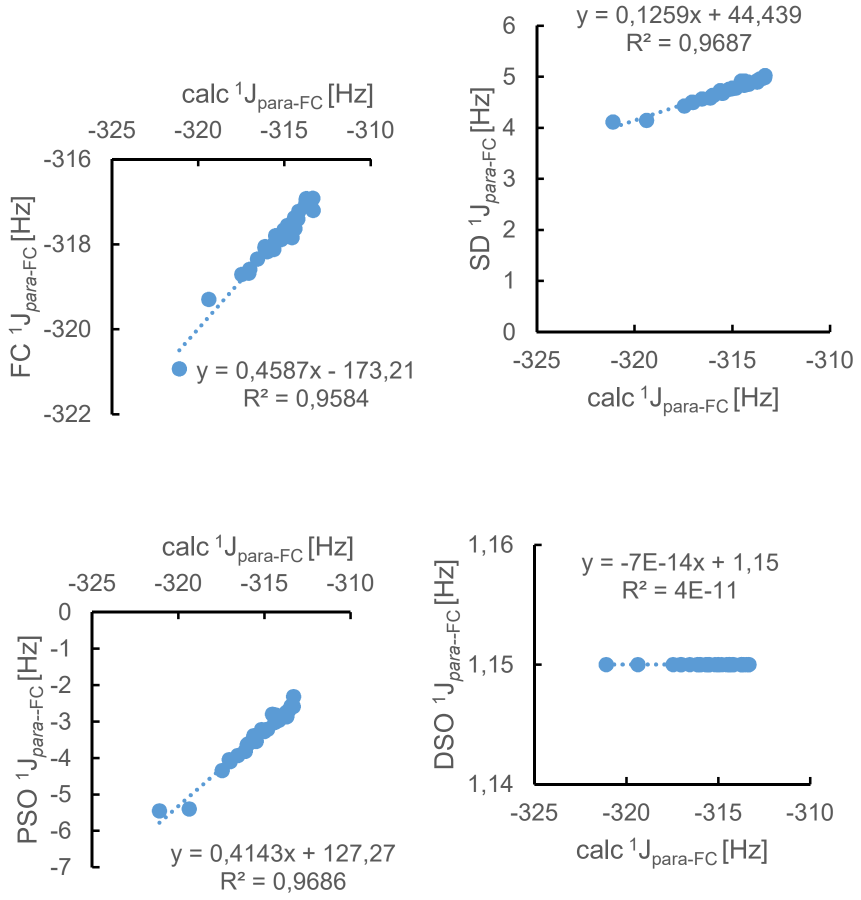

Figure S17: Calculated $^{1}J_{p\text{-}F,C}$ couplings vs it's FC, SD, PSO, DSO contributions for Lewist base-IC_6F_5 adducts.

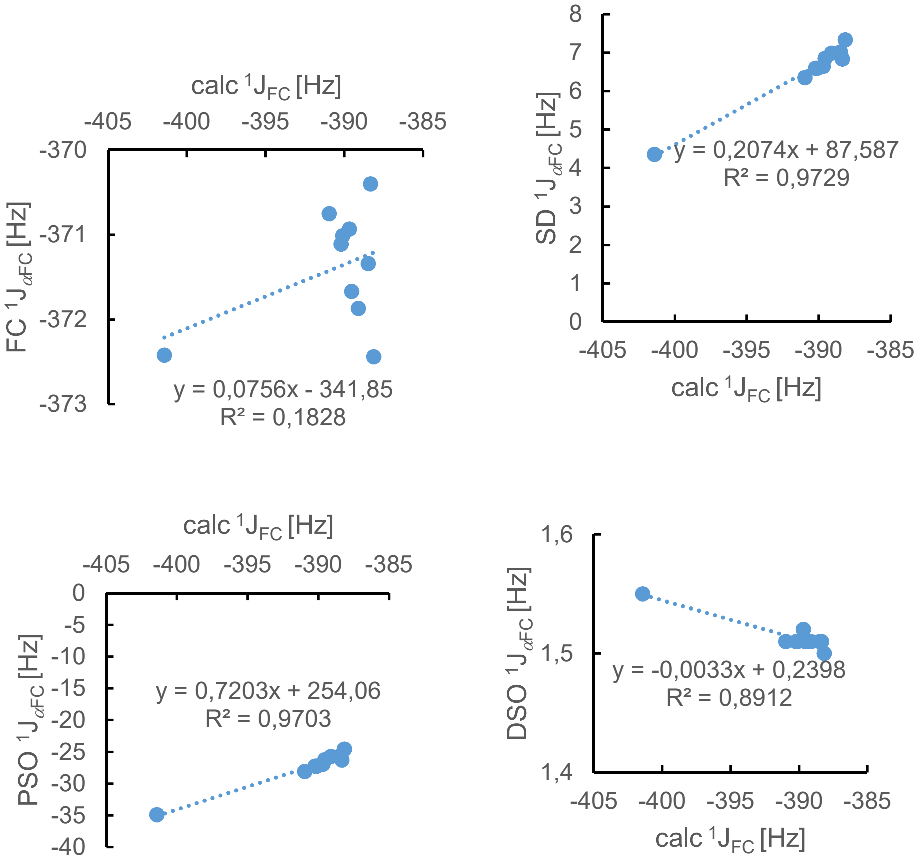

Figure S18: Calculated $^{1}J_{alpha\text{-}F,C}$ couplings vs it's FC, SD, PSO, DSO contributions for Lewis base-IC_8F_{17} adducts.

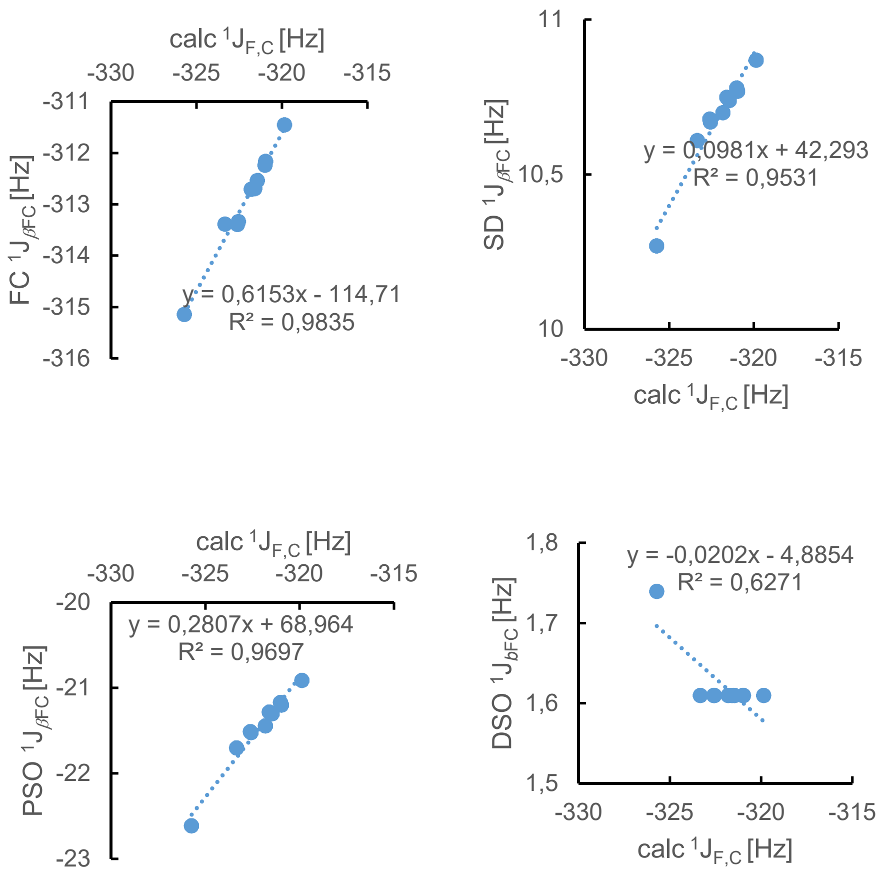

Figure S19: Calculated $^1J_{beta\text{-}F,C}$ couplings vs it's FC, SD, PSO, DSO contributions for Lewis base-IC_8F_{17} adducts.

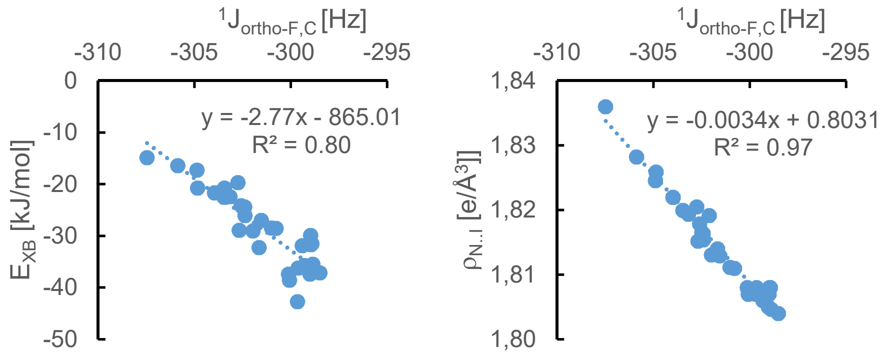

Figure S20: Calculated $^1J_{o\text{-}F,C}$ couplings vs calculated E_{XB} and $\rho_{N..I}$ for Lewis base-IC_5F_5 adducts.

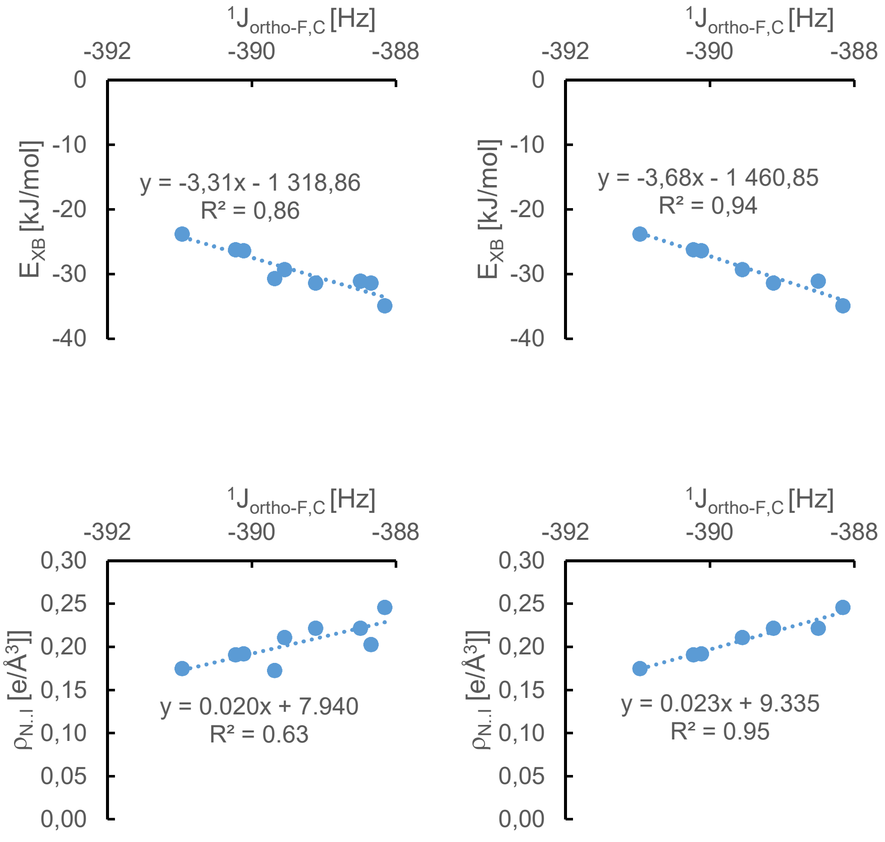

Figure S21: Calculated $^1J_{alpha\text{-}F,C}$ couplings vs calculated E_{XB} and $\rho_{N..I}$ for different pyridine-IC_8F_{17} adducts (left with ortho-substituted pyridines (2,6-dimethylpyridine and 2-picoline), right without those.

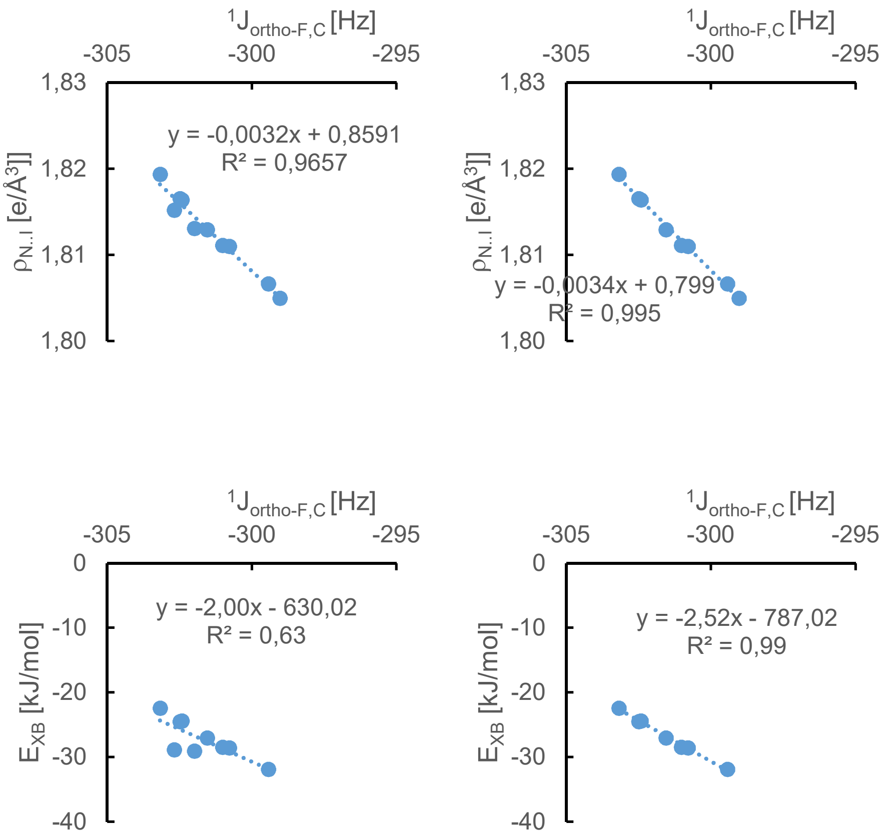

Figure S22: Calculated $^1J_{o\text{-}F,C}$ couplings vs calculated E_{XB} and $\rho_{N..I}$ for pyridine-IC_6F_5 adducts (left with ortho-substituted pyridines (2,6-dimethylpyridine and 2-picoline), right without those.

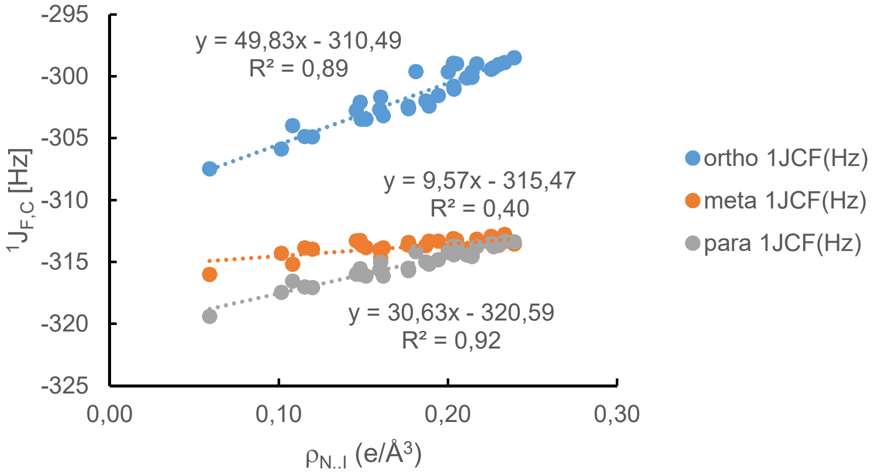

Figure S23: Calculated $^1J_{F,C}$ couplings vs calculated $\rho_{N..I}$ for Lewis base-IC_6F_5 adducts for ortho, meta and para-F.

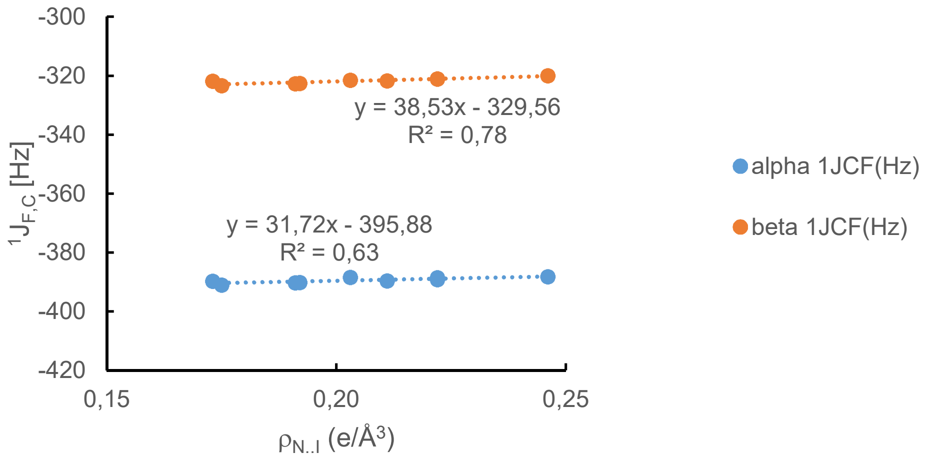

Figure S24: The calculated $^1J_{F,C}$ couplings vs calculated $\rho_{N..I}$ for Lewis base-IC_8F_{17} adducts for alpha and beta-F.

The calculated $^1J_{F,C}$ vs $\rho_{N..I}$ agree with the experimentally observed trends in $\Delta^1J_{F,C}$ vs pK_{bI2}[2] for IC_6F_5 , thus $^1J_{F,C}$ *ortho* > *para* >*meta* (Figure 2 and Figure S23). For IC_8F_{17}, the $\Delta^1J_{F,C}$ follows the trend alpha>beta (Figure 4 and Figure S24). We observed good correlations for $^1J_{F,C}$ for the α-F and β-F of IC_8F_{17} and for the *ortho*-F and *para*-F of IC_6F_5, while weak correlation for the *meta*-F of IC_6F_5 in both the experimental (R^2=0.13 with pK_{BI2}) and calculated (R^2=0.40 with $\rho_{N..I}$) datasets.

5 Optimized Structures Obtained at the B3LYP-D3/aug-cc-pVTZ-pp Level of Theory

Pentafluoroiodobenzene (C_6F_5I)

I	-2.30648000	0.00000000	0.00000000
C	-0.20705400	0.00000200	-0.00000200
C	0.50642600	1.19249200	-0.00000200
C	0.50642800	-1.19249600	-0.00000400
C	1.89344600	1.19960400	-0.00000200
C	1.89344000	-1.19960500	-0.00000100
C	2.58917700	0.00000200	0.00000000
F	-0.12543800	-2.37041800	0.00000000
F	2.56206300	-2.35549100	0.00000100
F	3.92144400	-0.00000600	0.00000200
F	2.56205700	2.35549600	0.00000100
F	-0.12543000	2.37042100	0.00000000

C_6F_5I···4-Dimethylaminopyridine

I	-0.20435100	0.00000400	-0.00004300
C	3.22238500	1.13401200	-0.11049700
C	4.60000000	1.19274500	-0.11634000
C	5.35277500	-0.00000300	0.00002200
C	4.59998700	-1.19274700	0.11633600
C	3.22237300	-1.13400700	0.11041000
N	2.51876700	0.00000400	-0.00006400
H	2.64543800	2.04672000	-0.19929800
H	5.07712700	2.15461500	-0.21020900
H	5.07710300	-2.15462000	0.21023000

H	2.64541600	-2.04671200	0.19917400
C	-2.35213200	0.00000200	-0.00001300
C	-3.07498200	-1.18388400	-0.04014500
C	-3.07498600	1.18388500	0.04014200
C	-4.46200000	-1.19751900	-0.04060100
C	-4.46200300	1.19751400	0.04064800
C	-5.15938300	-0.00000400	0.00003700
F	-2.44617700	2.36983500	0.08052400
F	-5.13305300	2.35517100	0.08013200
F	-6.49482500	-0.00000700	0.00006100
F	-5.13304700	-2.35517900	-0.08006200
F	-2.44617000	-2.36983200	-0.08055100
N	6.70984600	-0.00000400	0.00006500
C	7.44345100	-1.25170100	0.12306100
H	8.50807600	-1.04426900	0.09975800
H	7.21573200	-1.75754800	1.06445500
H	7.21168500	-1.93316400	-0.69896200
C	7.44345900	1.25169100	-0.12290000
H	7.21580800	1.75752200	-1.06431900
H	7.21163300	1.93316600	0.69909500
H	8.50808300	1.04425900	-0.09951700

C_6F_5I···2-Methylpyridine

I	-0.61360800	-0.02975600	-0.00084000
C	-4.25743700	0.88353100	0.01252300
C	-5.64150100	0.70873200	0.01098000
C	-6.17001000	-0.57419600	-0.00692700
C	-5.30361400	-1.65948200	-0.02296800
C	-3.94016200	-1.40432600	-0.02021300
N	-3.42916200	-0.17163400	-0.00300500
H	-7.24103100	-0.72389900	-0.00833500
H	-6.29101700	1.57243000	0.02372900
H	-5.66867200	-2.67599100	-0.03720400
H	-3.22351000	-2.21660500	-0.03222200
C	1.52168200	-0.02872300	-0.00051100
C	2.24429500	-1.21395800	0.00687000
C	2.24141000	1.15828200	-0.00765700
C	3.63131400	-1.22455900	0.00718700
C	3.62836700	1.17239800	-0.00751500
C	4.32688600	-0.02522700	-0.00005000
F	1.61038400	2.34227700	-0.01501600
F	4.29737100	2.33104200	-0.01453100
F	5.66161500	-0.02358400	0.00017300
F	4.30321900	-2.38152200	0.01443100
F	1.61634600	-2.39954100	0.01403000
C	-3.62883700	2.24619000	0.03145600
H	-2.99242100	2.35785400	0.91024500
H	-2.99231500	2.38217900	-0.84381000
H	-4.38044500	3.03251300	0.04232400

$C_6F_5I\cdots$2,6-Dimethylpyridine

I	-0.46438900	-0.00000400	-0.00010800
C	-4.03998200	1.16035700	0.05540300
C	-5.43343300	1.19383900	0.05704800
C	-6.13626100	-0.00000200	0.00028800
C	-5.43344000	-1.19383800	-0.05668600
C	-4.03998900	-1.16034600	-0.05545400
N	-3.36887400	0.00000800	-0.00012200
H	-7.21794100	-0.00000700	0.00044800
H	-5.95043000	2.14159200	0.10211300
H	-5.95044400	-2.14159400	-0.10160500
C	1.66646500	-0.00000400	-0.00002200
C	2.38699200	-1.18612600	0.03754100
C	2.38699100	1.18612100	-0.03751900
C	3.77396600	-1.19802100	0.03804800
C	3.77396600	1.19802200	-0.03789600
C	4.47086500	0.00000200	0.00010900
F	1.75727000	2.36973600	-0.07504300
F	4.44441300	2.35505400	-0.07468800
F	5.80542800	0.00000300	0.00017100
F	4.44441500	-2.35505000	0.07490500
F	1.75727100	-2.36974200	0.07500700
C	-3.22965600	2.42335600	0.11580500
H	-2.58131300	2.41918200	0.99274400
H	-2.58297800	2.50412800	-0.75866200
H	-3.86889700	3.30253800	0.15907700
C	-3.22967600	-2.42334200	-0.11608500
H	-2.58136300	-2.41903800	-0.99304300
H	-2.58296900	-2.50425600	0.75834900
H	-3.86892700	-3.30251100	-0.15946200

$C_6F_5I\cdots$4-Methoxypyridine

I	-0.09648900	-0.06588200	0.00002100
C	-3.58779900	0.97939000	0.00009200
C	-4.97524700	1.00888900	0.00006800
C	-5.66062500	-0.20792500	-0.00002900
C	-4.91265300	-1.39032300	-0.00009500
C	-3.53572100	-1.30536600	-0.00006400
N	-2.86850600	-0.14280900	0.00002800
H	-3.02995800	1.90771800	0.00016600
H	-5.48619500	1.95806800	0.00012400
H	-5.41413000	-2.34685200	-0.00017100
H	-2.93270100	-2.20452900	-0.00011500
C	2.04134100	0.00373300	0.00000500
C	2.80235200	-1.15710200	0.00001800
C	2.72314200	1.21278600	-0.00001800
C	4.18902900	-1.12337800	0.00000600
C	4.10890900	1.27205700	-0.00002900

C	4.84577500	0.09765300	-0.00001700
F	2.05460400	2.37650800	-0.00003000
F	4.74019600	2.45198000	-0.00005200
F	6.17998800	0.14222800	-0.00002800
F	4.89808000	-2.25825300	0.00001800
F	2.21303700	-2.36283500	0.00004000
C	-7.81052100	0.84041800	-0.00000100
H	-7.62641000	1.43905200	0.89316400
H	-7.62637700	1.43916800	-0.89308200
H	-8.83783300	0.49080500	-0.00004300
O	-6.99924200	-0.34045900	-0.00006400

C_6F_5I···3,5-Dimethylpyridine

I	-0.24539600	-0.00002400	-0.00004700
C	-3.69762900	1.14471700	0.08989300
C	-5.08949300	1.20780500	0.09444200
C	-5.77615000	0.00003200	0.00006300
C	-5.08954600	-1.20776700	-0.09438000
C	-3.69768000	-1.14473200	-0.08995600
N	-3.02018900	-0.00002000	-0.00006200
H	-6.86005300	0.00005200	0.00011400
H	-3.10518400	2.04934200	0.16122200
H	-3.10527500	-2.04937900	-0.16133800
C	1.89374700	-0.00000800	-0.00001600
C	2.61546900	-1.18393500	0.06369600
C	2.61545200	1.18393100	-0.06370200
C	4.00246800	-1.19665600	0.06441200
C	4.00245100	1.19667400	-0.06436600
C	4.69960700	0.00001400	0.00003600
F	1.98619200	2.36758100	-0.12761500
F	4.67309500	2.35296100	-0.12680400
F	6.03456600	0.00002600	0.00006200
F	4.67312800	-2.35293200	0.12687800
F	1.98622500	-2.36759600	0.12758800
C	-5.81450700	2.52072900	0.19269600
H	-6.41264200	2.70146800	-0.70204400
H	-6.49606900	2.52603900	1.04453400
H	-5.12031800	3.35163400	0.30812700
C	-5.81462000	-2.52066400	-0.19256800
H	-5.12047400	-3.35159500	-0.30806100
H	-6.41268200	-2.70137800	0.70222600
H	-6.49625900	-2.52594800	-1.04434500

C_6F_5I···Pyridine

I	0.77469200	0.00000200	0.00000200
C	4.25396600	1.14692800	-0.03376600
C	5.64181200	1.19550300	-0.03523700
C	6.34877300	-0.00000300	-0.00000500
C	5.64180900	-1.19550700	0.03523000

C	4.25396300	-1.14692800	0.03376800
N	3.57077900	0.00000100	0.00000200
H	7.42996500	-0.00000500	-0.00000800
H	3.66545400	2.05546000	-0.06050600
H	6.15021900	2.14839300	-0.06334900
H	6.15021300	-2.14839900	0.06334000
H	3.66544900	-2.05545800	0.06051000
C	-1.36095800	0.00000000	0.00000100
C	-2.08226800	-1.18604700	-0.00750300
C	-2.08227000	1.18604700	0.00750300
C	-3.46926200	-1.19843700	-0.00759400
C	-3.46926400	1.19843500	0.00759200
C	-4.16635200	-0.00000200	-0.00000200
F	-1.45286800	2.37090800	0.01501100
F	-4.13965200	2.35628100	0.01496000
F	-5.50109300	-0.00000300	-0.00000300
F	-4.13964800	-2.35628500	-0.01496300
F	-1.45286500	-2.37090700	-0.01501000

C_6F_5I···3-Bromopyridine

I	-0.38357600	-0.39959300	-0.00000100
C	3.28030000	0.07896200	0.00001000
C	4.65131600	-0.14604000	-0.00000300
C	5.13832200	-1.44470400	-0.00003600
C	4.21790000	-2.48437300	-0.00005500
C	2.86307200	-2.18162000	-0.00004100
N	2.40723100	-0.92736600	-0.00000800
H	6.20056800	-1.63901800	-0.00004600
H	2.87823100	1.08313300	0.00003600
H	4.54812700	-3.51287500	-0.00008100
H	2.11652100	-2.96498700	-0.00005500
C	-2.47637100	-0.00856200	0.00000000
C	-3.40187600	-1.04353100	0.00001900
C	-2.96706400	1.29027400	-0.00001800
C	-4.76753000	-0.80109200	0.00002000
C	-4.32849100	1.55570500	-0.00001700
C	-5.23306700	0.50491700	0.00000200
F	-2.13098000	2.33860100	-0.00003700
F	-4.77542800	2.81634800	-0.00003400
F	-6.54488000	0.74924400	0.00000300
F	-5.63837300	-1.81634000	0.00003900
F	-2.99935700	-2.32270200	0.00003600
Br	5.84793100	1.33993600	0.00002500

C_6F_5I···3-Chloropyridine

I	-0.19175600	-0.25117100	0.00003000
C	-3.81263300	0.49472300	-0.00009100
C	-5.19629600	0.36904100	-0.00006500
C	-5.77574500	-0.89082500	0.00013600

C	-4.93253300	-1.99341400	0.00030400
C	-3.55915700	-1.78929300	0.00026600
N	-3.01408400	-0.57102000	0.00007200
H	-6.84984600	-1.00302800	0.00015800
H	-3.34424800	1.46986500	-0.00024500
H	-5.33558800	-2.99553200	0.00046300
H	-2.87110400	-2.62441600	0.00039300
C	1.92384300	-0.01271700	0.00000600
C	2.77274400	-1.11139500	-0.00010700
C	2.50649600	1.24753900	0.00010100
C	4.15229200	-0.96751900	-0.00012600
C	3.88341200	1.41466200	0.00008400
C	4.71035000	0.30173300	-0.00003000
F	1.74777800	2.35317400	0.00021300
F	4.41958200	2.64004800	0.00017600
F	6.03631700	0.45149500	-0.00004700
F	4.94798700	-2.04269100	-0.00023600
F	2.27948900	-2.35841400	-0.00020200
Cl	-6.19144600	1.80556400	-0.00028500

C_6F_5I···3,5-Dichloropyridine

I	0.25535000	0.00007600	0.00000500
C	-3.29253100	1.15122800	-0.00035200
C	-4.68196400	1.18338900	-0.00036200
C	-5.40405000	-0.00006600	-0.00000300
C	-4.68186100	-1.18345800	0.00035800
C	-3.29243100	-1.15117600	0.00035200
N	-2.62481500	0.00005500	0.00000100
H	-6.48301200	-0.00011300	-0.00000400
H	-2.71477100	2.06506700	-0.00063100
H	-2.71459200	-2.06496500	0.00063200
C	2.37934800	0.00002500	0.00000400
C	3.09892800	-1.18764500	-0.00044500
C	3.09898800	1.18766100	0.00044800
C	4.48589800	-1.19876700	-0.00045300
C	4.48595800	1.19871100	0.00044800
C	5.18269300	-0.00004600	-0.00000500
F	2.46893500	2.37074000	0.00089500
F	5.15580200	2.35605900	0.00088200
F	6.51675200	-0.00007800	-0.00000800
F	5.15568200	-2.35615000	-0.00089100
F	2.46881600	-2.37069400	-0.00088800
Cl	-5.51232900	2.71645200	-0.00082500
Cl	-5.51209400	-2.71659400	0.00081900

1-Iodoheptadecafluorooctane ($C_8F_{17}I$)

C	5.70116600	-0.27311400	0.21789800
C	4.37915700	0.17505500	-0.48909500
C	3.07591900	-0.32417500	0.22418500

C	1.78848100	0.44684300	-0.23909700
C	0.45936500	-0.31327100	0.11216400
C	-0.80790100	0.61601700	0.06242200
C	-2.15420200	-0.19082300	-0.04473700
C	-3.42098800	0.65203400	0.31423300
F	5.68644500	-1.58981500	0.44309500
F	5.86305400	0.36684100	1.37592300
F	6.73297700	0.01766700	-0.57826800
F	4.40099200	-0.31070900	-1.74402700
F	4.37807400	1.52284600	-0.53870700
F	2.92528400	-1.63707400	-0.04259200
F	3.21683000	-0.16420800	1.55488700
F	1.84140900	0.62135300	-1.57422500
F	1.77675300	1.65359700	0.36053100
F	0.30268400	-1.32331800	-0.76539300
F	0.56418400	-0.82526700	1.35461800
F	-0.71410300	1.42810300	-1.00995600
F	-0.82189800	1.37073900	1.17759000
F	-2.26686000	-0.63502500	-1.31075000
F	-2.09903300	-1.24809500	0.79115600
F	-3.38809600	1.80434000	-0.37864500
F	-3.40317700	0.94728000	1.62320600
I	-5.26576700	-0.42876800	-0.14327900

$C_8F_{17}I$···4-Methylaminopyridine

C	-7.70933300	1.00414500	-0.34152100
C	-6.44816200	0.60603500	0.49493900
C	-5.12273500	0.55430800	-0.33963800
C	-3.96645400	-0.22010800	0.38842500
C	-2.54770200	0.09143500	-0.20982000
C	-1.46365100	-0.97369900	0.19018600
C	0.00953100	-0.46449000	-0.00896300
C	1.09303100	-1.58053500	-0.02460500
F	-8.05385300	0.02684300	-1.18007400
F	-7.47354800	2.11812300	-1.04030600
F	-8.72639700	1.22608900	0.49510300
F	-6.68521600	-0.60437500	1.04076300
F	-6.30786300	1.51222600	1.48020700
F	-5.37315900	-0.05345500	-1.51644200
F	-4.72622500	1.82039700	-0.57977300
F	-4.21023800	-1.54140100	0.28311300
F	-3.96793200	0.12225500	1.69225600
F	-2.64225100	0.12306400	-1.55496500
F	-2.16196700	1.30589700	0.22916000
F	-1.66746600	-2.07692000	-0.55653100
F	-1.63337700	-1.29535500	1.49054600
F	0.07734900	0.21008400	-1.18032100
F	0.27928500	0.39222900	1.00071400
F	0.91186200	-2.34082200	-1.13410700

F	0.88261100	-2.38415000	1.05274400
I	3.16526600	-0.78717900	-0.00363800
C	6.72971700	-0.75656700	-0.27961300
C	8.05182700	-0.36751500	-0.30110200
C	8.39241800	0.97591500	-0.01295700
C	7.30739600	1.83531000	0.28372200
C	6.02151200	1.33917200	0.27707400
N	5.71108800	0.06574200	0.00239800
H	6.46697600	-1.78399500	-0.50052500
H	8.80348300	-1.10200200	-0.53999900
H	7.45908600	2.87650200	0.51702800
H	5.18982500	1.99520700	0.50373400
N	9.67668600	1.41218500	-0.02081300
C	9.98013100	2.80595900	0.27273500
H	11.05274800	2.95697500	0.21293500
H	9.50135700	3.47823500	-0.44322200
H	9.65304600	3.08272200	1.27774400
C	10.76239500	0.49307000	-0.33276100
H	11.70589300	1.02600100	-0.28091600
H	10.80046400	-0.33534600	0.37842200
H	10.65953600	0.07949700	-1.33889400

$C_8F_{17}I$···2-Methylpyridine

C	-7.11419000	-0.62495100	0.47738300
C	-5.82986900	-0.50341100	-0.40849700
C	-4.50530900	-0.34458900	0.41444300
C	-3.30527800	0.18698500	-0.44784500
C	-1.90758500	-0.06664100	0.22323200
C	-0.76505600	0.82809700	-0.38040600
C	0.67651700	0.28965000	-0.06088700
C	1.81914500	1.32678600	-0.26959900
F	-7.40438700	0.53962300	1.05747300
F	-6.94336600	-1.55429200	1.42198500
F	-8.14009700	-0.98209500	-0.29922600
F	-5.99054300	0.56368500	-1.21743300
F	-5.74378400	-1.61561000	-1.16151500
F	-4.72232200	0.51784600	1.42740700
F	-4.18261800	-1.54788700	0.92990100
F	-3.47213600	1.51121700	-0.63457700
F	-3.32430200	-0.42973000	-1.64632800
F	-2.00514300	0.19577500	1.54254600
F	-1.58947300	-1.36588200	0.05920400
F	-0.90412600	2.07354400	0.11479600
F	-0.91530400	0.87699700	-1.72106000
F	0.70828700	-0.12369800	1.22660000
F	0.89815900	-0.77297300	-0.86413900
F	1.68542900	2.30947400	0.65272000
F	1.65691300	1.89255200	-1.49288900
I	3.83255600	0.43442500	-0.10197300

C	7.29783500	-0.44109800	0.94452700
C	8.55758000	-1.03780800	0.89110400
C	8.88160800	-1.86103700	-0.17778800
C	7.93891600	-2.07309200	-1.17536100
C	6.70844700	-1.44539400	-1.05255600
N	6.39529400	-0.65330100	-0.02510500
H	9.85478500	-2.32955500	-0.23091500
H	9.27090700	-0.85438500	1.68193900
H	8.14598900	-2.70567500	-2.02599500
H	5.93920200	-1.57845100	-1.80341100
C	6.89186200	0.45785000	2.07527700
H	6.62821700	1.44669000	1.69791800
H	6.00917400	0.06033500	2.57782300
H	7.69078400	0.56461500	2.80561500

$C_8F_{17}I$ ···2,6-Dimethylpyridine

C	-7.27374400	0.59810900	-0.51402100
C	-5.98862000	0.50980400	0.37464200
C	-4.66623600	0.30511200	-0.44137600
C	-3.46625600	-0.18625800	0.44456900
C	-2.06914500	0.02903400	-0.24096900
C	-0.92828000	-0.83755800	0.40539700
C	0.51412000	-0.31889100	0.05812400
C	1.65336900	-1.34931200	0.31765100
F	-7.56988800	-0.58905500	-1.04299300
F	-7.10044000	1.48525300	-1.49790800
F	-8.29691700	0.99277500	0.24795300
F	-6.15382700	-0.51786100	1.23225600
F	-5.89523100	1.65521800	1.07523400
F	-4.88805600	-0.60700600	-1.40873800
F	-4.34039800	1.47953000	-1.01781600
F	-3.63669600	-1.49886000	0.69806100
F	-3.48070200	0.49000900	1.61055800
F	-2.16929900	-0.29948900	-1.54513800
F	-1.74707400	1.33392500	-0.14317800
F	-1.07064300	-2.10566600	-0.02728500
F	-1.07679900	-0.81945500	1.74686500
F	0.54620500	0.02912800	-1.24825200
F	0.73922400	0.78159700	0.80707000
F	1.51853800	-2.37320600	-0.55714300
F	1.48960500	-1.85550600	1.56557700
I	3.66409400	-0.46815400	0.11217500
C	7.30319300	-0.17571200	-0.65213600
C	8.60210500	0.31060300	-0.78881800
C	8.88338500	1.60621200	-0.38337900
C	7.86467400	2.38270400	0.14701300
C	6.58588900	1.83985700	0.25815900
N	6.32458000	0.58449300	-0.13734900
H	9.88392800	2.00571700	-0.47945800

H	9.37307700	-0.32086300	-1.20624600
H	8.04995700	3.39616000	0.47256300
C	6.94515400	-1.57118600	-1.07563400
H	6.55164900	-2.13759500	-0.23083600
H	6.16595000	-1.55073800	-1.83839700
H	7.80942200	-2.09728200	-1.47507600
C	5.44778100	2.63859400	0.82542500
H	5.77084400	3.63639300	1.11410000
H	4.64339000	2.73013200	0.09469900
H	5.02999900	2.14012500	1.70081200

$C_8F_{17}I$ ···4-Methoxypyridine

C	7.48287400	-0.89883900	-0.17780000
C	6.19537500	-0.47892400	0.60628600
C	4.88462600	-0.53291700	-0.25166700
C	3.69541900	0.26622200	0.39104600
C	2.29596100	-0.13266100	-0.20149600
C	1.17965000	0.93469800	0.08824400
C	-0.27720100	0.37292700	-0.09100100
C	-1.38180900	1.46160200	-0.22609000
F	7.81627800	0.02665700	-1.07739500
F	7.29038500	-2.06529700	-0.80010500
F	8.48950300	-1.03281200	0.68944300
F	6.38584400	0.77569300	1.06320300
F	6.06394400	-1.31243400	1.65485500
F	5.14020100	-0.01744900	-1.47071400
F	4.52628300	-1.82492700	-0.39255700
F	3.90650900	1.58062000	0.18053800
F	3.68193400	0.03228500	1.71858200
F	2.41487800	-0.27707900	-1.53719100
F	1.93148700	-1.31423800	0.33441000
F	1.37022900	1.97550800	-0.74612000
F	1.31635600	1.36926000	1.35917100
F	-0.30868400	-0.40322600	-1.19854400
F	-0.54846300	-0.39609700	0.98563800
F	-1.20034100	2.11617400	-1.39884800
F	-1.20707200	2.36478100	0.77364900
I	-3.43100300	0.63349200	-0.15686600
C	-6.55178900	-0.90841500	-1.14168200
C	-7.85380700	-1.36256700	-1.16291300
C	-8.66092900	-1.14864300	-0.04000500
C	-8.10762900	-0.48338700	1.05650900
C	-6.78652300	-0.06836700	0.97290700
N	-6.01334500	-0.26824500	-0.09432600
H	-5.90505000	-1.05918600	-1.99659500
H	-8.25334300	-1.87458600	-2.02576600
H	-8.67022200	-0.28531400	1.95439100
H	-6.33051900	0.45067000	1.80678800
O	-9.92278000	-1.60741400	-0.10833500

C	-10.79264700	-1.40990000	1.01318300
H	-10.93069600	-0.34695800	1.21576700
H	-11.74132400	-1.85432700	0.73031400
H	-10.40289000	-1.91013800	1.90069900

$C_8F_{17}I$ ···3,5-Dimethylpyridine

C	7.39422300	0.68413500	0.54609600
C	6.11618000	0.57625000	-0.35057100
C	4.79264700	0.34449700	0.45627500
C	3.60632800	-0.16186300	-0.43972200
C	2.20176400	0.02436100	0.23892500
C	1.07872200	-0.85446500	-0.42179400
C	-0.37422100	-0.36550700	-0.07615300
C	-1.49590700	-1.40892000	-0.35284100
F	7.70751100	-0.49906000	1.07408800
F	7.20006000	1.56584900	1.53096100
F	8.41504700	1.09800800	-0.20889600
F	6.30508800	-0.44474800	-1.21128900
F	6.00666700	1.72278700	-1.04702500
F	5.02438900	-0.56928500	1.41976800
F	4.44378100	1.50993900	1.03746400
F	3.80126500	-1.46958800	-0.70070200
F	3.61602500	0.52227000	-1.60122900
F	2.30029800	-0.31412900	1.54076000
F	1.85920800	1.32478400	0.15092200
F	1.24142200	-2.12463300	-0.00210700
F	1.23398000	-0.81933500	-1.76236100
F	-0.41749800	-0.03269700	1.23434300
F	-0.61415300	0.74066300	-0.81320500
F	-1.34650200	-2.44278500	0.51047100
F	-1.31520500	-1.89890500	-1.60688700
I	-3.53119000	-0.56794200	-0.14538900
C	-7.04312000	-0.40451000	0.63718400
C	-8.36497900	0.01099100	0.78103700
C	-8.67815400	1.28871500	0.32426200
C	-7.70440600	2.10347900	-0.24785500
C	-6.41647000	1.58020800	-0.33799300
N	-6.09633700	0.35991600	0.09280900
H	-9.69443200	1.65439300	0.41523900
H	-6.73406400	-1.38768400	0.97173000
H	-5.61286800	2.16306100	-0.77227800
C	-9.40296900	-0.88043900	1.40296300
H	-9.81999800	-0.42212000	2.30114900
H	-10.23044600	-1.05344000	0.71325200
H	-8.98438400	-1.84677800	1.67953700
C	-8.02604900	3.48381000	-0.74786000
H	-7.14157100	3.98069300	-1.14312300
H	-8.77520100	3.44459800	-1.54025900
H	-8.43361700	4.10214100	0.05330100

$C_8F_{17}I$ Ph-I···Pyr

C	-6.94168900	0.66546200	-0.37476600
C	-5.65568900	0.40851400	0.47912100
C	-4.33019700	0.39633200	-0.35734700
C	-3.12575200	-0.25984300	0.40739700
C	-1.73070500	0.10840300	-0.21449100
C	-0.58171600	-0.86553900	0.23405900
C	0.85622300	-0.27580900	0.00027900
C	2.00425900	-1.32723900	0.03683400
F	-7.22170400	-0.38738400	-1.14299200
F	-6.78085500	1.74236100	-1.14883700
F	-7.97006200	0.87635400	0.45067300
F	-5.80884700	-0.77850800	1.10090400
F	-5.57687300	1.38209300	1.40505200
F	-4.54092700	-0.29055600	-1.49778300
F	-4.01649900	1.67013600	-0.66854300
F	-3.28377900	-1.59787500	0.37763300
F	-3.14720600	0.15464900	1.68988300
F	-1.82913800	0.06396600	-1.55879200
F	-1.42005700	1.36545600	0.15898400
F	-0.71692900	-2.01607700	-0.45412700
F	-0.72666900	-1.12956900	1.54994800
F	0.88114000	0.33790700	-1.20476900
F	1.07652200	0.64608700	0.96221400
F	1.87094900	-2.15074000	-1.03018100
F	1.84948200	-2.08214700	1.15450700
I	4.01360500	-0.41010900	0.00617000
C	7.51573500	0.07434900	-0.81036100
C	8.81433300	0.56229600	-0.86784700
C	9.14377400	1.66852900	-0.09456600
C	8.16401400	2.24349100	0.70526000
C	6.89150100	1.68812400	0.69991900
N	6.57229400	0.62505300	-0.04244000
H	10.14540400	2.07497200	-0.11485800
H	7.21662800	-0.78534000	-1.39661000
H	9.54455100	0.08372500	-1.50414200
H	8.37606300	3.10447000	1.32243700
H	6.09875800	2.10480400	1.30831800

$C_8F_{17}I$ ···3-Bromopyridine

C	-7.78156300	1.03135800	-0.53113100
C	-6.47733800	0.97048300	0.33157200
C	-5.21901300	0.46694700	-0.45595300
C	-4.04569800	0.01422300	0.48418100
C	-2.66251700	-0.06762600	-0.25673800
C	-1.60017100	-0.92702300	0.51966200
C	-0.12460100	-0.65968300	0.04800600
C	0.89866100	-1.75503100	0.47314300

F	-8.22362600	-0.19421900	-0.81327100
F	-7.55626000	1.68926700	-1.67185500
F	-8.72460800	1.67610400	0.16042500
F	-6.71552600	0.15042500	1.37553200
F	-6.23347700	2.21045400	0.79467100
F	-5.57749800	-0.57720700	-1.22933100
F	-4.78968200	1.46871900	-1.24950800
F	-4.35004400	-1.19836100	0.98740300
F	-3.93763100	0.89472300	1.49903400
F	-2.85262600	-0.61832000	-1.47292600
F	-2.19190500	1.18525900	-0.41385100
F	-1.90381600	-2.22777300	0.34246000
F	-1.68641900	-0.63818400	1.83528100
F	-0.11048500	-0.56689500	-1.30078500
F	0.25936400	0.52457300	0.56982100
F	0.60902000	-2.89931000	-0.18801600
F	0.73482500	-1.99586300	1.79750100
I	2.98588100	-1.18339300	0.05719100
C	6.46843200	-1.43544700	-1.07238200
C	7.80651900	-1.17179800	-1.33059000
C	8.34604700	0.03708900	-0.91168000
C	7.51251300	0.92727900	-0.25075200
C	6.18240900	0.59406700	-0.02791200
N	5.67780300	-0.56963300	-0.43528900
H	9.38269100	0.27867300	-1.09362900
H	6.01194900	-2.36603700	-1.38286900
H	8.41595000	-1.89742000	-1.84906700
H	5.51316700	1.27146600	0.48499100
Br	8.17461100	2.60917200	0.35819100

$C_8F_{17}I$ ···3-Chloropyridine

C	-7.33519100	0.90662600	-0.44743400
C	-6.04907300	0.71782500	0.42396400
C	-4.75172900	0.44156700	-0.41080700
C	-3.57535600	-0.13821900	0.45350500
C	-2.17538000	-0.00199300	-0.24628500
C	-1.08306200	-0.94561600	0.37563000
C	0.38436800	-0.51569700	0.01172000
C	1.45885100	-1.61947100	0.24593100
F	-7.71229700	-0.24799200	-0.99659900
F	-7.11614300	1.80134500	-1.41512000
F	-8.32281300	1.34951300	0.33472500
F	-6.27248100	-0.31254300	1.26505600
F	-5.87298700	1.84161700	1.14337700
F	-5.04224400	-0.43995400	-1.38823600
F	-4.35802700	1.60143400	-0.97422800
F	-3.82552100	-1.44109100	0.69032900
F	-3.53378500	0.51996300	1.62907400
F	-2.31139500	-0.30772500	-1.55268700

F	-1.77054700	1.27869700	-0.13738000
F	-1.31082000	-2.19786500	-0.06641300
F	-1.21103200	-0.93136500	1.71915500
F	0.42065300	-0.15848800	-1.29180700
F	0.69040200	0.56059700	0.76676900
F	1.24807500	-2.62388400	-0.63559900
F	1.28165600	-2.12565700	1.49139900
I	3.51782100	-0.86852200	0.01664500
C	7.10108600	-0.84152500	-0.77681100
C	8.42807600	-0.46420500	-0.93193800
C	8.81492700	0.80970700	-0.53945500
C	7.84638400	1.64777400	-0.00756500
C	6.53712500	1.20051500	0.11756500
N	6.17945700	-0.02447400	-0.26302800
H	9.83645000	1.14468300	-0.64204700
H	6.76198300	-1.82588500	-1.07120100
H	9.14565400	-1.15368100	-1.35184100
H	5.76845600	1.83999800	0.53020800
Cl	8.25613700	3.26557300	0.50879000

$C_8F_{17}I$ ···3,5-Dichloropyridine

C	7.81768200	0.66277000	0.72272700
C	6.54497400	0.70690500	-0.18664300
C	5.22632100	0.27805900	0.54352800
C	4.05646500	-0.06024400	-0.44840300
C	2.64508700	-0.04953700	0.24160500
C	1.54763600	-0.80313500	-0.59409900
C	0.08175500	-0.42206600	-0.17283400
C	-1.01003700	-1.42052800	-0.66384900
F	8.15950100	-0.59467400	1.00360900
F	7.59962700	1.32281400	1.86351100
F	8.82908200	1.24488900	0.07340900
F	6.76457600	-0.10973800	-1.23738800
F	6.40443200	1.96881300	-0.63307300
F	5.47889600	-0.81060700	1.29702500
F	4.84459500	1.28731900	1.35177800
F	4.28412700	-1.28144400	-0.97081800
F	4.05303800	0.84792300	-1.44435900
F	2.74567400	-0.64232100	1.44858000
F	2.26925200	1.23297200	0.41484400
F	1.73544400	-2.12762500	-0.43452800
F	1.70879600	-0.49766500	-1.89868600
F	0.02021500	-0.35620000	1.17577100
F	-0.18151300	0.79996900	-0.68127700
F	-0.84579900	-2.59671700	-0.01876900
F	-0.81512200	-1.64676400	-1.98522700
I	-3.05224100	-0.68080600	-0.31714900
C	-6.64289500	-0.64387400	0.59639500
C	-7.95916100	-0.24365600	0.79312100
C	-8.35274700	1.04117600	0.45187400

C	-7.38672000	1.88046700	-0.08154200
C	-6.08599400	1.42297700	-0.25487700
N	-5.73675100	0.18383800	0.08197000
H	-9.36890700	1.37434300	0.59542000
H	-6.31919900	-1.64192000	0.85689000
H	-5.32053200	2.06396100	-0.66958100
Cl	-7.79755700	3.51174500	-0.53746900
Cl	-9.11143600	-1.36262300	1.46986700

$C_6F_5I\cdots NEt_2H$

I	0.88934200	-0.05671600	-0.12273500
C	-1.26238500	-0.01375800	-0.03992100
C	-1.95489400	1.18073100	0.09439200
C	-2.01183300	-1.17853200	-0.11751000
C	-3.34021600	1.22297400	0.15076900
C	-3.39786900	-1.16362300	-0.06391800
C	-4.06544000	0.04402400	0.07115200
F	-1.41155100	-2.37283500	-0.24856400
F	-4.09649700	-2.30289900	-0.14109900
F	-5.39942900	0.07155000	0.12416500
F	-3.98301500	2.38988800	0.28150800
F	-1.29759800	2.34912300	0.17556800
N	3.63927400	-0.07821100	-0.20323000
H	3.81955500	-0.23925600	-1.18764200
C	4.09512100	-1.23405700	0.58254200
H	3.74987500	-1.08426000	1.60746300
H	3.56231000	-2.10607200	0.20020700
C	4.16710400	1.24130100	0.19293900
H	3.36487500	1.97522000	0.08879700
H	4.41052900	1.19725000	1.25540000
C	5.37390800	1.71308000	-0.61521900
H	6.22491100	1.04280700	-0.50614800
H	5.12252200	1.77226100	-1.67617600
H	5.67862500	2.70948700	-0.29141400
C	5.59857200	-1.50758500	0.57524500
H	5.96761800	-1.63720600	-0.44346300
H	6.15753000	-0.69634000	1.04111800
H	5.81202100	-2.42122100	1.13238000

$C_6F_5I\cdots NBuH_2$

I	0.57766200	-0.03366700	-0.57417500
C	-1.52496500	-0.00550100	-0.12460900
C	-2.24238600	-1.18063700	0.04674800
C	-2.21900300	1.18850400	0.00837200
C	-3.59828200	-1.17617200	0.33926200
C	-3.57463500	1.22024300	0.30049600
C	-4.26785200	0.03113600	0.46703500
F	-1.59202800	2.36637800	-0.14311600
F	-4.21910600	2.38695200	0.42349300

F	-5.57295000	0.04850600	0.74864100
F	-4.26564600	-2.32536200	0.49948400
F	-1.63884300	-2.37492300	-0.06642900
N	3.28720000	-0.06603100	-1.08527800
H	3.50331300	-0.91129400	-1.60188700
H	3.50591000	0.71475300	-1.69400900
C	4.07300600	0.00314600	0.15585400
H	3.77141100	0.90897000	0.68575700
H	3.77263700	-0.83936200	0.78216400
C	5.58765600	-0.00644200	-0.03923800
H	5.87145900	0.83804400	-0.67556500
H	5.87289700	-0.91444000	-0.58030600
C	6.35606900	0.06581200	1.28031100
H	6.06122900	0.97111400	1.81896300
H	6.06276700	-0.77650600	1.91366400
C	7.87129100	0.05651000	1.08611800
H	8.19328400	0.90680400	0.48136700
H	8.39583400	0.10834900	2.04112100
H	8.19454800	-0.85339300	0.57622400

C_6F_5I···Piperidine

I	-0.65111500	-0.17935300	-0.20353400
C	1.49702300	-0.03365900	-0.05224800
C	2.30279500	-1.16166300	-0.09839000
C	2.12861600	1.19236700	0.09534600
C	3.68431700	-1.08164900	-0.00257000
C	3.50803400	1.30063700	0.19346400
C	4.29004700	0.15683100	0.14424200
F	1.41486500	2.32936600	0.14883000
F	4.09126400	2.49743400	0.33519400
F	5.61920000	0.24753500	0.23763100
F	4.43789000	-2.18729300	-0.05029300
F	1.76354700	-2.38416000	-0.23933100
C	-3.91260600	-0.87022800	0.90213200
C	-5.44195400	-0.91506400	0.91636400
C	-6.03011000	0.46207900	0.59467300
C	-5.45583600	1.00199600	-0.71856700
C	-3.92611600	0.99368000	-0.68692500
H	-7.11872100	0.40923200	0.54204900
H	-5.78389100	-1.64232200	0.17343000
H	-5.78733600	-1.26729000	1.89056200
H	-3.55264700	-0.23266200	1.71413500
H	-3.48663800	-1.86134800	1.05749600
H	-5.79871400	0.38038800	-1.55160100
H	-5.81100700	2.01668400	-0.91003400
H	-3.50910700	1.30747300	-1.64374700
H	-3.56680100	1.69757200	0.06858200
H	-5.78581900	1.15561900	1.40576900
N	-3.36338800	-0.32597300	-0.35182700

H	-3.55265800	-0.97372800	-1.10994000

$C_6F_5I \cdots N(sec\text{-}Bu)H_2$

I	0.88136100	-0.14482100	-0.57688100
C	-1.22308500	-0.02527200	-0.16279500
C	-1.97124700	-1.16214600	0.10734300
C	-1.88850400	1.19224000	-0.15252400
C	-3.33016600	-1.09793900	0.37808200
C	-3.24644400	1.28309100	0.11540800
C	-3.97098100	0.13154100	0.38233200
F	-1.23051400	2.33517100	-0.40464200
F	-3.86321400	2.47096100	0.11888000
F	-5.27857300	0.20616700	0.64183400
F	-4.02797100	-2.21083500	0.63502700
F	-1.39614700	-2.37541300	0.11516100
N	3.60220800	-0.30352000	-1.19550300
H	3.69739000	-1.15915800	-1.73097800
H	3.77041500	0.46425600	-1.83612400
C	4.57232200	-0.27526200	-0.07954800
C	4.32800700	-1.48668000	0.81423500
H	4.39048300	-2.41215400	0.23904800
H	5.07022000	-1.54197100	1.60884800
H	3.33711200	-1.43406900	1.26778700
C	4.43295200	1.05715400	0.66134800
H	4.49588400	1.86667000	-0.07190700
H	3.43241100	1.11190200	1.09745300
C	5.49044600	1.28005600	1.74078200
H	5.38938300	2.27304900	2.17932000
H	5.40278500	0.55398800	2.54924400
H	6.49792900	1.19957200	1.32723000
H	5.59638200	-0.33704800	-0.47101500

$C_6F_5I \cdots$Pyrrolidine

I	1.03536400	-0.42232200	-0.08493300
C	-1.09516600	-0.10120600	-0.02717000
C	-1.63177900	1.17788700	-0.02374700
C	-1.98526300	-1.16440800	0.00833000
C	-3.00068500	1.39902100	0.01336700
C	-3.35829000	-0.97090800	0.04594900
C	-3.86853300	0.31826000	0.04836100
F	-1.53990400	-2.43169700	0.00741300
F	-4.19458400	-2.01572400	0.07991900
F	-5.18844200	0.51786100	0.08436100
F	-3.49163900	2.64446500	0.01568700
F	-0.83225900	2.25687400	-0.05672600
C	4.38260500	0.03437600	-1.16763200
C	4.43692400	1.07722300	1.04699800
H	5.39239500	-0.32000300	-1.40523800
C	4.37195300	-0.45633900	1.12109800

H	3.56376900	1.51612100	1.52670000
H	3.77797100	-0.82333200	1.95676500
C	4.44036900	1.40284400	-0.47155600
H	3.56630800	1.99657900	-0.73357400
H	5.32245600	1.96296200	-0.77526300
N	3.74526600	-0.82894300	-0.15875100
H	3.79804000	0.04169400	-2.08636100
H	5.31936100	1.45916600	1.55637400
H	5.37922400	-0.88103500	1.20256300
H	3.85798300	-1.81300800	-0.36915600

$C_6F_5I\cdots N(iso\text{-}Pr)H_2$

I	-0.65540500	0.06908000	-0.12550100
C	1.48697400	0.02420400	-0.03908200
C	2.17163900	-1.14965000	0.24127900
C	2.24228300	1.16698300	-0.25983100
C	3.55673200	-1.19207300	0.30207000
C	3.62816900	1.14983200	-0.20476800
C	4.28869400	-0.03609900	0.07760900
F	1.64830900	2.33854800	-0.53667000
F	4.33299800	2.26663700	-0.42166400
F	5.62229500	-0.06480100	0.13311300
F	4.19243800	-2.33769100	0.57486800
F	1.50690300	-2.29413800	0.46524900
N	-3.50742000	0.17894100	-0.36001100
H	-3.48984600	0.68567600	-1.23691800
C	-4.20123000	1.03164500	0.63950100
C	-4.13847500	-1.13199900	-0.63435300
H	-5.23972200	0.68708700	0.72138900
H	-5.21367600	-0.97477300	-0.80481000
C	-4.21631100	2.47643700	0.14538900
H	-4.70098500	3.12602100	0.87387900
H	-3.19592800	2.83738300	-0.00097300
H	-4.75369700	2.56720400	-0.80021700
C	-3.55886800	0.94826200	2.02500600
H	-3.44703500	-0.07919000	2.36263000
H	-2.57023400	1.40760900	2.01613300
H	-4.17468000	1.48070800	2.75138700
C	-3.54197700	-1.72299500	-1.90886700
H	-4.06232300	-2.64349500	-2.17293700
H	-3.63580200	-1.03191400	-2.74893600
H	-2.48537600	-1.95499900	-1.77430200
C	-3.98723500	-2.09294600	0.54138800
H	-2.93421300	-2.22496400	0.79425700
H	-4.51271800	-1.73716500	1.42590200
H	-4.40418400	-3.06562700	0.27903500

$C_6F_5I\cdots NEt(iso\text{-}Pr)H$

I	0.85352300	0.02817700	-0.17472500

C	-1.29218400	0.00276100	-0.05572700
C	-2.01468800	1.16124700	0.19043500
C	-2.00937100	-1.17358200	-0.21872800
C	-3.39931500	1.15715300	0.27328000
C	-3.39395500	-1.20393000	-0.14099900
C	-4.09242700	-0.03204400	0.10647100
F	-1.37781600	-2.33382600	-0.45932300
F	-4.06144500	-2.35282200	-0.30224000
F	-5.42527600	-0.04864000	0.18370100
F	-4.07195900	2.28942000	0.51208900
F	-1.38852100	2.33707900	0.35860100
N	3.66307100	0.12771300	-0.33138100
H	3.81452400	-0.03867300	-1.32073800
C	4.34597600	-0.94551000	0.42210200
C	4.11974400	1.49009200	-0.01510700
H	5.43293800	-0.80724400	0.32443900
H	5.21109500	1.51001700	0.10651200
C	3.98024300	-2.29699800	-0.18304900
H	4.52696200	-3.09501000	0.31896500
H	2.91280200	-2.49270200	-0.07585300
H	4.22991200	-2.33558100	-1.24514500
C	3.99710600	-0.88732000	1.90715600
H	4.29657300	0.05748200	2.35911900
H	2.92403100	-1.01365200	2.05511200
H	4.51319600	-1.68722300	2.43827900
C	3.70605200	2.47930900	-1.09450500
H	4.04615400	3.48222400	-0.83720400
H	4.14575400	2.21353100	-2.05819400
H	2.62286700	2.50589300	-1.20987800
H	3.68706000	1.78557200	0.94125100

C_6F_5I···N(*tert*-Bu)

I	-0.88631800	-0.06217300	-0.39939800
C	1.24415800	-0.01569300	-0.12800400
C	1.93512200	1.18480400	-0.04672900
C	1.98445400	-1.18468100	-0.02487200
C	3.31017200	1.22850200	0.13048500
C	3.36007600	-1.16781700	0.15255700
C	4.02630800	0.04556200	0.23092100
F	1.38493800	-2.38410900	-0.09432300
F	4.04964400	-2.31090800	0.24909400
F	5.35016500	0.07470100	0.40200500
F	3.95152000	2.40090300	0.20537400
F	1.28628800	2.35675500	-0.13829800
N	-3.64165100	-0.13189500	-0.82411600
H	-3.81273800	0.58721300	-1.51958000
H	-3.80985800	-1.02192300	-1.28199200
C	-4.56409800	0.03653300	0.32790600
C	-4.27793100	1.40121400	0.95877300

H	-4.45107100	2.20511600	0.24052900
H	-4.93227800	1.56808200	1.81429000
H	-3.24396600	1.46244900	1.29716400
C	-4.27196700	-1.08561200	1.32675500
H	-4.43988900	-2.06382300	0.87176700
H	-3.23819200	-1.04080500	1.66833700
H	-4.92673000	-1.00120100	2.19397300
C	-6.02824900	-0.03503100	-0.13127100
H	-6.70740800	0.08815500	0.71381900
H	-6.24367400	0.75125700	-0.85704000
H	-6.23904200	-0.99883600	-0.59817000

$C_6F_5I\cdots NBu_2H$

I	0.24035800	-0.01390600	-0.24378400
C	-1.90262300	0.00204300	-0.06454600
C	-2.59755000	1.17822700	0.17673800
C	-2.64511800	-1.16369200	-0.18432100
C	-3.97932900	1.20158400	0.29586900
C	-4.02747300	-1.16703700	-0.06962400
C	-4.69792800	0.02232400	0.17195600
F	-2.04122300	-2.34019700	-0.41777500
F	-4.71965700	-2.30643500	-0.18958700
F	-6.02839600	0.03207600	0.28453100
F	-4.62505700	2.35065900	0.52915300
F	-1.94615300	2.34557800	0.30453300
N	3.02155100	-0.02627100	-0.30732700
H	3.32630800	-0.08327700	-1.27361200
C	3.48744400	-1.21573200	0.41818700
H	2.97677000	-1.23748200	1.38504100
H	4.56133200	-1.13722900	0.62701900
C	3.49597200	1.23552600	0.27664100
H	2.98728400	1.37086300	1.23521500
H	4.56980600	1.17479600	0.49159900
C	3.19294400	-2.49827000	-0.35143800
H	2.12987800	-2.52310400	-0.59756500
H	3.73509800	-2.47637800	-1.30310000
C	3.20787200	2.42364000	-0.63404000
H	3.74987300	2.29110600	-1.57674100
H	2.14489000	2.42576000	-0.88176800
C	3.58342300	3.77588700	-0.01996500
H	3.22519400	4.56547100	-0.68439000
H	3.04264400	3.90275300	0.92249200
C	5.08226400	3.96780600	0.21839600
H	5.29372100	4.97397500	0.58274400
H	5.47061600	3.26455900	0.95586000
H	5.64463500	3.82297000	-0.70683600
C	3.56209000	-3.77386500	0.41229000
H	3.02234900	-3.78949300	1.36372700
H	3.19827400	-4.63193500	-0.15741800

C	5.06026300	-3.94605000	0.66922700
H	5.45406800	-3.16556700	1.32113100
H	5.26668700	-4.90531300	1.14572300
H	5.62163500	-3.91104400	-0.26719700

$C_6F_5I \cdots NPr_2H$

I	0.67318300	-0.02231700	-0.19047700
C	-1.47240400	-0.00095900	-0.05943700
C	-2.16808300	1.17268000	0.19211000
C	-2.21631900	-1.16073600	-0.22121900
C	-3.55206500	1.19915900	0.28129200
C	-3.60087600	-1.16067000	-0.13722700
C	-4.27210500	0.02593300	0.11550200
F	-1.61176600	-2.33430400	-0.46696800
F	-4.29441700	-2.29421300	-0.29752000
F	-5.60465900	0.03877400	0.19876000
F	-4.19843000	2.34554100	0.52549200
F	-1.51532800	2.33412400	0.35973200
N	3.46018300	-0.03392500	-0.19883000
H	3.78087200	-0.14203400	-1.15557800
C	3.91412200	-1.18132500	0.59643000
H	3.40675700	-1.13720300	1.56395600
H	4.99199300	-1.11027800	0.79979900
C	3.92396800	1.25670100	0.32483400
H	3.41778200	1.43010000	1.27831300
H	5.00159300	1.22382300	0.53899700
C	3.61526800	-2.50982800	-0.08651700
H	2.54376600	-2.58384500	-0.27977200
H	4.11192700	-2.52915800	-1.06136100
C	3.63400200	2.40509800	-0.63315100
H	4.12963800	2.20665300	-1.58830800
H	2.56279700	2.44276700	-0.83825400
C	4.10446900	3.74817600	-0.07864800
H	3.89453900	4.55707000	-0.77863100
H	3.60028100	3.98106900	0.86119000
C	4.07630900	-3.70189100	0.74960500
H	3.57140100	-3.71905500	1.71737400
H	3.85971600	-4.64312500	0.24412600
H	5.15132700	-3.66198700	0.93568300
H	5.17929500	3.74215000	0.11265300

$C_6F_5I \cdots N(iso\text{-}Pr)_2H$

I	0.15419100	0.15659100	-0.04317400
C	-1.96485500	0.04518700	-0.01045300
C	-2.61653200	-1.18041200	0.02352500
C	-2.74642100	1.19272500	-0.02214900
C	-4.00066500	-1.26639700	0.04552900
C	-4.13179100	1.12763500	-0.00059700
C	-4.76155800	-0.10725400	0.03349800

F	-2.18128700	2.40720700	-0.05481700
F	-4.86424700	2.24625700	-0.01249600
F	-6.09340600	-0.17986000	0.05446500
F	-4.60643300	-2.45799700	0.07823300
F	-1.92205000	-2.32633600	0.03636300
N	3.31421400	0.02647100	-0.00398700
C	3.85679300	1.30430000	-0.52780500
H	3.85846800	1.18696800	-1.61299600
C	3.50585900	-1.12934800	-0.91469900
H	2.94988500	-0.86313700	-1.81933700
C	2.94262500	2.49208200	-0.20927000
H	1.97165500	2.38777700	-0.68672500
H	2.78546000	2.59884800	0.86484300
C	2.87919900	-2.41628400	-0.37284000
H	3.44320400	-2.82595000	0.46417700
H	1.85110100	-2.26983500	-0.05188900
H	3.39608100	3.41727400	-0.56757200
H	2.87751400	-3.16694700	-1.16303600
C	5.29267100	1.65759600	-0.09752800
H	5.32636400	1.93158800	0.95766800
H	5.99197000	0.84244900	-0.26073500
H	5.63881900	2.51959900	-0.66961700
C	4.95389900	-1.41021900	-1.36002700
H	5.39109500	-0.56108000	-1.88170400
H	5.59654600	-1.66296600	-0.51819700
H	4.95802900	-2.25406300	-2.05214600
C	3.44981900	-0.19897400	1.44068100
H	2.63237000	-0.84926100	1.75604400
C	4.75384200	-0.78658200	2.00630700
H	5.62706400	-0.18380800	1.77323600
H	4.66551600	-0.84215800	3.09348500
H	4.93551500	-1.79606000	1.64348400
H	3.26119400	0.75838500	1.92636600

$C_6F_5I \cdots NEt_3$

I	-0.69299000	-0.04226900	0.00464400
C	1.44373000	-0.01198600	0.00482300
C	2.14549000	1.18481000	-0.03127400
C	2.18120000	-1.18706400	0.04149900
C	3.53212800	1.21795400	-0.03125300
C	3.56824600	-1.17814700	0.04237300
C	4.24688200	0.03042200	0.00581700
F	1.56882100	-2.38018500	0.07756100
F	4.25655900	-2.32485800	0.07824500
F	5.58143700	0.05053000	0.00635400
F	4.18529300	2.38505800	-0.06669400
F	1.49743200	2.35889300	-0.06792900
N	-3.61952400	-0.05102200	-0.28163500
C	-3.87996200	-1.26611400	-1.07914900

H	-3.30795200	-1.16852200	-2.00292200
C	-3.81526000	1.13621800	-1.13782900
H	-3.15609000	1.02085600	-1.99950100
C	-4.45521300	0.00331600	0.94220900
H	-5.28295900	0.70348000	0.78839600
H	-4.91723800	-0.96975100	1.09615800
H	-4.94139000	-1.30435600	-1.36874400
H	-4.84630300	1.14623000	-1.52448600
C	-3.52236000	2.47148600	-0.46584800
H	-3.63681800	3.27051000	-1.19890500
H	-4.20793700	2.67973700	0.35441400
H	-2.50462500	2.50923200	-0.08031300
C	-3.48815100	-2.57080800	-0.39652800
H	-4.05092400	-2.74777800	0.51933900
H	-3.69174400	-3.40369900	-1.07045300
H	-2.42776900	-2.58467800	-0.15018400
C	-3.67721500	0.37680000	2.19801200
H	-3.17991100	1.33933500	2.09244300
H	-4.35196000	0.43223400	3.05411000
H	-2.91421000	-0.37170200	2.41101600

C_6F_5I ···NBu_3

I	-0.29320700	-0.00000400	-0.10243200
C	-2.43422300	-0.00000100	0.11334300
C	-3.15144500	-1.18513700	0.18479600
C	-3.15144200	1.18513600	0.18480000
C	-4.53165300	-1.19867000	0.32235300
C	-4.53164900	1.19867200	0.32235700
C	-5.22511400	0.00000200	0.39167400
F	-2.52436500	2.37105000	0.12191200
F	-5.19992500	2.35660900	0.38887600
F	-6.55378000	0.00000400	0.52459800
F	-5.19993100	-2.35660500	0.38886800
F	-2.52437200	-2.37105300	0.12190500
N	2.47786600	-0.00000400	-0.45890400
C	2.77352100	1.20185400	-1.26363600
H	2.13649100	1.14504000	-2.14928900
C	2.77353300	-1.20186200	-1.26363000
H	2.13651300	-1.14505300	-2.14928900
C	3.09902400	0.00000200	0.87744200
H	2.72017900	0.86821600	1.41568800
H	2.72018500	-0.86821200	1.41569200
H	3.81063000	1.17248400	-1.62657400
H	3.81064600	-1.17249000	-1.62655700
C	2.52986700	-2.53353400	-0.56269900
H	3.26139700	-2.68147300	0.23492800
H	1.54521500	-2.52827300	-0.09043300
C	2.52985900	2.53352800	-0.56270900
H	3.26139800	2.68147300	0.23490900

H	1.54521200	2.52826800	-0.09043200
C	4.62887100	0.00000800	0.91947500
H	5.01747500	0.87581000	0.39356100
H	5.01748100	-0.87579900	0.39357400
C	5.15566700	0.00001900	2.35421500
H	4.76391200	0.87505600	2.88121600
H	4.76391400	-0.87501000	2.88123000
C	6.68140500	0.00002200	2.42640300
H	7.09593500	0.88137700	1.93259900
H	7.03207900	0.00003100	3.45936700
H	7.09593600	-0.88134100	1.93261400
C	2.61886100	-3.70851700	-1.53727300
H	1.86713400	-3.58308300	-2.32163200
C	2.61883900	3.70850700	-1.53728900
H	1.86710400	3.58306900	-2.32163800
H	3.59139300	3.69157800	-2.03806000
C	2.42061300	5.05788400	-0.84972200
H	3.18089600	5.22219100	-0.08327200
H	2.48169500	5.88111700	-1.56268800
H	1.44421200	5.10821200	-0.36349500
H	3.59142100	-3.69158700	-2.03803300
C	2.42063000	-5.05789000	-0.84970200
H	1.44422400	-5.10821900	-0.36348600
H	3.18090400	-5.22219300	-0.08324200
H	2.48172200	-5.88112800	-1.56266300

C_6F_5I ···NPr_3

I	0.67153400	-0.18672700	-0.09819800
C	2.76770600	-0.07138200	-0.04717400
C	3.54851800	-1.22048600	-0.02366100
C	3.41291000	1.15922800	-0.03425200
C	4.93327200	-1.14898600	0.01113600
C	4.79665700	1.24614700	0.00054000
C	5.55948800	0.08826600	0.02332500
F	2.71495000	2.29976300	-0.05470400
F	5.39845100	2.43857800	0.01238400
F	6.88960700	0.16438900	0.05698100
F	5.66687100	-2.26503000	0.03324700
F	2.98492000	-2.43316200	-0.03371700
N	-2.87912700	0.04752900	0.03800800
C	-2.93334000	1.42335200	-0.31834600
C	-2.12390800	2.34936600	0.34630600
C	-3.78744000	1.87279200	-1.32816500
C	-2.16888200	3.69345200	0.00544000
H	-1.46391800	2.01172800	1.13186800
C	-3.81705600	3.21813100	-1.67428400
H	-4.42340100	1.16800600	-1.84371900
C	-3.01105300	4.13717300	-1.01063500
H	-1.53283200	4.39460900	0.52875300

H	-4.48327300	3.54827300	-2.46001600
H	-3.03845000	5.18383800	-1.28007100
C	-2.95846300	-0.32180500	1.40777700
C	-2.27799200	-1.45344500	1.86883600
C	-3.70830700	0.43084500	2.31532300
C	-2.34602800	-1.81930700	3.20526000
H	-1.70188600	-2.04881100	1.17551100
C	-3.76052100	0.06659800	3.65515200
H	-4.24577700	1.30256900	1.97199700
C	-3.08271500	-1.05957000	4.10995000
H	-1.81029300	-2.69657900	3.54208400
H	-4.34481200	0.66301300	4.34300600
H	-3.12790600	-1.34214700	5.15248800
C	-3.05941800	-0.93984500	-0.96806900
C	-2.37015600	-0.83416700	-2.18054700
C	-3.91882600	-2.02395600	-0.77225400
C	-2.53554400	-1.79302700	-3.16951300
H	-1.71126300	0.00574800	-2.34704700
C	-4.06911900	-2.98760900	-1.76177200
H	-4.46488100	-2.11325500	0.15537600
C	-3.38144100	-2.87983600	-2.96604600
H	-1.99121900	-1.69477200	-4.09908300
H	-4.73761900	-3.82086500	-1.59139000
H	-3.50312400	-3.62995700	-3.73497700

C_6F_5I ···Pyridine-N-oxide

I	-0.55552400	-0.45954900	-0.67914600
C	-4.14777800	1.07799400	-0.38554800
C	-4.90627200	1.72600200	0.56650200
C	-5.47878400	1.00658800	1.60822500
C	-5.26312000	-0.36521500	1.65885900
C	-4.49790300	-0.98050800	0.69067600
N	-3.95071200	-0.26326800	-0.32284600
H	-6.07512600	1.50103300	2.36002900
H	-3.67121300	1.56371600	-1.22063900
H	-5.03972000	2.79395200	0.48050000
H	-5.68238800	-0.97164300	2.44766600
H	-4.28199900	-2.03551900	0.66177200
C	1.47943700	-0.10124200	-0.17995200
C	2.32217300	-1.13811100	0.19838200
C	2.01487600	1.17953300	-0.21669800
C	3.65053800	-0.91423300	0.52898700
C	3.34027700	1.42550700	0.10982200
C	4.16220700	0.37360200	0.48437400
F	1.25923300	2.22865300	-0.57193800
F	3.83146500	2.66892200	0.06619200
F	5.43888400	0.59949400	0.80019600
F	4.44132400	-1.93052300	0.89068700
F	1.87305800	-2.39978400	0.25723100

O	-3.22517500	-0.86161500	-1.22971700

C_6F_5I ···Trimethylamine-N-Oxide

I	0.91644300	0.34013200	-0.11091300
C	-1.21594800	0.08745100	-0.03586700
C	-1.79459500	-1.17368500	-0.02382600
C	-2.07309300	1.17819100	0.00228000
C	-3.16938200	-1.35121600	0.02337400
C	-3.45127300	1.02824000	0.04964600
C	-4.00261000	-0.24376100	0.06015700
F	-1.58858500	2.43066200	-0.00535200
F	-4.25369400	2.09934700	0.08564700
F	-5.32818500	-0.40143000	0.10582700
F	-3.69911400	-2.58094200	0.03340400
F	-1.03074800	-2.27863300	-0.05820700
O	3.49644100	0.80415200	-0.25799700
C	5.79790500	0.42707200	-0.14504700
H	6.56535700	-0.30147300	0.10786800
H	5.86245100	0.70890400	-1.19040700
H	5.87943200	1.30778000	0.48269100
C	4.28306900	-0.53733200	1.51873700
H	3.28509300	-0.94215300	1.64717600
H	5.03427200	-1.27402900	1.79489600
H	4.39450100	0.36762000	2.10598800
C	4.26273400	-1.36748900	-0.78811200
H	5.01541700	-2.11419800	-0.54452000
H	3.26644800	-1.75817500	-0.61214500
H	4.35738100	-1.04452900	-1.81915900
N	4.43982000	-0.15940100	0.07858100

C_6F_5I ···N.N-Dimethylimidazolidinone

I	0.27281600	-0.16136600	-0.58928200
C	-1.79415400	-0.06814000	-0.15108900
C	-2.53501600	-1.22099300	0.07679800
C	-2.45952800	1.14926000	-0.08032900
C	-3.89078900	-1.16894000	0.36484900
C	-3.81460900	1.22196300	0.20620600
C	-4.53361200	0.05779200	0.43001300
F	-1.80626900	2.30048100	-0.28807900
F	-4.43216100	2.40628900	0.26875500
F	-5.83721900	0.11769400	0.70683200
F	-4.58206700	-2.29300200	0.58032100
F	-1.95728900	-2.42847400	0.02493000
O	3.06928500	-0.33557500	-1.17050800
C	5.57179100	-0.22357100	1.28905700
C	4.97659300	1.18735200	1.35489300
H	6.54614100	-0.22448700	0.78763600
H	5.73502000	1.96778100	1.35677500
C	3.31569700	2.36114700	-0.14299400

H	2.78545100	2.17737900	-1.07320900
H	2.58312500	2.51914900	0.65523400
H	3.91863100	3.26249000	-0.24811800
C	4.80913500	-2.29086400	0.05406800
H	3.97228400	-2.60871200	-0.56166500
H	5.73302700	-2.37954400	-0.52758300
H	4.88058900	-2.94358600	0.92338800
C	3.86371600	-0.03929700	-0.27126400
N	4.17149000	1.23058200	0.14052900
N	4.58356600	-0.92852800	0.48219100
H	4.34189800	1.31347000	2.23913000
H	5.68459400	-0.68242900	2.26931300

C_6F_5I ···Triphenylphosphineoxide

I	1.26151000	-0.21377600	-0.77286800
C	3.34395300	-0.09110800	-0.40751800
C	3.94975300	1.12075100	-0.10108200
C	4.15448700	-1.21777000	-0.46255500
C	5.31212900	1.21385800	0.14128400
C	5.51883900	-1.14627400	-0.22337000
C	6.10069600	0.07513700	0.07974600
F	3.63755300	-2.42016200	-0.75132200
F	6.27723900	-2.24632100	-0.28299200
F	7.41248800	0.15435300	0.31130600
F	5.87079400	2.39335400	0.43392900
F	3.22869600	2.24850000	-0.02956300
O	-1.50178800	-0.31447500	-1.30032700
C	-4.17177800	-0.81508800	-0.78835000
C	-5.09099300	-1.31651300	0.13434900
C	-4.46634800	-0.85538300	-2.15319400
C	-6.29838700	-1.84751400	-0.30536700
H	-4.86635700	-1.30133700	1.19185400
C	-5.67265700	-1.38907900	-2.58829700
H	-3.74850400	-0.47836700	-2.86864800
C	-6.58996300	-1.88296800	-1.66493300
H	-7.00690200	-2.23714200	0.41259000
H	-5.89595200	-1.42190400	-3.64582900
H	-7.52824600	-2.29936700	-2.00538700
C	-2.17680400	-0.78288700	1.34027900
C	-1.48781700	-1.99855600	1.35410100
C	-2.52125000	-0.16955400	2.54499500
C	-1.15049600	-2.59377700	2.56213400
H	-1.20649300	-2.46760200	0.42151300
C	-2.18296600	-0.76959500	3.75316700
H	-3.04437400	0.77646600	2.54575200
C	-1.49910900	-1.98044200	3.76246200
H	-0.61283600	-3.53192600	2.56773600
H	-2.44907500	-0.28871000	4.68440300
H	-1.23361700	-2.44387400	4.70290000

C	-2.90392900	1.69928200	-0.01639400
C	-4.16668200	2.20007800	0.30448700
C	-1.81924100	2.57339900	-0.12315100
C	-4.34046800	3.56200800	0.52344200
H	-5.01552600	1.53455000	0.37781700
C	-1.99755900	3.93354400	0.09472100
H	-0.84221900	2.19031200	-0.38276500
C	-3.25720500	4.42841200	0.42018800
H	-5.32121900	3.94540000	0.76927500
H	-1.15535100	4.60625900	0.00838400
H	-3.39480100	5.48792100	0.58802900
P	-2.59216000	-0.07280000	-0.28147300

C_6F_5I ···Pyrrolidine-N-oxide

I	0.59286300	-0.61440300	0.08484600
C	-1.47043400	-0.12826400	0.02429300
C	-2.44109700	-1.11759900	-0.06459400
C	-1.89975700	1.19159100	0.07154800
C	-3.79281400	-0.80927000	-0.10577500
C	-3.24638100	1.52109200	0.03202200
C	-4.19738800	0.51605600	-0.05726900
F	-1.01692300	2.19687700	0.15704100
F	-3.63407400	2.80008000	0.07887500
F	-5.49511500	0.82275000	-0.09648300
F	-4.70825400	-1.78004200	-0.19176700
F	-2.09737900	-2.41168000	-0.11383700
O	3.34638200	-1.23270800	0.17728500
C	5.71999500	-0.60317100	0.16627000
C	6.33731900	0.69254400	-0.37831300
H	5.95143700	-0.75998900	1.22237900
C	5.23627300	1.74803800	-0.16043100
H	3.09482300	1.33357100	-0.16410300
H	7.26704500	0.96660600	0.11336500
H	5.20503800	2.49604400	-0.95005700
C	4.22267000	-0.37364000	0.05986800
N	4.02126300	0.93774600	-0.16390700
H	6.00980000	-1.50220700	-0.37204600
H	6.53591800	0.58859000	-1.44449500
H	5.34969200	2.26206200	0.79699300

C_6F_5I ···Dimethylsulfoxide

I	0.78400200	-0.17432500	-0.25819900
C	-1.32237000	-0.04012200	-0.07214900
C	-1.96114200	1.19067300	0.00902100
C	-2.11563400	-1.17955400	-0.02747500
C	-3.33928200	1.28949100	0.13008500
C	-3.49525100	-1.10226500	0.09318900
C	-4.11014600	0.13786600	0.17240600
F	-1.56682600	-2.39989100	-0.10000700

F	-4.23649800	-2.21463000	0.13419200
F	-5.43660100	0.22268700	0.28879400
F	-3.92995500	2.48697600	0.20588800
F	-1.25856300	2.33138900	-0.02796900
O	3.59017400	-0.37241400	-0.56064600
S	4.52252000	0.15541800	0.52964900
C	5.87110600	-1.05484600	0.64881300
H	6.63371600	-0.66415600	1.32010300
H	5.44254000	-1.96661600	1.05666400
H	6.27389900	-1.23035300	-0.34679000
C	5.46724100	1.50355700	-0.23773200
H	6.23797100	1.83143000	0.45744300
H	5.90144400	1.14266400	-1.16811000
H	4.76548100	2.31130400	-0.42898700

C_6F_5I ···Dimethylformamide

I	-0.85283200	-0.63663300	-0.15436600
C	1.19584200	-0.13785800	-0.03479200
C	2.17243700	-1.12205400	0.05362300
C	1.61351000	1.18710700	-0.04183600
C	3.51985700	-0.80266800	0.13292200
C	2.95614700	1.52577100	0.03646400
C	3.91360400	0.52675800	0.12447500
F	0.72439300	2.18572900	-0.12458100
F	3.33331800	2.80837200	0.02824600
F	5.20704800	0.84312800	0.20089800
F	4.44125600	-1.76747700	0.21746100
F	1.83925600	-2.41890500	0.06506500
O	-3.66658700	-1.35180300	-0.26085700
C	-4.55213900	-0.73400800	0.33483100
C	-5.90516600	1.20059500	0.94567300
H	-5.48263300	2.02692400	1.51957700
H	-6.68744100	1.59320400	0.29443600
H	-6.34673700	0.48496300	1.63512900
C	-4.17751300	1.38219300	-0.82264700
H	-3.53308900	0.75808500	-1.43219400
H	-4.90851100	1.88151200	-1.45933700
H	-3.57385800	2.14023400	-0.32096700
N	-4.86915300	0.55505600	0.15487200
H	-5.17780100	-1.22694100	1.09144900

C_6F_5I ···Acetophenone

I	0.13642600	-0.83807600	0.36634400
C	-1.84511600	-0.17863000	0.08449300
C	-2.87850900	-1.07964300	-0.14063500
C	-2.16047500	1.17414100	0.11837200
C	-4.18479000	-0.65177300	-0.32597600
C	-3.46105800	1.61951500	-0.06513000
C	-4.47743800	0.70303900	-0.28807300

F	-1.21141200	2.09414500	0.33083900
F	-3.74098100	2.92593600	-0.02840600
F	-5.73063200	1.12273200	-0.46553900
F	-5.16300800	-1.53669900	-0.54098100
F	-2.64232100	-2.39636700	-0.18574200
O	2.93525800	-1.83165900	0.63515400
C	3.89829300	-1.42102200	-0.00017200
C	4.19348100	0.03650800	-0.08155900
C	5.10642300	0.55548600	-1.00585500
C	3.53471100	0.91220000	0.78920600
C	5.34142400	1.92299200	-1.06818400
H	5.62858100	-0.09950700	-1.68763600
C	3.77835300	2.27521300	0.73398200
H	2.83987500	0.50956800	1.51087600
C	4.67951700	2.78386200	-0.19877500
H	6.04098200	2.31603100	-1.79280300
H	3.26847100	2.94248200	1.41503500
H	4.86692000	3.84803500	-0.24510600
C	4.80256500	-2.39249400	-0.71523400
H	5.84715000	-2.22345900	-0.45366600
H	4.51355200	-3.40757900	-0.45945300
H	4.71271300	-2.25833700	-1.79501600

C_6F_5I ···Benzophenone

I	0.55657300	-0.46928000	0.55889400
C	2.61745800	-0.32829100	0.12711900
C	3.37473600	-1.45868800	-0.15355500
C	3.26251700	0.90217200	0.11166600
C	4.72902200	-1.37169000	-0.44021300
C	4.61576100	1.00845700	-0.17288600
C	5.35225400	-0.13315400	-0.45009000
F	2.59134200	2.03119600	0.37288300
F	5.21461500	2.20363300	-0.18181900
F	6.65408200	-0.04029000	-0.72482400
F	5.43731600	-2.47360000	-0.70693300
F	2.81543600	-2.67525900	-0.15594100
O	-2.32353000	-0.78539700	1.03873700
C	-3.17586600	-0.14058400	0.43232600
C	-2.92392600	1.28644400	0.09021800
C	-3.35682600	1.84432000	-1.11610400
C	-2.17007000	2.06409200	0.97476100
C	-3.03204900	3.15695700	-1.43432400
H	-3.92447600	1.24637600	-1.81433100
C	-1.86966800	3.38191400	0.66704300
H	-1.83033100	1.62505700	1.90168300
C	-2.29614600	3.92876500	-0.54105100
H	-3.35355000	3.57662900	-2.37745300
H	-1.29876100	3.98155100	1.36234000
H	-2.05315400	4.95383800	-0.78567800

C	-4.45562700	-0.78987200	0.03914700
C	-4.46597000	-2.17345200	-0.17027200
C	-5.64981400	-0.07088900	-0.07267200
C	-5.64414300	-2.82200200	-0.50527400
H	-3.54108100	-2.72394200	-0.07431400
C	-6.83341100	-0.72651800	-0.38719500
H	-5.65855500	0.99412000	0.10738500
C	-6.83093700	-2.09924500	-0.61136600
H	-5.64198700	-3.88878800	-0.68161800
H	-7.75569800	-0.16667100	-0.45717800
H	-7.75143800	-2.60670900	-0.86609700

C_6F_5I ···4-Methylpyridine

I	-0.19220300	-1.00289600	0.00001600
C	1.75797400	-0.14852200	0.00000600
C	2.89528200	-0.94511500	-0.00001500
C	1.94055800	1.22792600	0.00001900
C	4.16979500	-0.39775500	-0.00002300
C	3.20550800	1.79672600	0.00001200
C	4.32580600	0.97991100	-0.00000900
F	0.88772700	2.05918900	0.00003900
F	3.35334400	3.12647300	0.00002500
F	5.54767900	1.51695900	-0.00001600
F	5.24928300	-1.18784800	-0.00004300
F	2.79513600	-2.28226900	-0.00002900
O	-2.73537300	-1.98600800	0.00002700
N	-3.57283200	-0.97604000	0.00000900
C	-4.00362500	-0.44638100	1.17013600
C	-4.00358800	-0.44639100	-1.17013600
C	-4.87536100	0.62012200	1.18663300
H	-3.61281500	-0.92479800	2.05285700
C	-4.87532300	0.62011300	-1.18667000
H	-3.61274900	-0.92481500	-2.05284000
C	-5.33711500	1.19016400	-0.00002800
H	-5.19319000	1.00519300	2.14500000
H	-5.19312000	1.00517600	-2.14505000
C	-6.25737300	2.37457000	-0.00004600
H	-6.89157800	2.38332800	0.88506300
H	-5.67919700	3.30167600	-0.00001700
H	-6.89151900	2.38334700	-0.88519700

6 References

(1) Laurence, C.; Graton, J.; Berthelot, M.; El Ghomari, M. J., The Diiodine Basicity Scale: Toward a General Halogen-Bond Basicity Scale. *Chem. Eur. J.* **2011,** *17* (37), 10431–10444.

(2) Laurence;, C.; Gal, J.-F., *Lewis Basicity and Affinity Scales*. Wiley: UK, The Atrium, Southern Gate, Chichester, West Sussex, England, 2010.

(3) Ciancaleoni, G.; Bertani, R.; Rocchigiani, L.; Sgarbossa, P.; Zuccaccia, C.; Macchioni, A., Discriminating Halogen-Bonding from Other Noncovalent Interactions by a Combined NOE NMR/DFT Approach. *Chem. Eur. J.* **2015,** *21* (1), 440-447.

(4) Becke, A. D., Density-functional exchange-energy approximation with correct asymptotic behavior. *Physical Review A* **1988,** *38* (6), 3098-3100.

(5) Lee, C.; Yang, W.; Parr, R. G., Development of the Colle-Salvetti correlation-energy formula into a functional of the electron density. *Physical Review B* **1988,** *37* (2), 785-789.

(6) Grimme, S.; Antony, J.; Ehrlich, S.; Krieg, H., A consistent and accurate ab initio parametrization of density functional dispersion correction (DFT-D) for the 94 elements H-Pu. *J. Chem. Phys.* **2010,** *132* (15), 154104.

(7) Jr., T. H. D., Gaussian basis sets for use in correlated molecular calculations. I. The atoms boron through neon and hydrogen. *J. Chem. Phys.* **1989,** *90* (2), 1007-1023.

(8) Woon, D. E.; Jr., T. H. D., Gaussian basis sets for use in correlated molecular calculations. V. Core-valence basis sets for boron through neon. *J. Chem. Phys.* **1995,** *103* (11), 4572-4585.

(9) Peterson, K. A.; Figgen, D.; Goll, E.; Stoll, H.; Dolg, M., Systematically convergent basis sets with relativistic pseudopotentials. II. Small-core pseudopotentials and correlation consistent basis sets for the post-d group 16–18 elements. *J. Chem. Phys.* **2003,** *119* (21), 11113-11123.

(10) Peterson, K. A., Systematically convergent basis sets with relativistic pseudopotentials. I. Correlation consistent basis sets for the post-d group 13–15 elements. *The J. Chem. Phys.* **2003,** *119* (21), 11099-11112.

(11) DiLabio, G. A.; Johnson, E. R.; Otero-de-la-Roza, A., Performance of conventional and dispersion-corrected density-functional theory methods for hydrogen bonding interaction energies. *PCCP* **2013,** *15* (31), 12821-12828.

(12) Torres, E.; DiLabio, G. A., A (Nearly) Universally Applicable Method for Modeling Noncovalent Interactions Using B3LYP. *J. Phys. Chem. Lett.* **2012,** *3* (13), 1738-1744.

(13) Tomasi, J.; Mennucci, B.; Cammi, R., Quantum Mechanical Continuum Solvation Models. *Chem. Rev.* **2005,** *105* (8), 2999-3094.

(14) Frisch, M. J.; Trucks, G. W.; Schlegel, H. B.; Scuseria, G. E.; Robb, M. A.; Cheeseman, J. R.; Scalmani, G.; Barone, V.; Petersson, G. A.; Nakatsuji, H.; Li, X.; Caricato, M.; Marenich, A. V.; Bloino, J.; Janesko, B. G.; Gomperts, R.; Mennucci, B.; Hratchian, H. P.; Ortiz, J. V.; Izmaylov, A. F.; Sonnenberg, J. L.; Williams; Ding, F.; Lipparini, F.; Egidi, F.; Goings, J.; Peng, B.; Petrone, A.; Henderson, T.; Ranasinghe, D.; Zakrzewski, V. G.; Gao, J.; Rega, N.; Zheng, G.; Liang, W.; Hada, M.; Ehara, M.; Toyota, K.; Fukuda, R.; Hasegawa, J.; Ishida, M.; Nakajima, T.; Honda, Y.; Kitao, O.; Nakai, H.; Vreven, T.; Throssell, K.; Montgomery Jr., J. A.; Peralta, J. E.; Ogliaro, F.; Bearpark, M. J.; Heyd, J. J.; Brothers, E. N.; Kudin, K. N.; Staroverov, V. N.; Keith, T. A.; Kobayashi, R.; Normand, J.; Raghavachari, K.; Rendell, A. P.; Burant, J. C.; Iyengar, S. S.; Tomasi, J.; Cossi, M.; Millam, J. M.; Klene, M.; Adamo, C.; Cammi, R.; Ochterski, J. W.; Martin, R. L.; Morokuma, K.; Farkas, O.; Foresman, J. B.; Fox, D. J. *Gaussian 16 Rev. C.01*, Wallingford, CT, 2016.

(15) Gräfenstein, J.; Cremer, D., Efficient density-functional theory integrations by locally augmented radial grids. *J. Chem. Phys.* **2007,** *127* (16), 164113.

(16) Glendening, E. D.; Badenhoop, J. K.; Reed, A. E.; Carpenter, J. E.; Bohmann, J. A.; Morales, C. M.; Karifiloglou, P.; Landis, C. R.; Weinhold, F. *NBO 7.0*, Theoretical Chemistry Institute, University of Wisconsin, Madison, 2018.

(17) Keith, T. A. *AIMALL (Version 19.10.12)*, TK Gristmill Software: Overland Park KS, USA, 2019.

(18) Cremer, D.; Kraka, E., Chemical Bonds without Bonding Electron Density — Does the Difference Electron-Density Analysis Suffice for a Description of the Chemical Bond? *Angew. Chem. Int. Ed.* **1984,** *23* (8), 627-628.
